# Benzylated Dihydroflavones and Isoquinoline-Derived Alkaloids from the Bark of *Diclinanona calycina* (Annonaceae) and Their Cytotoxicities

**DOI:** 10.3390/molecules26123714

**Published:** 2021-06-18

**Authors:** Emmanoel V. Costa, Liviane do N. Soares, Jamal da Silva Chaar, Valdenizia R. Silva, Luciano de S. Santos, Hector H. F. Koolen, Felipe M. A. da Silva, Josean F. Tavares, Gokhan Zengin, Milena B. P. Soares, Daniel P. Bezerra

**Affiliations:** 1 Department of Chemistry, Federal University of Amazonas (UFAM), Manaus 69080-900, AM, Brazil; BeM.Liviane@gmail.com (L.d.N.S.); jchaar@ufam.edu.br (J.d.S.C.); felipemourams@gmail.com (F.M.A.d.S.); 2 Gonçalo Moniz Institute, Oswaldo Cruz Foundation (IGM-FIOCRUZ/BA), Salvador 40296-710, BA, Brazil; valdeniziar@gmail.com (V.R.S.); luciano.biomed@gmail.com (L.d.S.S.); milenabpsoares@gmail.com (M.B.P.S.); 3 Metabolomics and Mass Spectrometry Research Group, Amazonas State University (UEA), Manaus 690065-130, AM, Brazil; hkoolen@uea.edu.br; 4 Health Sciences Center, Postgraduate Program in Natural and Synthetic Bioactive Products, Federal University of Paraíba (UFPB), João Pessoa 58051-970, PA, Brazil; josean@ltf.ufpb.br; 5 Department of Biology, Science Faculty, Selcuk University, 42130 Konya, Turkey; gokhanzengin@selcuk.edu.tr

**Keywords:** *Diclinanona calycina*, alkaloids and benzylated dihydroflavones, cytotoxic activity

## Abstract

*Diclinanona calycina* R. E. Fries popularly known as “envira”, is a species of the Annonaceae family endemic to Brazil. In our ongoing search for bioactive compounds from Annonaceae Amazon plants, the bark of *D. calycina* was investigated by classical chromatography techniques that yielded thirteen compounds (alkaloids and flavonoids) described for the first time in *D. calycina* as well as in the genus *Diclinanona*. The structure of these isolated compounds were established by extensive analysis using 1D/2D-NMR spectroscopy in combination with MS. The isolated alkaloids were identified as belonging to the subclasses: simple isoquinoline, thalifoline (**1**); aporphine, anonaine (**2**); oxoaporphine, liriodenine (**3**); benzyltetrahydroisoquinolines, (*S*)-(+)-reticuline (**4**); dehydro-oxonorreticuline (3,4-dihydro-7-hydroxy-6-methoxy-1-isoquinolinyl)(3-hydroxy-4-methoxyphenyl)-methanone) (**5**); (+)-1*S*,2*R*-reticuline *N*_β_-oxide (**6**); and (+)-1*S*,2*S*-reticuline *N*_α_-oxide (**7**); tetrahydroprotoberberine, coreximine (**8**); and pavine, bisnorargemonine (**9**). While the flavonoids belong to the benzylated dihydroflavones, isochamanetin (**10**), dichamanetin (**11**), and a mixture of uvarinol (**12**) and isouvarinol (**13**). Compound **5** is described for the first time in the literature as a natural product. The cytotoxic activity of the main isolated compounds was evaluated against cancer and non-cancerous cell lines. Among the tested compounds, the most promising results were found for the benzylated dihydroflavones dichamanetin (**10**), and the mixture of uvarinol (**12**) and isouvarinol (**13**), which presented moderate cytotoxic activity against the tested cancer cell lines (<20.0 µg·mL^−1^) and low cytotoxicity against the non-cancerous cell line MRC-5 (>25.0 µg·mL^−1^). Dichamanetin (**11**) showed cytotoxic activity against HL-60 and HCT116 with IC_50_ values of 15.78 µg·mL^−1^ (33.70 µmol·L^−1^) and 18.99 µg·mL^−1^ (40.56 µmol·L^−1^), respectively while the mixture of uvarinol (**12**) and isouvarinol (**13**) demonstrated cytotoxic activity against HL-60, with an IC_50_ value of 9.74 µg·mL^−1^, and HCT116, with an IC_50_ value of 17.31 µg·mL^−1^. These cytotoxic activities can be attributed to the presence of one or more hydroxybenzyl groups present in these molecules as well as the position in which these groups are linked. The cytotoxic activities of reticuline, anonaine and liriodenine have been previously established, with liriodenine being the most potent compound.

## 1. Introduction

Annonaceae is a large family of tropical and subtropical trees and shrubs, comprising about 112 genera and 2440 species [1]. Several species are known for their edible fruits and medicinal properties [2]. The previous phytochemical investigation with some species of Annonaceae led to the isolation and characterization of different classes of secondary metabolites, such as monoterpenes, diterpenes, triterpenes, lignans, flavonoids, asarone-derived phenylpropanoids, acetogenins and mainly typical isoquinoline-derived alkaloids [3,4,5,6,7]. Some of these secondary metabolites isolated from Annonaceae species exhibited important biological activities, such as anti-inflammatory and urease-inhibiting properties [8,9], trypanocidal [10,11], leishmanicidal [11,12], antimalarial [4,13], antimicrobial [4,14,15], antioxidant and antirheumatic actions [9,15] and, particularly, cytotoxic activity against different human tumor cell lines [4,6,11,16,17,18,19,20,21].

Although the Annonaceae family is considered a primitive and well-studied family, few phytochemical and/or pharmacological studies have been carried out with its species [3]. Phytochemical and/or pharmacological studies have focused mainly on species of the genera *Annona*, *Asimina* and *Cananga,* due to their great economic importance, and on some species of the genera *Duguetia*, *Guatteria* and *Xylopia* [5]. Despite the great growth in the last 20 years in relation to phytochemical and pharmacological studies, the number of species investigated is still very small in relation to the large number of recognized species. Currently, according to the *Web of Science*, *Scopus*, and *SciFinder* scientific databases, only about 15% of the Annonaceae species described have any corresponding phytochemical and/or pharmacological study.

Among the little studied species are those belonging to the genus *Diclinanona* Diels. This genus belongs to the tribe *Annoneae*, of the subfamily *Annonoideae*, and occurs only in tropical South America (mainly in the Amazon region). It is a genus consisting of only three species, *Diclinanona calycina* (Diels) R. E. Fries *Diclinanona matogrossensis* Maas and *Diclinanona tessmannii* Diels, which occur as trees [22,23,24]. *D. calycina* (synonymy *Xylopia calycina* Diels) is an 8 to 30 m tall tree, popularly known as “envireira” and “envira”, distributed throughout the Amazon basin in Brazil, Peru and Venezuela [23]. *D. calycina* is superficially similar to *Xylopia* for its flowers with elongate and narrow petals, but it is different for its woody, indehiscent, globose and thick-walled monocarps [23,24].

Previous studies with *D. calycina* report only pharmacological studies. The first study describes the investigation of the antimicrobial activity of methanolic, chloroform and aqueous extracts against the microorganisms *Mycobacterium smegmatis*, *Escherichia coli*, *Streptococcus sanguis*, *Streptococcus oralis*, *Staphylococcus aureus* and *Candida albicans* using the gel-diffusion method [25]. The second one reports the investigation of the antimicrobial activity of organic (dichloromethane:methanol 1:1) and aqueous extracts against the microorganism *Enterococcus faecalis* using the microdilution broth assay (MDBA) and disk diffusion assay (DDA) [26]. Thus, in our continuous search for new bioactive natural products from Annonaceae from Amazon rainforest, this study aimed to investigate the phytochemical and pharmacological properties of the bark of *D. calycina*. In this report, thirteen compounds (nine alkaloids and four benzylated dihydroflavones) were isolated and identified for the first time in *D. calycina,* as well as in the genus *Diclinanona*. In addition, the cytotoxicity of the main compounds was investigated against B16-F10, HepG2, K562, and HL-60 tumor cell lines using the Alamar blue assay.

## 2. Results and Discussion

### 2.1. Structural Elucidation of the Compounds

**Figure 1:**
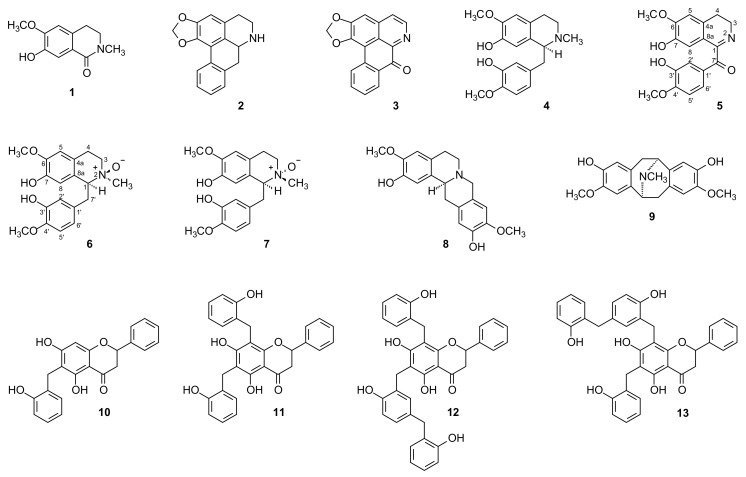
Chemical structures of the isolated compounds from the bark of *D. calycina*.

Having discovered the presence of nitrogen-containing compounds in the methanolic extract using Dragendorff’s reagent, it was subjected to acid-base treatment according to the methodology of Costa et al. [12] resulting in alkaloidal and neutral fractions. A high concentration of nitrogen-containing compounds was observed in the alkaloidal fraction that was subjected to chromatographic analysis. The successive chromatographic separations, as described in the Extraction and Isolation section, led to the isolation and identification of thirteen chemical constituents (**1**−**13**, Figure 1), nine isoquinoline-derived alkaloids **1**−**9** and four benzylated dihydroflavones **10**−**13**. The structures of these isolated compounds (Figure 1) were established by extensive analysis using 1D and 2D NMR spectroscopy in combination with MS (Supplementary Data), as well as comparison with data from the literature.

Compound **5** was obtained as a brown amorphous powder and tested positive for Dragendorff’s reagent. It showed a protonated molecule at *m/z* 328 [M + H]^+^ in the LR-ESI (+)MS compatible with the molecular formula C_18_H_17_NO_5_. The ^1^H and ^13^C-NMR spectra of **5** (Table 1) were consistent with those of reticuline (**4**) [27], except for the absence of the nitrogen-bonded methyl group (CH_3_−*N*) and the signal of the methine group in position 1, which was replaced by a signal at δ_C_ 165.1 in the typical ^13^C-NMR spectrum of the imine group conjugated to a carbonyl group at δ_C_ 192.8. In the ^1^H-NMR spectrum, three downfield hydrogens at δ_H_ 6.88 (1H, d, *J* = 8.4 Hz, H-5′), δ_H_ 7.57 (1H, d, *J* = 2.0 Hz, H-2′) and δ_H_ 7.60 (1H, dd, *J* = 8.4 and 2.0 Hz, H-6′), revealed the presence of an ABX coupling system. Two singlet signals at δ_H_ 6.90 (1H, s, H-8) and 6.71 (1H, s, H-5) indicated the presence of **1**,**2**,**4**,**5**-tetrasubstituted phenyl ring. In addition, the ^1^H-NMR spectrum of **5** also showed signals for two methoxy groups resonating at δ_H_ 3.93 (3H, s) and δ_H_ 3.95 (3H, s), and signals for two methylene groups resonating at δ_H_ 2.79 (2H, t, *J* = 7.8 Hz) and δ_H_ 3.89 (2H, t, *J* = 7.8 Hz), attributed to H-4 and H-3, respectively (Table 1). These data indicated that alkaloid **5** has a benzyltetrahydroisoquinoline skeleton [28,29,30].

**Figure 2:**
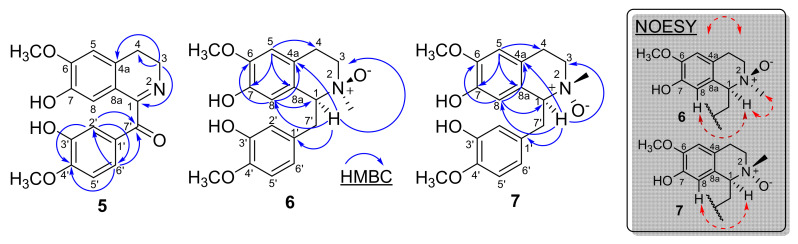
The key HMBC and NOESY correlations in alkaloids **5**–**7**.

These groups were established based on the two and three-bond ^1^H-^13^C correlation map from HMBC-NMR experiment (Figure 2 and Table 1). This analysis revealed that the hydrogen at δ_H_ 3.89 (H-3) showed three-bond ^1^H-^13^C correlation with the carbons at δ_C_ 130.1 (4a) and δ_C_ 165.0 (C-1) and two-bond ^1^H-^13^C correlation with the carbon at δ_C_ 25.4 (C-4), confirming the presence of imine group in the molecule. On the other hand, the signals at δ_H_ 7.57 (H-2′) and δ_H_ 7.60 (H-6′) showed three-bond ^1^H-^13^C correlation with the carbons at δ_C_ 124.3 (C-6′), δ_C_ 151,4 (C-4′) and δ_C_ 192.8 (C-7′), and δ_C_ 116.0 (C-2′), δ_C_ 151.4 (C-4′) and δ_C_ 192.8 (C-7′), respectively, establishing the carbonyl group in the molecule (Figure 2 and Table 1). Therefore, based on these NMR data, compound **5** was established as the benzyltetrahydroisoquinoline alkaloid 3,4-dihydro-7-hydroxy-6-methoxy-1-isoquinolinyl)(3-hydroxy-4-methoxyphenyl)-methanone, which was named as dehydro-oxonorreticuline. This alkaloid is described for the first time in the literature as a natural product. Its first and only record was described by Dörnyei et al. in 1982 [31] as a product of synthetic origin. Only the ^1^H-NMR data are described with some undetermined multiplicities. Thus, the complete assignments for all ^1^H- and ^13^C-NMR chemical shifts were established by one-bond (HSQC) and two and three-bond (HMBC) ^1^H-^13^C-NMR correlation experiments, and were described in the Table 1.

Compounds **6** and **7** were identified as the benzyltetrahydroisoquinoline alkaloids (+)-1*S*,2*R*-reticuline-*N*_β_-oxide and (+)-1*S*,2*S-*reticuline-*N*_α_-oxide, respectively. The ^1^H- NMR data of these alkaloids were compared with the data described by Lee et al. [28] using the same deuterated solvent (CD_3_OD) and some inconsistencies were observed (Table 1), mainly in relation to H-1, H-8, and H_3_C-*N*O. There are no ^13^C-NMR data described for these alkaloids in the literature. Based on the limited ^1^H- and ^13^C-NMR data, as well as the ambiguities observed for these molecules, ^1^H and ^13^C 1D and 2D NMR experiments were performed to determine their correct assignments and multiplicities.

The correct position of the hydrogen H-8 and H-1 of both isomers **6** and **7** was established based on the three-bond ^1^H-^13^C correlation map from HMBC-NMR experiment (Figure 2). For the isomer **6** the analysis revealed that the hydrogen at δ_H_ 5.78 (H-8) showed three-bond ^1^H-^13^C correlation with the carbons at δ_C_ 79.8 (C-1), δ_C_ 120.6 (4a), and δ_C_ 149.4 (C-6), while the hydrogen at δ_H_ 4,13 (H-1) showed three-bond ^1^H-^13^C correlation with the carbons at δ_C_ 60.7 (C-3), δ_C_ 115.7 (C-8), δ_C_ 120.6 (C-4a), and 131.2 (C-1′), thus establishing the correct positions of the hydrogens H-8 and H-1 for isomer **6**. This attribution can be further confirmed by the analysis of the hydrogen at δ_H_ 6.76 (H-5) that showed three-bond ^1^H-^13^C correlation with the carbons at δ_C_ 27.0 (C-4), δ_C_ 127.1 (C-8a), and δ_C_ 145.7 (C-7) (Figure 2). For the isomer **7** the analysis revealed that the hydrogen at δ_H_ 6.30 (H-8) showed three-bond ^1^H-^13^C correlation with the carbons at δ_C_ 79.4 (C-1), δ_C_ 122.8 (4a), and δ_C_ 148.9 (C-6), while the hydrogen at δ_H_ 4.46 (H-1) showed three-bond ^1^H-^13^C correlations with the carbons at δ_C_ 62.9 (C-3), δ_C_ 115.5 (C-8), δ_C_ 122.8 (C-4a), and 131.4 (C-1′), thus establishing the correct positions of the hydrogens H-8 and H-1 for isomer **7**. This attribution was also confirmed by the analysis of the hydrogen at δ_H_ 6.71 (H-5) that showed three-bond ^1^H-^13^C correlations with the carbons at δ_C_ 26.3 (C-4), δ_C_ 126.9 (C-8a), and δ_C_ 146.1 (C-7) (Figure 2). The NOESY experiment was also carried out to establish the correct stereochemistry of isomers **6** and **7**. In this experiment the strong NOE correlation of H-1 (δ_H_ 4.13) and H_3_C-*N* (δ_H_ 3.15) indicated the β-orientation of the oxygen in the isomer **6**. On the other hand, no obvious NOE correlation between H-1 (δ_H_ 4.46) and H_3_C-*N* (δ_H_ 3.20) could be found in the 2D-NOESY experiment for **7**, indicated the α-orientation of the oxygen in the isomer **7** (Figure 2). These small differences in the chemical shifts of these isomers are clearly observed due to the stereochemistry of the nitrogen affected by the α and β position of oxygen. Comparisons of the ^1^H- and ^13^C-NMR data obtained for compounds **6** and **7** with data of molecules with close structures such as hexapetaline A and hexapetaline B [30] support the data described in Table 1 without ambiguity.

Compounds **1**−**4** and **8**−**13** were identified as thalifoline (**1**) [32], anonaine (**2**) [33], liriodenine (**3**) [10,34], (*S*)-(+)-reticuline (**4**) [27], coreximine (**8**) [34,35], bisnorargemonine (**9**) [36], isochamanetin (**10**) [37], dichamanetin (**11**) [37] and an a mixture of uvarinol (**12**) and isouvarinol (**13**) [37] based on their spectroscopic profiles and comparison with values in the literature. The ^1^H- and ^13^C 1D and 2D-NMR spectra, as well as the mass spectra of all isolated compounds, are available as Supplementary Materials.

From a chemophenetic (a new term for plant chemosystematics/plant chemotaxonomy) point of view, it is important to note that the presence of *C*-benzylated flavanones and *C*-benzylated dihydrochalcones are a special type of flavonoids derived from the well-known flavanone pinocembin and have been described particularly in species of the genus *Uvaria* belonging to the Annonaceae family [3,38,39,40,41,42]. The presence of these compounds in *D. calycina* suggest close chemophenetic relationships with *Uvaria*. On the other hand, further investigations should be carried out with other parts of the plant, as well as other species of *Diclinanona* to confirm this chemophenetic relationship. Flavanones and chalcones are widespread in the higher plants, but the addition of benzyl groups is quite rare and seems to be limited in the Annonaceae to the genus *Uvaria* [3,39,40,41,42] and now to the genus *Diclinanona*. Benzyl groups presumably arise from a C_6_−C_1_ pathway, but *o*-hydroxy functionality is unusual. The absence of substituents in B-ring in all these flavonoids of *Uvaria* and *Diclinanona* can be linked with the previous observation concerning the flavonoids of *Popowia cauliflora* [38].

The isoquinoline-derived alkaloids isolated and described in this work have already been registered in several species of Annonaceae in different genera. Some of these, such as liriodenine and anonaine, are considered chemophenetic markers, and the presence of these alkaloids in *D. calycina* further reinforces the relationship of these chemophenetic markers in the Annonaceae family [5,6,7,15,20,27,33,34,35,43].

It is worth mentioning that, according to Zidorn [44], the chemophenetic studies are defined as studies aimed to describe the array of specialized secondary metabolites in a given taxon, as already observed in several published works [5,6,7,15,20,27,33,34,35,43,44,45,46]. Thus, chemophenetic studies contribute to the phenetic description of taxa, similar to anatomical, morphological and karyological approaches, which have already been recognized as of great importance for the establishment “natural” systems, and which continue to be of extreme importance for the description of classified organisms with the help of modern molecular methods [44].

### 2.2. Cytotoxic Assay

The in vitro cytotoxic activity of the isolated compounds (Table 2), except for compound **2**, **3**, **5**, and **8** (due to their low yield), was evaluated against cancer cell lines HL-60 (human promyelocytic leukemia), MCF-7 (human breast adenocarcinoma), HepG2 (human hepatocellular carcinoma), HCT116 (human colon carcinoma), and non-cancerous cell line MRC-5 (human lung fibroblast) using the Alamar blue assay after 72 h of incubation.

Among the compounds evaluated (Table 2), the most promising results were verified for benzylated dihydroflavones dichamanetin (**10**), and the mixture of uvarinol (**12**) and isouvarinol (**13**), which showed moderate cytotoxic activity against the tested cancer cell lines and low cytotoxicity against the non-cancerous cell line MRC-5 (>25.0 µg·mL^−1^). Dichamanetin (**11**) showed cytotoxic activity against HL-60 and HCT116 with IC_50_ values of 15.78 µg·mL^−1^ (33.70 µmol·L^−1^) and 18.99 µg·mL^−1^ (40.56 µmol·L^−1^), respectively. The mixture of uvarinol (**12**) and isouvarinol (**13**) demonstrated cytotoxic activity against HL-60, with IC_50_ value of 9.74 µg·mL^−^^1^, and HCT116, with IC_50_ value of 17.31 µg·mL^−1^. Among the benzylated dihydroflavones, only isochamanetin (**10**) showed cytotoxic activity against HepG2 with IC_50_ value of 19.79 µg·mL^−1^ (54.65 µmol·L^−1^). According to the literature, benzylated dihydroflavones are described with cytotoxic properties [39,40,41,42]. Among those described in this work, isochamanetin (**10**), dichamanetin (**11**), and uvarinol (**12**) are described with in vitro cytotoxic properties against human tumor cell lines of carcinoma of the nasopharynx (KB) and P-388 lymphocytic leukemia (PS) with IC_50_ values of 5.3 and 4.1, 4.8 and 1.8, and 5.9 and 9.7, µg·mL^−1^, respectively [39,40]. These different activities can be attributed to the presence of one or more hydroxybenzyl groups present in the molecules as well as the position in which these groups are linked. However, further investigation is needed to confirm this observation.

The in vitro cytotoxic activity of the isoquinoline-derived alkaloids anonaine (**2**), liriodenine (**3**) and (*S*)-(+)-reticuline (**4**) has been recently described by Menezes et al. [20], Souza et al. [6] and Costa et al. [43] with emphasis on liriodenine, which demonstrated potent cytotoxic activity against cancer cell lines B16-F10 (mouse melanoma), HepG2 (human hepatocellular carcinoma), HL-60 (human promyelocytic leukemia), and K562 (human chronic myelocytic leukemia) with IC_50_ values below to 10.0 µmol·L^−1^ [43]. Anonaine showed moderate activity against B16-F10, HepG2, HL60, and K562 with IC_50_ values of low then 19.0 µmol·L^−1^ [20]. (*S*)-(+)-Reticuline showed moderate activity only against HepG2 with IC_50_ values of 15.35 µmol L^−1^ [6,20].

## 3. Materials and Methods 

### 3.1. General Experimental Procedures

Optical rotations in methanol (MeOH) were recorded with a P-2000 polarimeter (Jasco, Tokyo, Japan) at 589 nm. 1D and 2D NMR experiments were acquired in CDCl_3_ (chloroform-*d*) or CDCl_3_ plus drop of CD_3_OD (methanol-*d_4_*, and CD_3_COCD_3_ (acetone*-d_6_*) at 298 K on an AVANCE III HD NMR spectrometer (Bruker, Billerica, MA, USA) operating at 11.75 T (^1^H and ^13^C at 500 and 125 MHz, respectively) and on a Bruker AVANCE III 600 NMR spectrometer operating at 14.1 T (^1^H and ^13^C at 600 and 150 MHz respectively). All ^1^H- and ^13^C-NMR chemical shifts (δ) are presented in ppm relative to the tetramethylsilane signal at 0.00 ppm as an internal reference, and the coupling constants (*J*) are given in Hz. The NMR spectrometer was equipped with a 5-mm multinuclear inverse detection probe (1D and 2D NMR experiments) with z-gradient. One-bond (HSQC) and two and three-bond (HMBC) ^1^H-^13^C-NMR correlation experiments were optimized for average coupling constant ^1^*J*_(C,H)_ and ^LR^*J*_(C,H)_ of 140 and 8 Hz, respectively. For low resolution mass spectrometry (LR-MS) analysis the samples of the isolated compounds were resuspended in methanol (HPLC grade), creating the stock solutions (1 mg·mL^−1^). Aliquots (5 µL) of the stock solutions were further diluted to 5 µg·mL^−1^ and analyzed by direct infusion into a triple quadrupole mass spectrometer, model TSQ Quantum Access (Thermo Scientific, San Jose, CA, USA), equipped with electrospray ionization (ESI) or atmospheric pressure chemical ionization (APCI) sources. ESI-MS conditions: spray voltage, 5 kV; sheath gas, 10 arbitrary unit (arb); auxiliary gas, 5 arb; sweep gas, 0 arb; capillary temp, 250 °C; capillary voltage, 40 V; tube lens, 70 V; mass range, *m/z* 100 to 1000. APCI-MS conditions: discharge current, 5 µA; vaporizer temperature, 350 °C; sheath gas pressure, 25 arbitrary unit (arb); ion sweep gas pressure, 0.0 arb; aux gas pressure, 10 arb; capillary temperature, 250 °C; tube lens offset, 70 V; skimmer offset, 0 V; mass range, *m/z* 100 to 1000. Argon was used as collision gas, and the MS/MS spectra were obtained using collision energies ranging from 25 to 30 eV. Silica gel 60 (Sigma-Aldrich, San Luis, MO, USA, 70−230 mesh) was used for the column chromatography (CC), while silica gel 60 F_254_ (Macherey-Nagel, Düren, Germany, 0.25 mm, aluminum) was used for analytical and preparative with thin layer chromatography (PTLC) (Macherey-Nagel, 1.00 mm, glass). Compounds were visualized by exposure under UV_254/365_ light, by spraying with *p*-anisaldehyde reagent followed by heating on a hot plate, and by spraying Dragendorff’s reagent.

### 3.2. Plant Material

In the present investigation, the botanical material (bark) of *D. calycina* was collected in 27 May 2017 on the Adolpho Ducke Reserve (geographic coordinates: 02°53′36.1′′ S and 59°58′28.9′′ W), Manaus, Amazonas State, Brazil, and identified by Prof. Dr. Antonio Carlos Webber, a plant taxonomist of the Department of Biology of the Universidade Federal do Amazonas (DB/UFAM). A voucher specimen number 10,810 was deposited at the Herbarium of DB/UFAM. The access (specimen) was registered in the Sistema Nacional de Gestão do Patrimônio Genético e do Conhecimento Tradicional Associado (SISGEN) with the record A70EDCD.

### 3.3. Extraction and Isolation

The bark of *D. calycina* was initially dried at room temperature for 24 h and then dried in an air-circulating oven for 48 h at a temperature of 40 °C, and subsequently pulverized in a four-knife mill grinder (Marconi, Piracicaba, SP, Brazil) to obtain the powdered material (1340 g). Then, an exhaustive maceration with hexane (8 × 4 L) followed by MeOH (8 × 4 L) was performed. The extractive solutions obtained were concentrated in a rotary evaporator at reduced pressure (40−50 °C) to give the hexane (24.85 g) and MeOH (199.43 g) extracts, respectively.

TLC analysis revealed with Dragendorff’s reagent indicated a high presence of alkaloids in the MeOH extract. Therefore, an aliquot of MeOH extract (188.30 g) was initially subjected to an acid−base extraction to give alkaloidal (5.46 g) and neutral (4.47 g) fractions. Subsequently, part of alkaloidal fraction (5.0 g) was subjected to silica gel chromatographic column (CC) previously treated with a 10% NaHCO_3_ solution [12], eluted with hexane (100%), hexane−CH_2_Cl_2_ (90:10, 80:20, 70:30, 60:40, 50:50, 40:60, 30:70, 20:80, and 10:90, *v/v*), CH_2_Cl_2_ (100%), CH_2_Cl_2_−EtOAc (90:10, 80:20, 70:30, 60:40, 50:50, 40:60, 30:70, 20:80, and 10:90, *v/v*), EtOAc (100%), EtOAc−MeOH (95:05, 90:10, 85:05, 80:10, 75:25, 70:30, 60:40, 50:50, 40:60, 30:70, 20:80, and 10:90, *v/v*), and finally MeOH giving 250 fractions (30 mL each). After TLC evaluation using a mixture of CH_2_Cl_2_−MeOH in the proportions of 95:05, 90:10, 85:15, and 80:20 as the eluent system (*v/v*), the similar samples were pooled to give 16 fractions (F1 to F16).

Fraction F5 (181.2 mg) from initial CC eluted with hexane−CH_2_Cl_2_ (50:50 to 10:90, *v/v*) was subjected to a new silica gel CC using the same methodology above eluted with hexane (100%), hexane−CH_2_Cl_2_ (90:10, 80:20, 70:30, 60:40, 50:50, 40:60, 30:70, 20:80, and 10:90, *v/v*), CH_2_Cl_2_ (100%), CH_2_Cl_2_−EtOAc (90:10, 80:20, 70:30, 60:40, 50:50, 40:60, 30:70, 20:80, and 10:90, *v/v*), EtOAc (100%), EtOAc−MeOH (85:15, 70:30, 50:50, 30:70, and 15:85, *v/v*), and MeOH (100%) affording 118 fractions (15 mL each) that were pooled in 12 subfractions (F5.1 to F5.12), according to TLC analysis evaluation using a mixture of CH_2_Cl_2_−MeOH in the proportions of 95:05 and 90:10. Subfraction F5.1 (28.9 mg) eluted with hexane (100%) was subjected to a preparative TLC eluted with CH_2_Cl_2_−MeOH (95:05, *v/v*, two elutions) affording **2** (1.4 mg) and **3** (1.7 mg), respectively. Subfractions F5.4 (21.5 mg) eluted with hexane−CH_2_Cl_2_ (80:20, *v/v*), F5.5 (16.7 mg) eluted with hexane−CH_2_Cl_2_ (80:20, 70:30, 60:40, and 50:50, *v/v*), and F5.6 (15.4 mg) eluted with hexane−CH_2_Cl_2_ (50:50, 40:60, and 30:70, *v/v*) were pooled (53.6 mg) and also subjected to a preparative TLC eluted with CH_2_Cl_2_−MeOH (95:05, *v/v*, two elutions) yielding **3** (8.9 mg).

Fraction F6 (2099.1 mg) from initial CC eluted with hexane−CH_2_Cl_2_ (10:90, *v/v*), CH_2_Cl_2_ (100%), and CH_2_Cl_2_−EtOAc (90:10 to 10:90, *v/v*) was subjected to a new silica gel CC using the same methodology above eluted with the same solvents systems affording 140 fractions (30 mL each). After TLC evaluation using a mixture of CH_2_Cl_2_−MeOH in the proportions of 95:05, 90:10, and 85:15 as the eluent system (*v/v*), the similar samples were pooled to give 13 subfractions (F6.1 to F6.13). Subfraction F6.6 (1541.3 mg) was subjected to a new silica gel CC using the same methodology above eluted with the same solvents systems affording 120 fractions (30 mL each) that were analyzed by TLC (using the same methodology above) providing 12 new subfractions (F6.6.1 to F6.6.12). Subfraction F6.6.2 (17.0 mg) eluted with hexane−CH_2_Cl_2_ (60:40 and 50:50, *v/v*) was subjected to a preparative TLC eluted with CH_2_Cl_2_−MeOH (90:10, *v/v*, one elution) resulting in **3** (1.4 mg). Subfraction F6.6.3 (643.3 mg) eluted with hexane−CH_2_Cl_2_ (50:50, 40:60, 30:70, 20:80, and 10:90, *v/v*), CH_2_Cl_2_ (100%), and CH_2_Cl_2_−EtOAc (90:10 and 80:20, *v/v*) was subjected to a preparative TLC eluted with CH_2_Cl_2_−MeOH (90:10, *v/v*, one elution) yielding a new subfraction F6.6.3.1 (161.8 mg) that was subjected to a new preparative TLC eluted with EtOAc−MeOH (90:10, *v/v*, one elution) affording **4** (148.2 mg). Subfraction F6.6.4 (90.2 mg) eluted with CH_2_Cl_2_−EtOAc (70:30 and 60:40, *v/v*) was subjected to a preparative TLC eluted with CH_2_Cl_2_−MeOH (90:10, *v/v*, one elution) yielding **1** (1.4 mg) and **4** (50.1 mg), respectively. Subfraction F6.6.5 (83.4 mg) eluted with CH_2_Cl_2_−EtOAc (60:40 and 50:50, *v/v*) was also subjected to a preparative TLC eluted with CH_2_Cl_2_−MeOH (90:10, *v/v*, one elution) affording again **1** (1.5 mg) and **4** (45.5 mg), respectively. Subfraction F6.6.6 (101.4 mg) eluted with CH_2_Cl_2_−EtOAc (50:50 and 40:60, *v/v*) was subjected to a preparative TLC eluted with CH_2_Cl_2_−MeOH (90:10, *v/v*, one elution) giving **8** (3.4 mg) and other subfraction F6.6.6.1 (48.8 mg). This subfraction (F6.6.6.1) was subjected to a preparative TLC eluted initially with EtOAc−MeOH (85:15, *v/v*, one elution) and posteriorly CH_2_Cl_2_−MeOH (90:10, *v/v*, one elution) resulting again in **4** (37.4 mg). For this procedure, the chromatographic plate was initially eluted with the mobile phase EtOAc−MeOH (85:15, *v/v*, one elution). After this elution, the chromatographic plate was dried to remove the solvent and subsequently subjected to a new elution with the mobile phase CH_2_Cl_2_−MeOH (90:10, *v/v*, one elution) yielding **4**. Subfraction F6.6.7 (109.0 mg) eluted with CH_2_Cl_2_−EtOAc (40:60, 30:70, 20:80 and 10:90, *v/v*) was subjected to a preparative TLC eluted with CH_2_Cl_2_−MeOH (90:10, *v/v*, one elution) giving another subfraction F6.6.7.1 (24.8 mg) that was subjected to a new preparative TLC eluted initially with EtOAc−MeOH (85:15, *v/v*, one elution) and posteriorly CH_2_Cl_2_−MeOH (90:10, *v/v*, one elution) resulting in **9** (11.1 mg). The isolation procedure used for **9** was the same as previously described for **4**. Subfraction F6.6.10 (92.0 mg) eluted with EtOAc−MeOH (60:40 and 50:50, *v/v*) was subjected to a preparative TLC eluted with CH_2_Cl_2_−MeOH (90:10, *v/v*, one elution) yielding **5** (1.7 mg) and **6** (7.8 mg), respectively. Subfraction F6.6.11 (326.0 mg) eluted with EtOAc−MeOH (50:50, 40:60, 30:70, 20:80, and 10:90, *v/v*) was subjected to a preparative TLC eluted with CH_2_Cl_2_−MeOH (90:10, *v/v*, one elution) yielding **1** (2.4 mg) and **7** (12.5 mg), respectively.

Fraction F7 (692.4 mg) from initial CC eluted with EtOAc (100%) and CH_2_Cl_2_−EtOAc (95:05, 90:10, 85:15, and 75:25, *v/v*) was subjected to a new silica gel CC using the same methodology as described for initial CC with the same solvents systems affording 110 fractions (30 mL each). After TLC evaluation using a mixture of CH_2_Cl_2_−MeOH in the proportions of 95:05, 90:10, and 85:15 as the eluent system (*v/v*), the similar samples were pooled to give 12 subfractions (F7.1 to F7.12). F7.8 (147.4 mg) eluted with CH_2_Cl_2_−EtOAc (50:50, 40:60, and 30:70, *v/v* %) was subjected to a preparative TLC eluted with CH_2_Cl_2_−MeOH (95:05, *v/v*, one elution) affording **10** (5.4 mg) and **11** (120.6 mg), respectively. F7.9 (144.2 mg) eluted with CH_2_Cl_2_−EtOAc (30:70, 20:80, and 10:90, *v/v* %) was also subjected to a preparative TLC eluted with CH_2_Cl_2_−MeOH (95:05, *v/v*, one elution) affording **11** (91.2 mg) and a mixture of **12** and **13** (39.4 mg), respectively.

*Thalifoline* (**1**): Brown amorphous powder; ^1^H-NMR and ^13^C-NMR in accordance with literature [32]; LR-ESI(+)-MS [M + H]^+^ *m/z* 208.

*Anonaine* (**2**): Brown amorphous powder; ^1^H-NMR and ^13^C-NMR in accordance with literature [33]; LR-ESI(+)-MS [M + H]^+^ *m/z* 266.

*Liriodenine* (**3**): Yellow crystals (CH_2_Cl_2_−MeOH 3:1); ^1^H-NMR and ^13^C-NMR in accordance with literature [10,34]; LR-ESI(+)-MS [M + H]^+^ *m/z* 276.

*(S)-(+)-Reticuline* (**4**): Brown amorphous powder; [α]_D_^25^ +71,60 (*c* 0.05 g/100 mL, MeOH); ^1^H-NMR and ^13^C-NMR in accordance with literature [27]; LR-ESI(+)-MS [M + H]^+^ *m/z* 330.

*3,4-Dihydro-7-hydroxy-6-methoxy-1-isoquinolinyl)(3-hydroxy-4-methoxyphenyl)-methanone (Dehydro-oxonorreticuline)* (**5**): Brown amorphous powder; ^1^H and ^13^C-NMR data, see Table 1; LR-ESI(+)-MS [M + H]^+^ m/z 328.

*(+)-1S,2R-Reticuline N*_β_*-oxide* (**6**): Brown amorphous powder; [α]_D_^25^ +136.4 (*c* 0.146 g/100 mL, MeOH); ^1^H and ^13^C-NMR data, see Table 1; LR-APCI(+)-MS [M + H]^+^ *m/z* 346.

*(+)-1S,2S-Reticuline N*_α_*-oxide* (**7**): Brown amorphous powder; [α]_D_^25^ +394.5 (*c* 0.03 g/100 mL, MeOH); ^1^H and ^13^C-NMR data, see Table 1; LR-APCI(+)-MS [M + H]^+^ *m/z* 346.

*Coreximine* (**8**): Light yellow amorphous powder; [α]_D_^25^ +201.1 (*c* 0.08 g/100 mL, MeOH) ^1^H-NMR and ^13^C-NMR in accordance with literature [34,35]; LR-ESI(+)-MS [M + H]^+^ *m/z* 328.

*Bisnorargemonine* (**9**): Brown amorphous powder; ^1^H-NMR and ^13^C-NMR in accordance with literature [36]; LR-APCI(+)-MS [M + H]^+^ *m/z* 328.

*Isochamanetin* (**10**): Light yellow amorphous powder; [α]_D_^25^ −12.4 (*c* 0.5 g/100 mL, MeOH) ^1^H-NMR and ^13^C-NMR in accordance with literature [37]; LR-APCI(−)-MS [M − H]^−^ *m/z* 361.

*Dichamanetin* (**11**): Yellow amorphous powder; [α]_D_^25^ −10.7 (*c* 0.5 g/100 mL, MeOH); ^1^H-NMR and ^13^C-NMR in accordance with literature [37]; LR-ESI(−)-MS [M − H]^−^ *m/z* 467.

Mixture of *uvarinol* (**12**) and *isouvarinol* (**13**): Yellow amorphous powder; ^1^H-NMR and ^13^C-NMR in accordance with literature [37]; LR-ESI(−)-MS [M − H]^−^ *m/z* 573.

### 3.4. In Vitro Cytotoxic Assay

#### 3.4.1. Cells

HL-60 (human promyelocytic leukemia), MCF-7 (human breast adenocarcinoma), HepG2 (human hepatocellular carcinoma), HCT116 (human colon carcinoma) and MRC-5 (human lung fibroblast) cell lines were obtained from American Type Culture Collection (ATCC, Manassas, VA, USA), and were cultured as recommended by ATCC animal cell culture guide. All cell lines were tested for mycoplasma using a mycoplasma stain kit (Sigma-Aldrich) to validate the use of cells free from contamination.

#### 3.4.2. Cytotoxicity Assay

For cytotoxicity assay, cell viability was quantified by Alamar blue method, as previously described [47,48,49]. For all experiments, cells were plated in 96-well plates. Chemical constituents were dissolved in dimethyl sulfoxide (DMSO, Vetec Química Fina Ltda., Duque de Caxias, RJ, Brazil) and added to each well and incubated for 72 h. Doxorubicin (doxorubicin hydrochloride, purity ≥ 95%, Laboratory IMA S.A.I.C., Buenos Aires, Argentina) was used as a positive control. At the end of treatment, 20 μL of a stock solution (0.312 mg/mL) of resazurin (Sigma-Aldrich Co.) was added to each well. Absorbances at 570 nm and 600 nm were measured using a SpectraMax 190 Microplate Reader (Molecular Devices, Sunnyvale, CA, USA). Half-inhibitory concentration (IC_50_) was obtained by nonlinear regression with 95% confidence intervals (CI 95%) using the software GraphPad Prism (Intuitive Software for Science; San Diego, CA, USA).

## 4. Conclusions

The phytochemical investigation of the bark of *D. calycina* led to the isolation and identification of thirteen compounds (**1**−**13**); nine isoquinoline-derived alkaloids (**1**−**9**) and four flavonoids (**10**−**13**). The alkaloids belongs to the following subclasses: simple isoquinoline, thalifoline (**1**); aporphine, anonaine (**2**); oxoaporphine, liriodenine (**3**); benzyltetrahydroisoquinolines, (+)-reticuline (**4**), *dehydro-oxonorreticuline* (3,4-dihydro-7-hydroxy-6-methoxy-1-isoquinolinyl)(3-hydroxy-4-methoxyphenyl)-methanone) (**5**), 1*S*,2*R*-reticuline *N*_β_-oxide (**6**), and 1*S*,2*S*-reticuline *N*_α_-oxide (**7**); tetrahydroprotoberberine, coreximine (**8**); and pavine, bisnorargemonine (**9**). While the flavonoids belong to the benzylated dihydroflavones, isochamanetin (**10**), dichamanetin (**11**), and a mixture of uvarinol (**12**) and isouvarinol (**13**). The isolated compounds are described for the first time in *D. calycina* as well as in the *Diclinanona* genus.

The cytotoxic activity of the isolated compounds (except for anonaine, liriodenine, 1,2-dihydro-oxonorreticuline and coreximine) were evaluated against cancer (HL-60, MCF-7, HepG2 and HCT116) and non-cancerous (MRC-5) cell lines. Among them, the most promising results were observed for benzylated dihydroflavones dichamanetin (**10**), and the mixture of uvarinol (**12**) and isouvarinol (**13**) that showed moderate cytotoxic activity against the cancer cell lines tested (<20.0 µg·mL^−1^) and low cytotoxicity against the non-cancerous cell line MRC-5 (>25.0 µg·mL^−1^). Dichamanetin (**11**) showed cytotoxic activity against HL-60 and HCT116 with IC_50_ values of 15.78 µg·mL^−1^ (33.70 µmol·L^−1^) and 18.99 µg·mL^−1^ (40.56 µmol·L^−1^), respectively while the mixture of uvarinol (**12**) and isouvarinol (**13**) demonstrated cytotoxic activity against HL-60, with IC_50_ value of 9.74 µg·mL^−^^1^, and HCT116, with IC_50_ value of 17.31 µg·mL^−1^. These results on the tumor cell lines is also describe for the first time for the benzylated dihydroflavones. The cytotoxic activities of reticuline, anonaine and liriodenine were previously established, in which liriodenine is the most potent compound.

The results obtained in this work indicate that the species of the family Annonaceae are a promising source of biologically active compounds with cytotoxic properties, and suggest the continuation of their investigation in other models of biological assays.

## Figures and Tables

**Table 1:** NMR data for alkaloids **5**–**7** (500 MHz for ^1^H and 125 MHz for ^13^C).

Position	5		6		6 ^e^	7		7 ^e^
	δ_C_ Mult. ^a,c,d^	δ_H_ Mult. ^a^ (*J* in Hz)	δ_C_ Mult. ^b,c,d^	δ_H_ Mult. ^b^ (*J* in Hz)	δ_H_ Mult.	δ_C_ Mult. ^b,c,d^	δ_H_ Mult. ^b^ (*J* in Hz)	δ_H_ Mult.
1	165.0		79.8	4.13 *br d* (*J* 10.4)	4.82 *br d*	79.4	4.46 *dd* (*J* 8.7; 2.9)	5.08 *dd*
3α 3β	47.2	3.89 *t* (*J* 7.8)	60.7	3.42 *m* 3.76 *m*	N.D.	62.9	3.48 *m* 3.67 *m*	N.D.
4α 4β	25.4	2.79 *t* (*J* 7.8)	27.0	3.14 *m*	N.D.	26.3	2.93 *dt* (*J* 17.2; 5.8) 3.21 *dt* (*J* 17.2; 7.3)	N.D.
4a	130.1		120.6			122.8		
5	109.9	6.71 *s*	112.5	6.76 *s*	6.79 *s*	112.3	6.71 *s*	6.77 *s*
6	149.0		149.4			148.9		
7	144.2		145.7			146.1		
8	113.0	6.90 *s*	115.7	5.78 *s*	5.75 *s*	115.5	6.30 *s*	6.05 *s*
8a	120.1		127.1			126.9		
1′	129.1		131.2			131.4		
2′	116.0	7.57 *d* (*J* 2.0)	118.0	6.58 *d* (*J* 2.2)	6.54 *d*	117.3	6.65 *d* (*J* 2.0)	6.63 *d*
3′	145.4		147.6			147.9		
4′	151.4		148.0			148.2		
5′	109.9	6.88 *d* (*J* 8.4)	112.8	6.81 *d* (*J* 8.2)	6.82 *d*	113.1	6.83 *d* (*J* 8.2)	6.85 *d*
6′	124.3	7.60 *dd* (*J* 8.4; 2.0)	122.5	6.47 *dd* (*J* 8.2; 2.2)	6.48 *br d*	121.6	6.60 *dd* (*J* 8.2; 2.0)	6.56 *br d*
7′α 7′β	192.8		38.9	4.04 *dd* (*J* 12.6; 2.5) 2.54 *dd* (*J* 12.6; 10.4)	N.D.	39.0	3.62 *dd* (*J* 14.2; 2.9) 2.79 *dd* (*J* 14.2; 8.7)	N.D.
H_3_CO-6	56.0	3.93 *s*	56.4	3.81 *s*	3.82	56.5	3.80 *s*	3.82 *s*
H_3_CO-4′	56.1	3.95 *s*	56.4	3.82 *s*	3.81	56.4	3.81 *s*	3.81 *s*
H_3_C-*N*O			56.3	3.15 *s*	3.46	54.6	3.20 *s*	3.59 *s*

^a,b^ The experiments were obtained in CDCl_3_^a^ or CD_3_OD ^b^ at 298 K and the NMR chemical shift are given in ppm related to TMS signal at 0.00 ppm as internal reference. ^c^ Multiplicities were determined by DEPT 135 and HSQC-NMR experiments. ^d^ The correct NMR chemical shifts of the carbon atoms were obtained through one-bond (HSQC), and two and three-bond ^1^H-^13^C (HMBC) NMR correlation experiments. ^e 1^H-NMR data (CD_3_OD, 400 MHz) according to Lee et al. [28]. N.D.: Not determinated.

**Table 2:** Cytotoxic activity of the isolated compounds from the bark of *D. calycina*.

Compounds	IC_50_ in µg·mL^−1^ (µmol·L^−1^) ^a^				
	HL-60	MCF-7	HepG2	HCT116	MRC-5
Thalifoline (**1**)	N.D	N.D	20.08 (96.96) 17.15−23.51	>25.0 (>120.72)	>25.0 (>120.72)
(*S*)-(+)-Reticuline (**4**)	N.D	N.D	22.54 (68.47) 17.39−29.21	>25.0 (>75.95)	>25.0 (>75.95)
**1***S*,**2***R*-Reticuline *N*_β_-oxide (**6**)	N.D	N.D	23.11 (66.95) 19.50−36.22	>25.0 (>72.43)	>25.0 (>72.43)
**1***S*,**2***S*-Reticuline *N*_α_-oxide (**7**)	N.D	N.D	>25.0 (>72.43)	>25.0 (>72.43)	>25.0 (>72.43)
Bisnorargemonine (**9**)	N.D	N.D	>25.0 (>72.43)	>25.0 (>72.43)	>25.0 (>72.43)
Isochamanetin (**10**)	N.D	N.D	19.79 (54.65) 8.46−26.28	>25.0 (>69.03)	24.69 (68.18) 17.84−27.52
Dichamanetin (**11**)	15.78 (33.70) 14.37−17.33	23.59 (50.38) 17.21−32.35	>25.0 (>53.40)	18.99 (40.56) 11.98−26.08	>25.0 (>53.40)
Mixture ^b^ of uvarinol (**12**) + isouvarinol (**13**)	9.74 7.90−12.01	>25.0	>25.0	17.31 14.87−20.14	>25.0
Doxorubicin ^c^	0.04 (0.07) 0.03–0.05	3.08 (5.67) 1.52–6.27	2.05 (3.77) 1.34–3.16	0.85 (1.56) 0.59−1.24	3.19 (5.87) 1.89−5.40

^a^ Data are presented as IC_50_ values, in µg·mL^−1^ (μmol·L^−1^) and their 95% confidence interval obtained by nonlinear regression from three independent experiments performed in duplicate, measured using Alamar blue assay after 72 h incubation. Cancer cells: HL-60 (human promyelocytic leukemia), MCF-7 (human breast adenocarcinoma), HepG2 (human hepatocellular carcinoma) and HCT116 (human colon carcinoma). Non-cancerous cell: MRC-5 (human lung fibroblast). ^b^ Since the compounds are in a mixture, their values in μmol·L^−1^ were not calculated. ^c^ Doxorubicin was used as a positive control. N.D.: Not determined.

## Data Availability

The data presented in this study are available in Supplementary Materials.

## Supplementary Materials

The following are available online. Figures S1–S123: ^1^H and ^13^C 1D and 2D NMR, and MS spectra for compounds **1**–**13**.

## Sample Availability

Samples of the compounds **1**−**13** are available from the authors.
