# Supplementary material for: Benzylated Dihydroflavones and Isoquinoline-Derived Alkaloids from the Bark of Diclinanona calycina (Annonaceae) and Their Cytotoxicities
Source: Molecules. 2021 Jun 18;26(12):3714. doi: 10.3390/molecules26123714 (PMC8235387; doi:10.3390/molecules26123714)
Supplement: Supplementary file 1 [file molecules-26-03714-s001.zip › molecules-1221506-supplementary.pdf]

Supplementary Materials

Benzylated Dihydroflavones and Isoquinoline-Derived Alkaloids from the Bark of *Diclinanona calycina* (Annonaceae) and Their Cytotoxicities

Emmanoel V. Costa [1,*], Liviane do N. Soares [1], Jamal da S. Chaar [1], Valdenizia R. Silva [2], Luciano de S. Santos [2], Hector H. F. Koolen [3], Felipe M. A. da Silva [1], Josean F. Tavares [4], Gokhan Zengin [5], Milena B. P. Soares [2] and Daniel P. Bezerra [2,*]

[1] Department of Chemistry, Federal University of Amazonas (UFAM), Manaus 69080-900, AM, Brazil; evc@ufam.edu.br (E.V.C.); BeM.Liviane@gmail.com (L.d.N.S.); jchaar@ufam.edu.br (J.d.S.C.); felipemourams@gmail.com (F.M.A.d.S.)

[2] Gonçalo Moniz Institute, Oswaldo Cruz Foundation (IGM-FIOCRUZ/BA), Salvador 40296-710, BA, Brazil; valdeniziar@gmail.com (V.R.S.); luciano.biomed@gmail.com (L.d.S.S.); milenabpsoares@gmail.com (M.B.P.S.); daniel.bezerra@fiocruz.br (D.P.B.)

[3] Metabolomics and Mass Spectrometry Research Group, Amazonas State University (UEA), Manaus 690065-130, AM, Brazil; hkoolen@uea.edu.br

[4] Health Sciences Center, Postgraduate Program in Natural and Synthetic Bioactive Products, Federal University of Paraíba (UFPB), João Pessoa 58051-970, PA, Brazil; josean@ltf.ufpb.br

[5] Department of Biology, Science Faculty, Selcuk University, Konya 42130, Turkey; gokhanzengin@selcuk.edu.tr

* Correspondence: evc@ufam.edu.br (E.V.C.); daniel.bezerra@fiocruz.br (D.P.B.); Tel./Fax: +55-92-3305-1181 Ramal 2870 (E.V.C.); +55-71-3176-2272 (D.P.B.)

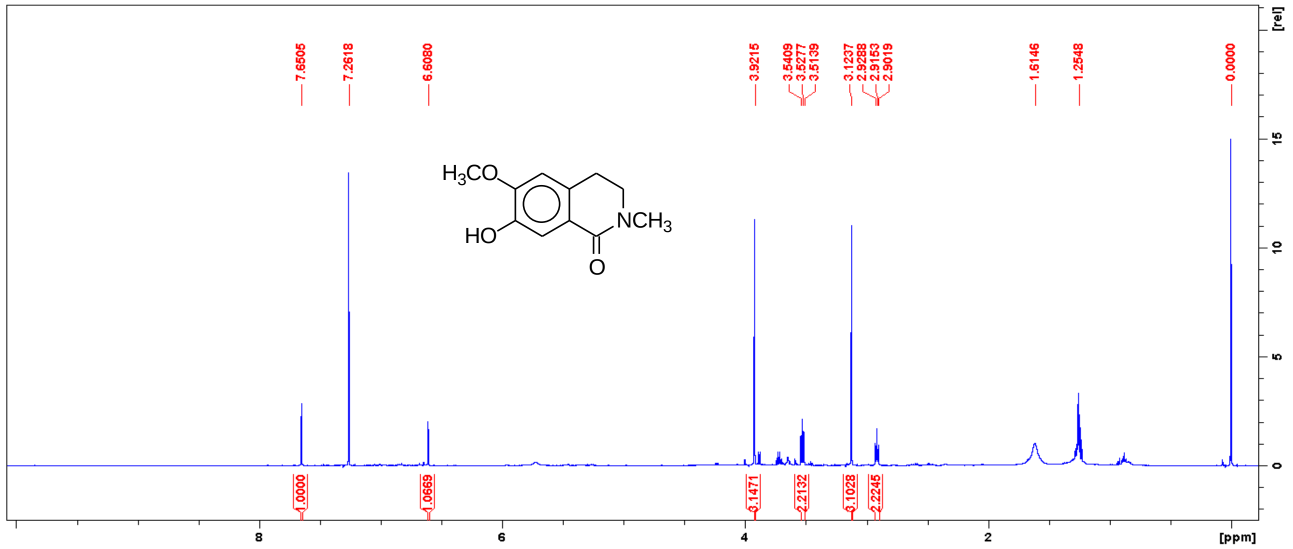

Figure S1. *^{1}H NMR spectrum of **thalifoline (1)** in $CDCl_3$ at 500 MHz.*

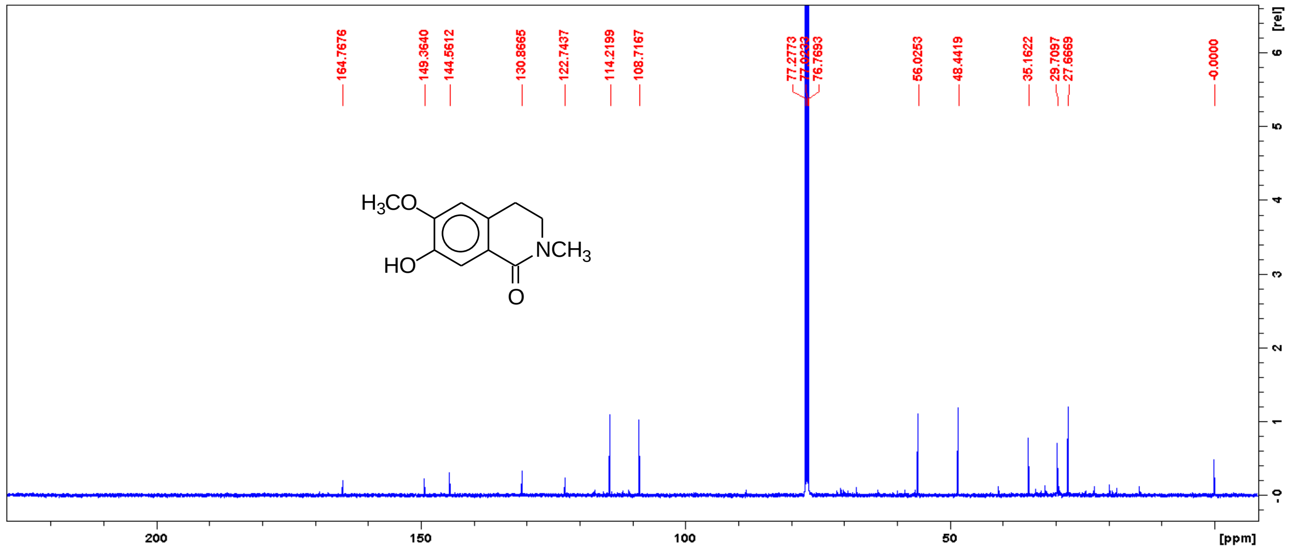

Figure S2. *^{13}C NMR spectrum of **thalifoline (1)** in $CDCl_3$ at 100 MHz.*

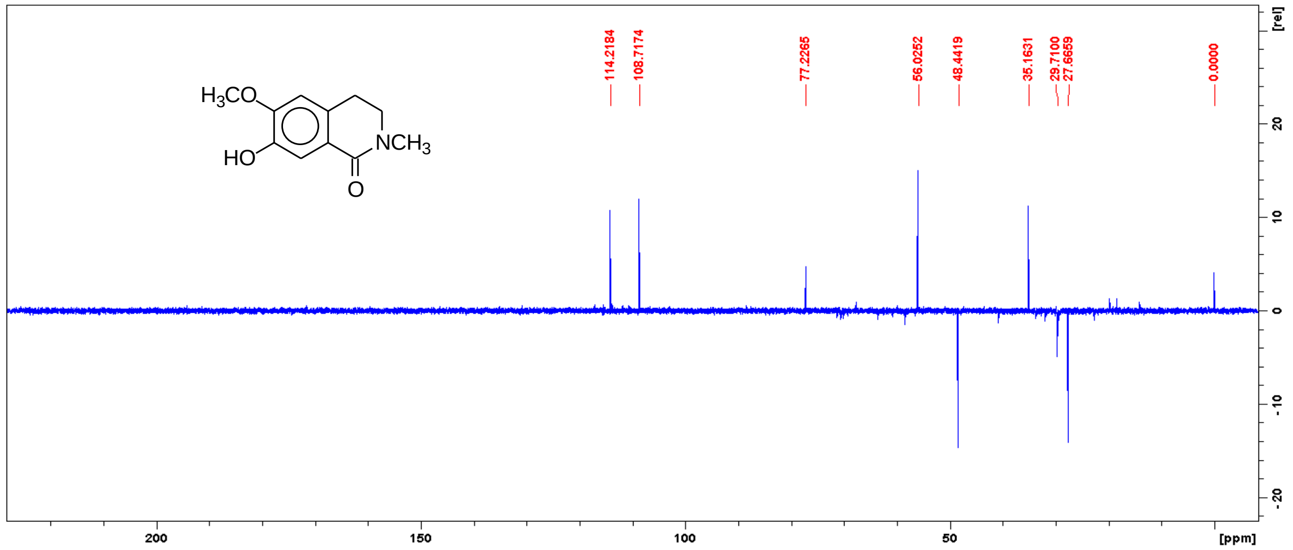

Figure S3. *DEPT 135 spectrum of **thalifoline (1)** in $CDCl_3$ at 125 MHz.*

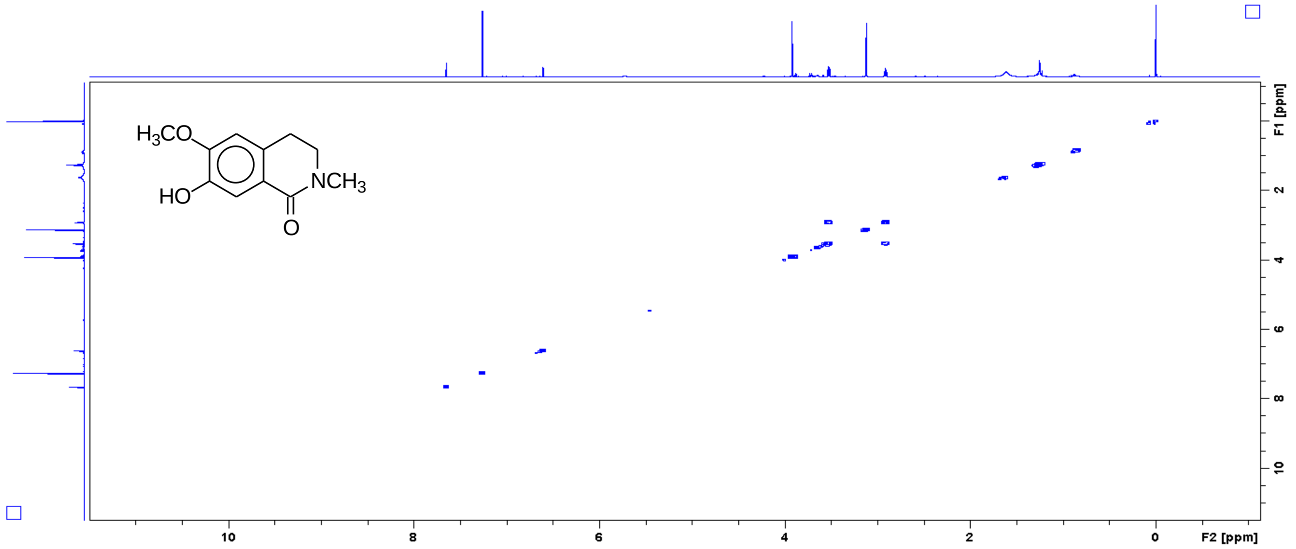

Figure S4. *^{1}H-^{1}H correlation map from COSY NMR experiment of **thalifoline** (**1**) in $CDCl_3$ at 500 MHz.*

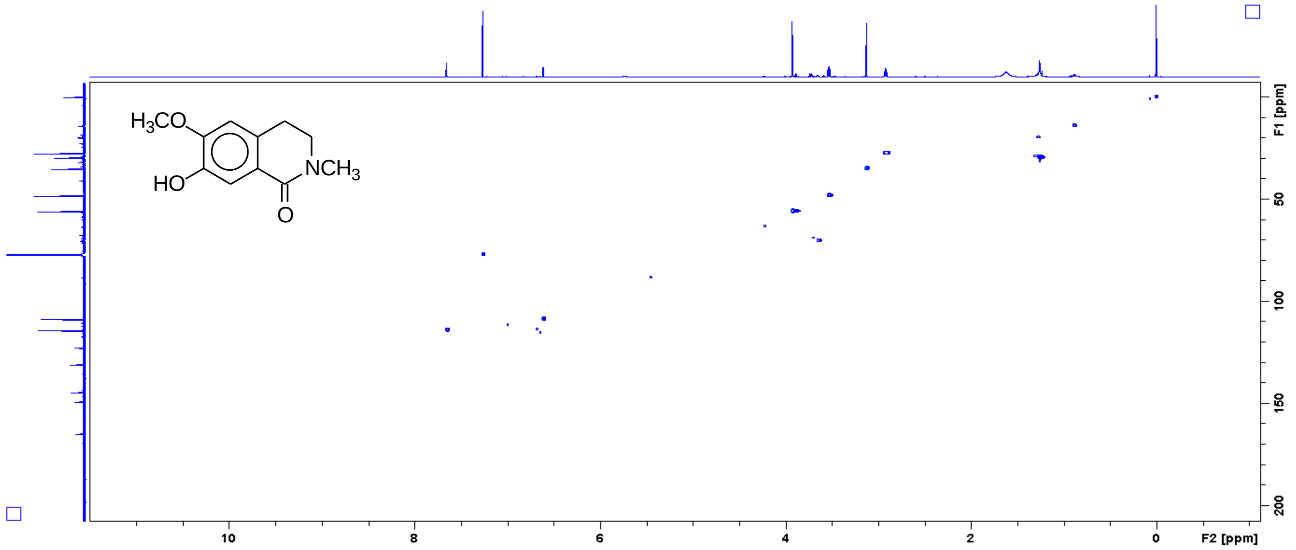

Figure S5. *One-bond ^{1}H-^{13}C correlation map from HSQC NMR experiment of **thalifoline** (**1**) in $CDCl_3$ at 500 (^{1}H) and 125 MHz (^{13}C), respectively.*

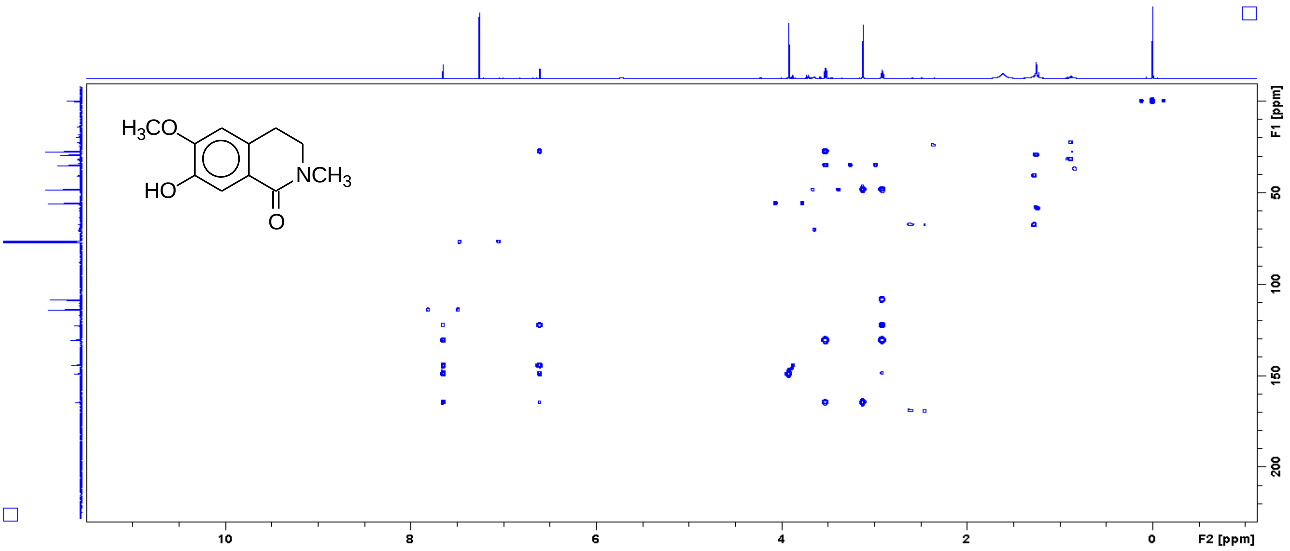

Figure S6. *Long-range ^{1}H-^{13}C correlation map from HMBC NMR experiment of* ***thalifoline (1)*** *in $CDCl_3$ at 500 (^{1}H) and 125 MHz (^{13}C), respectively.*

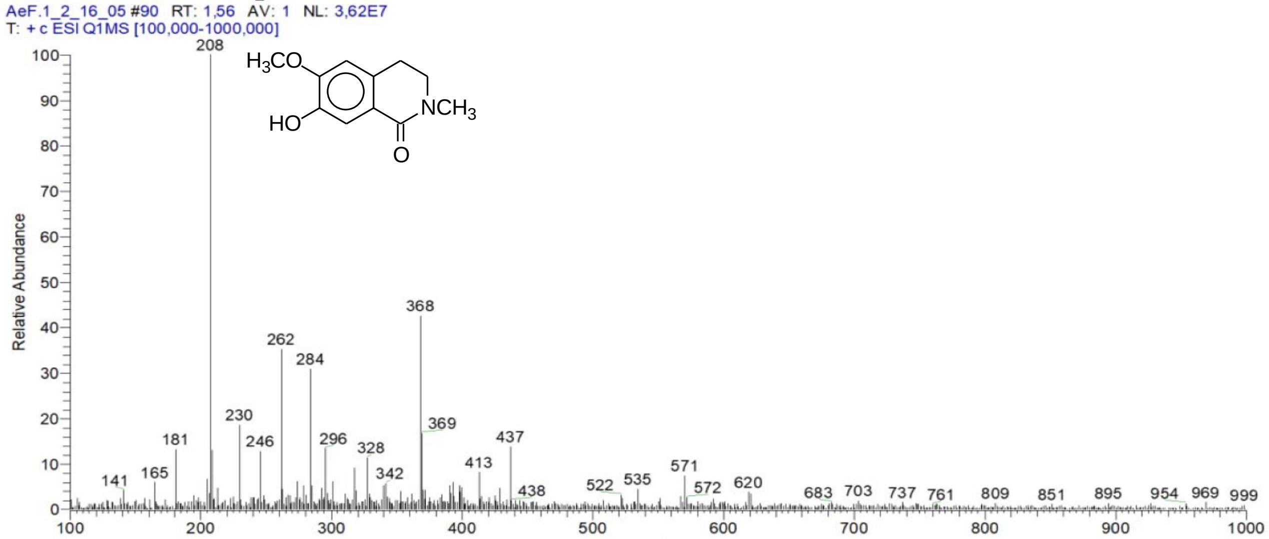

Figure S7. *LR-ESI(+)-MS spectrum of* ***thalifoline (1)*** *(m/z 208 $[M+H]^+$).*

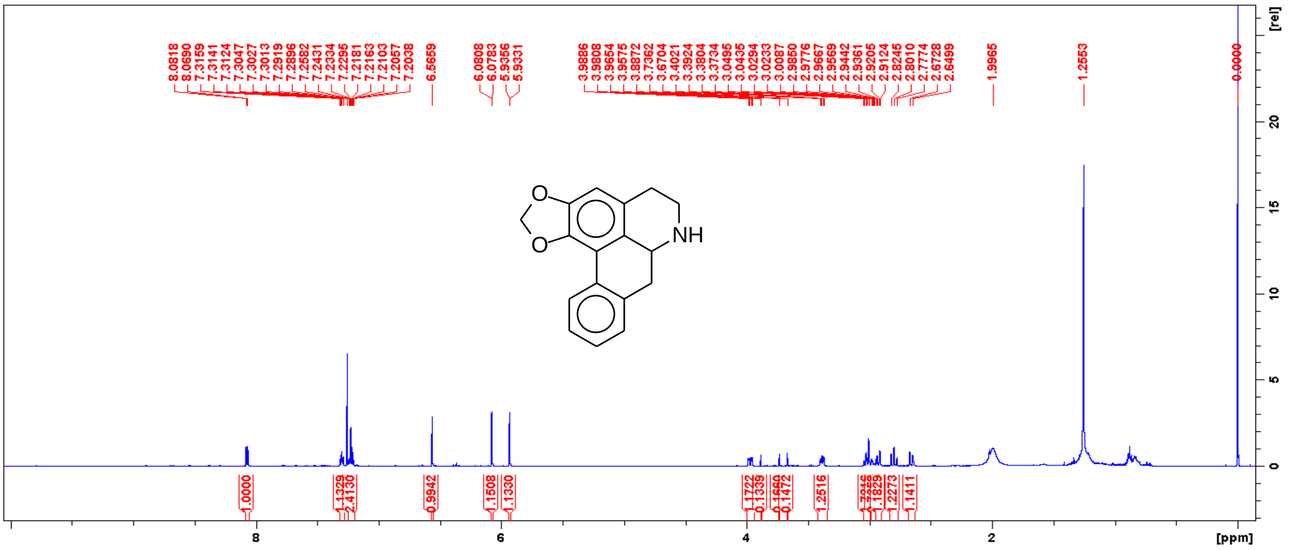

Figure S8. *^{1}H NMR spectrum of* ***anonaine (2)*** *in $CDCl_3$ at 600 MHz.*

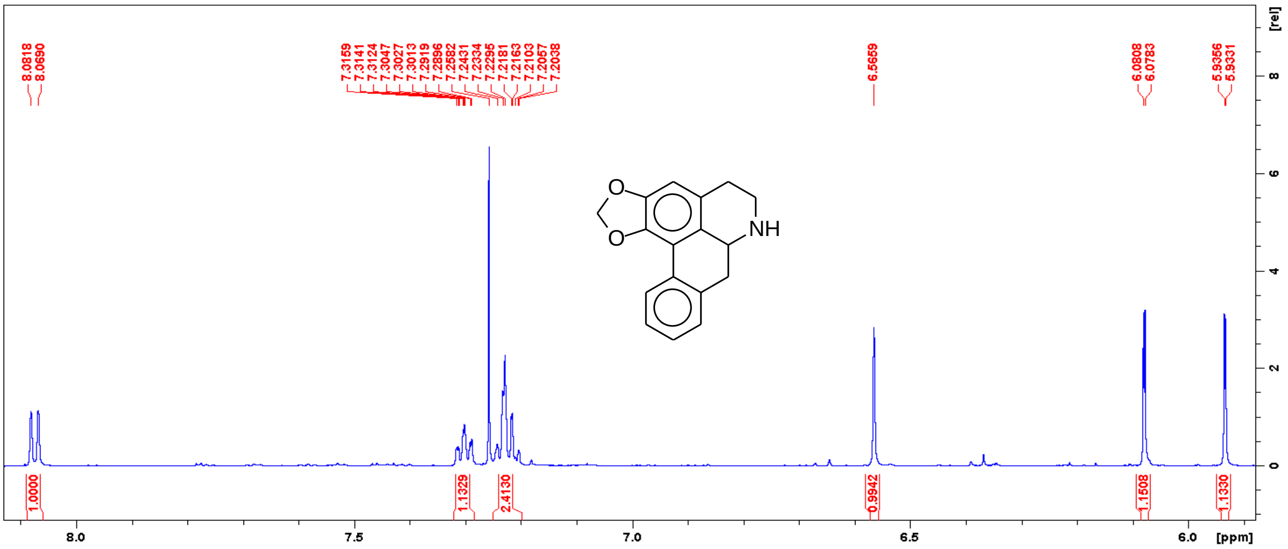

Figure S9. *Expansion (δ 8.12 – 5.88) of the* [1]*H NMR spectrum of* ***anonaine (2)*** *in CDCl₃ at 600 MHz.*

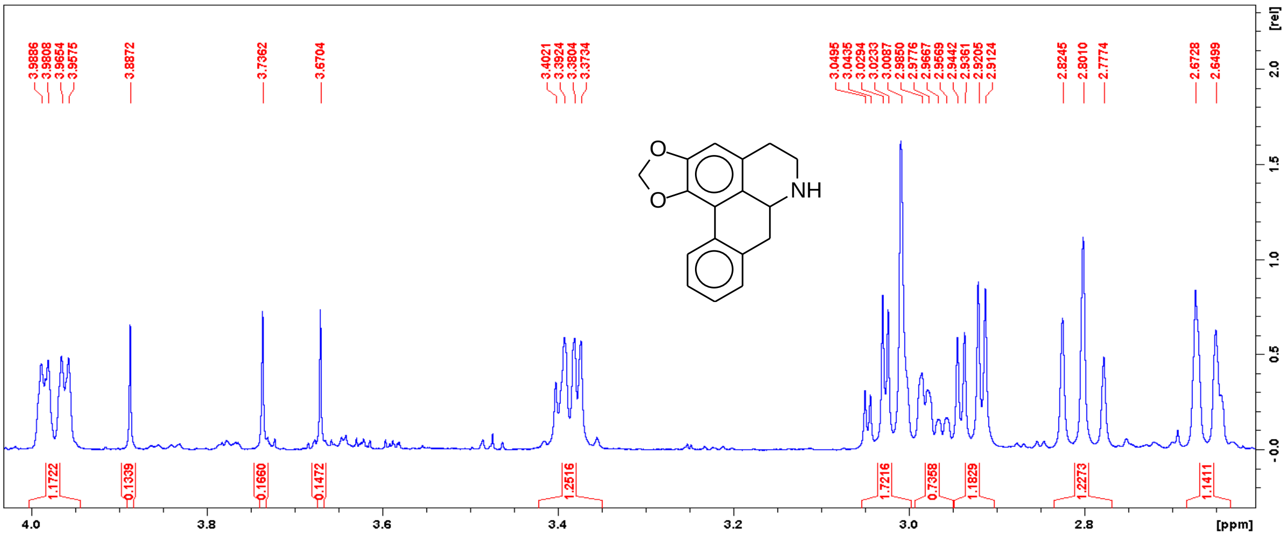

Figure S10. *Expansion (δ 4.02 – 2.61) of the* [1]*H NMR spectrum of* ***anonaine (2)*** *in CDCl₃ at 600 MHz.*

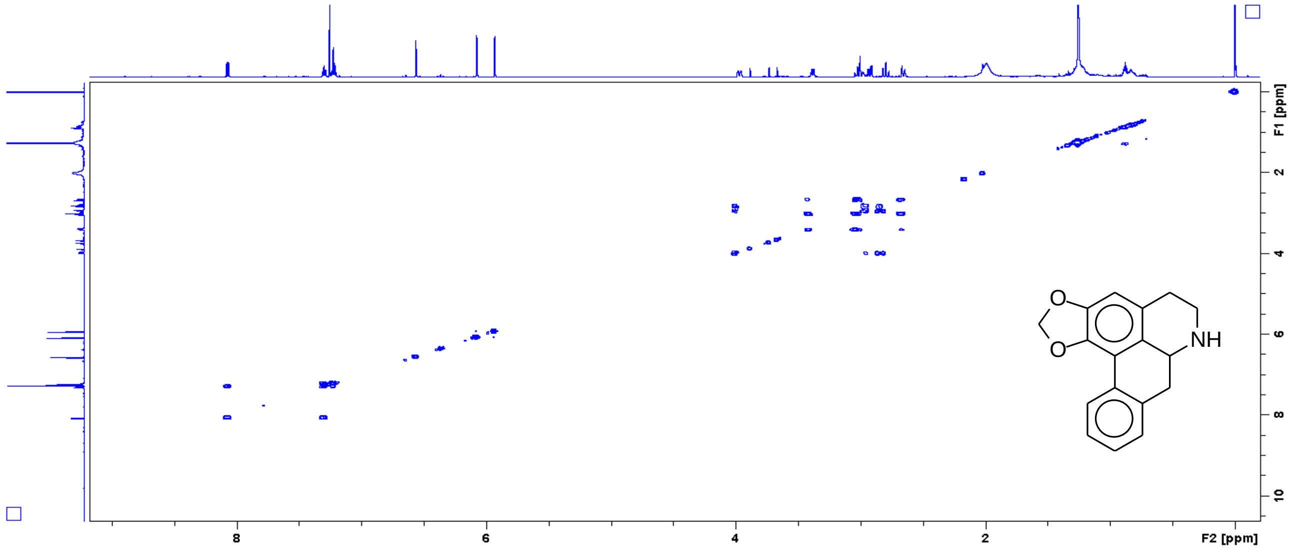

Figure S11. [1]*H-*[1]*H correlation map from COSY NMR experiment of* ***anonaine (2)*** *in CDCl₃ at 600 MHz.*

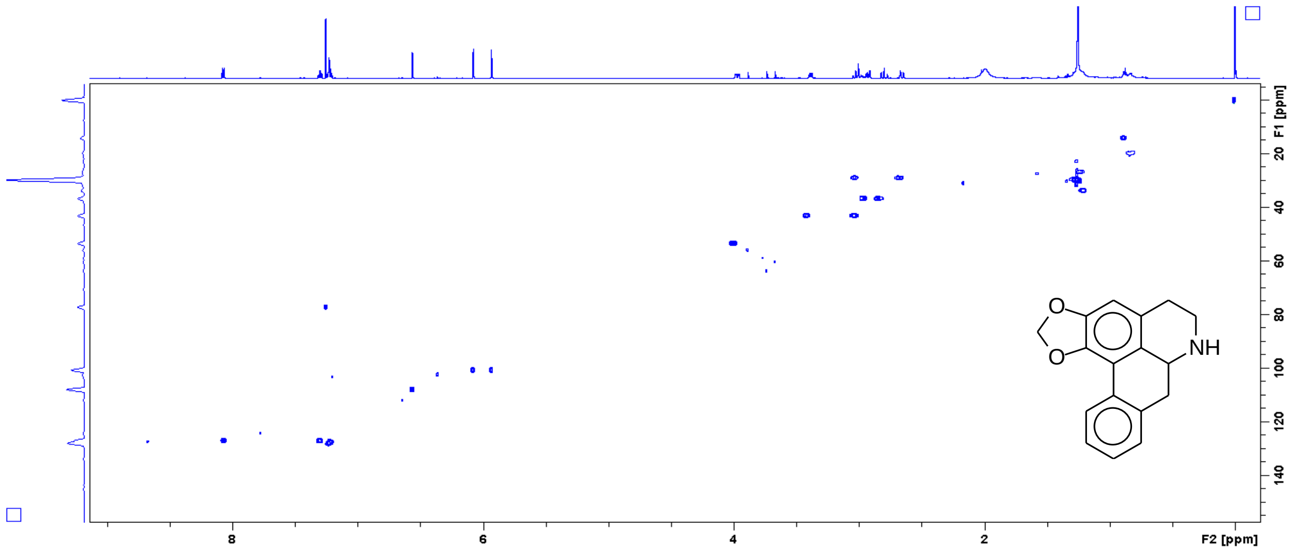

Figure S12. *One-bond* ^{1}H-^{13}C *correlation map from HSQC NMR experiment of* ***anonaine*** *(**2**) in* $CDCl_3$ *at 600 (*^{1}H*) and 150 MHz (*^{13}C*), respectively.*

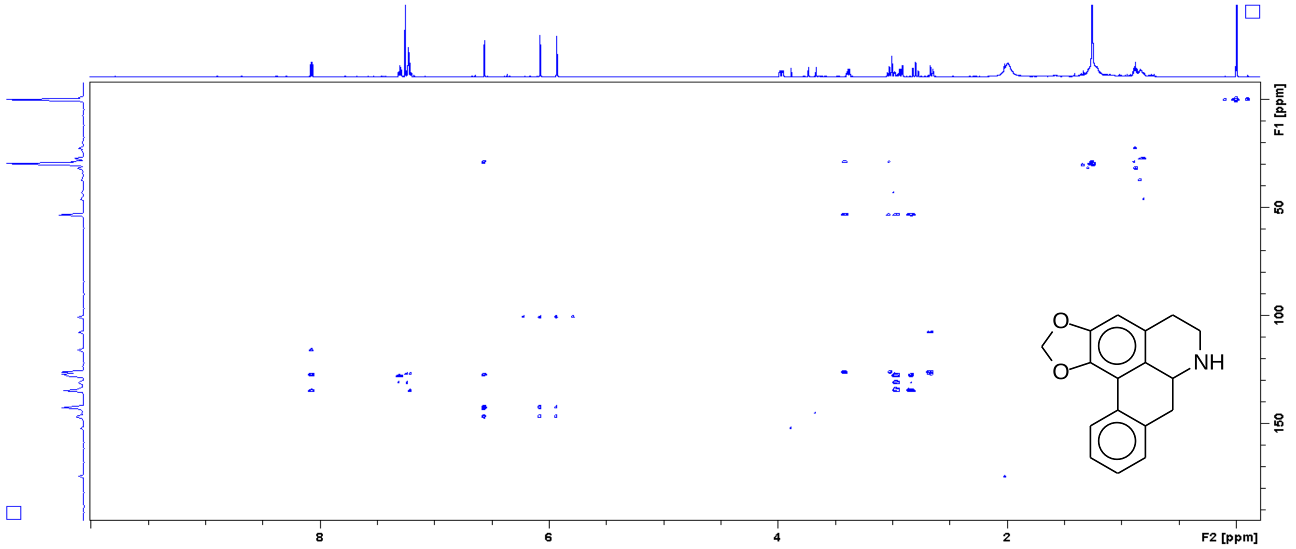

Figure S13. *Long-range* ^{1}H-^{13}C *correlation map from HMBC NMR experiment of* ***anonaine*** *(**2**) in* $CDCl_3$ *at 600 (*^{1}H*) and 150 MHz (*^{13}C*), respectively.*

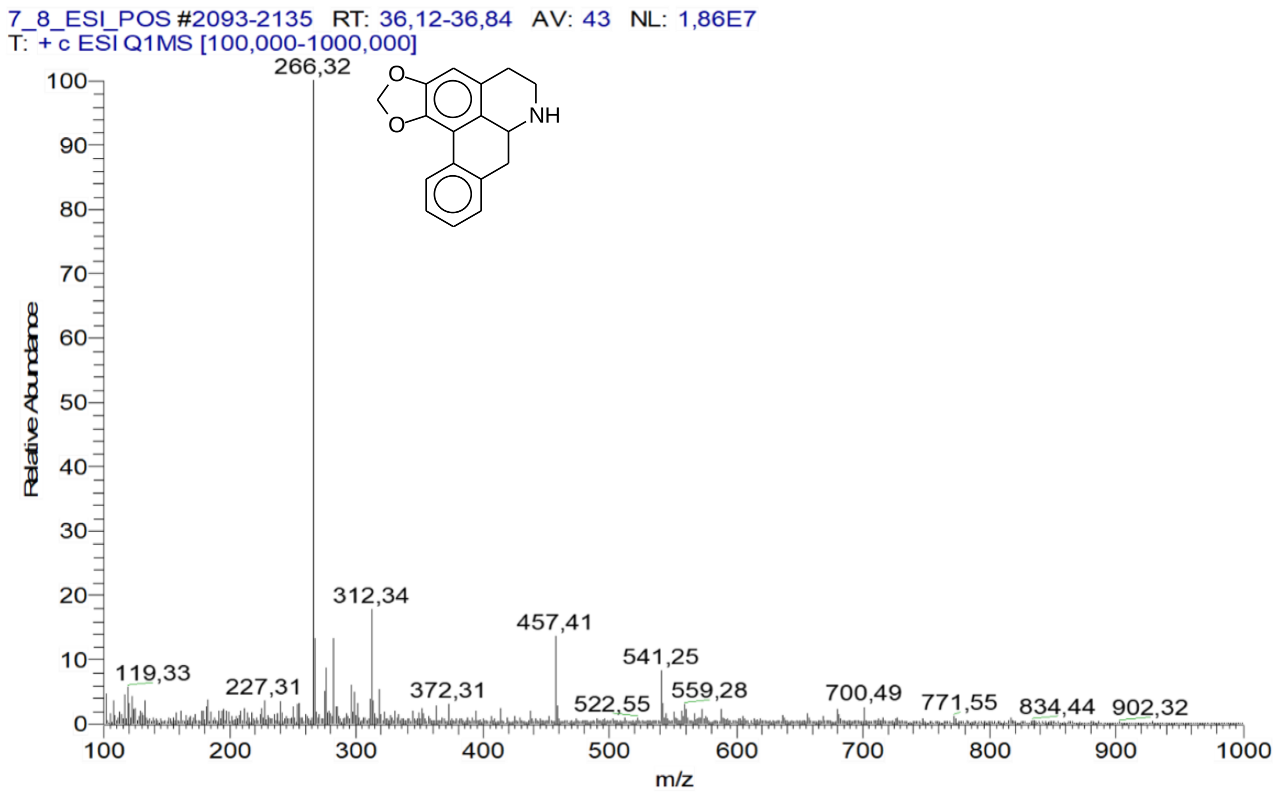

Figure S14. *LR-ESI(+)-MS spectrum of* ***anonaine*** *(****2****) (m/z 266 [M+H]$^+$).*

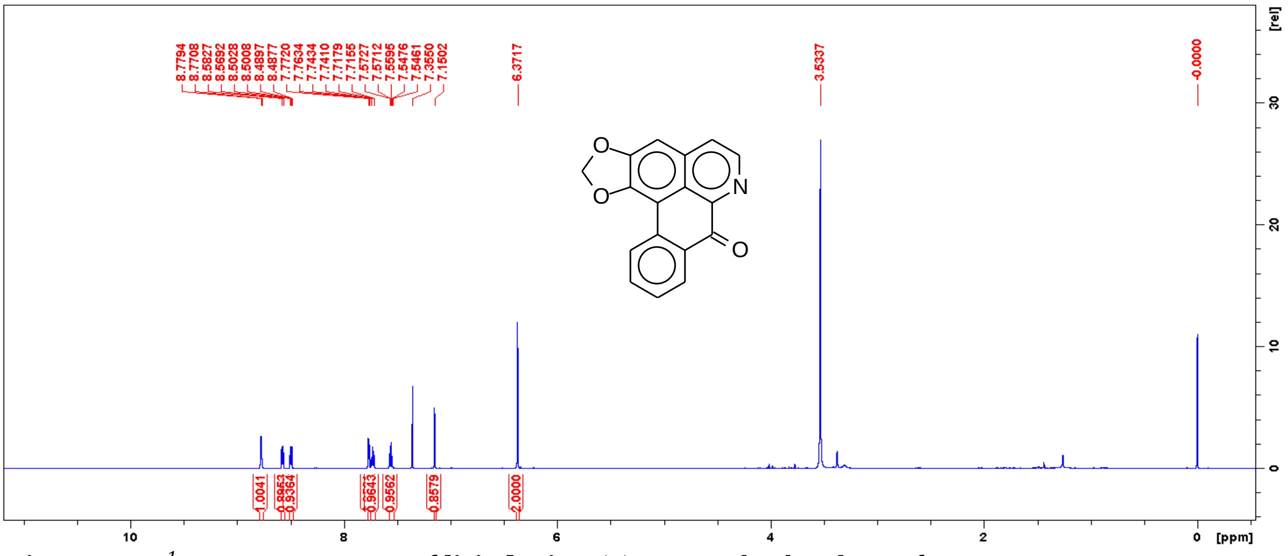

Figure S15. *^{1}H NMR spectrum of* ***liriodenine*** *(****3****) in $CDCl_3$ plus drop of CD_3OD at 600 MHz.*

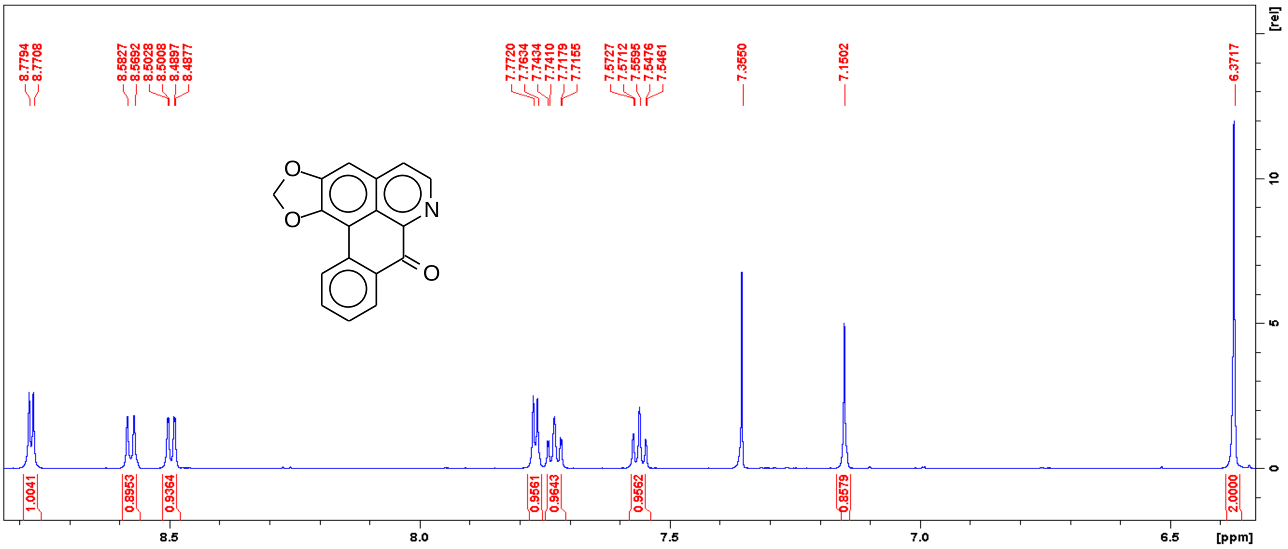

Figure S16. *Expansion (δ 8.82 – 6.33) of the* [1]*H NMR spectrum of* ***liriodenine (3)*** *in* $CDCl_3$ *plus drop of* CD_3OD *at 600 MHz.*

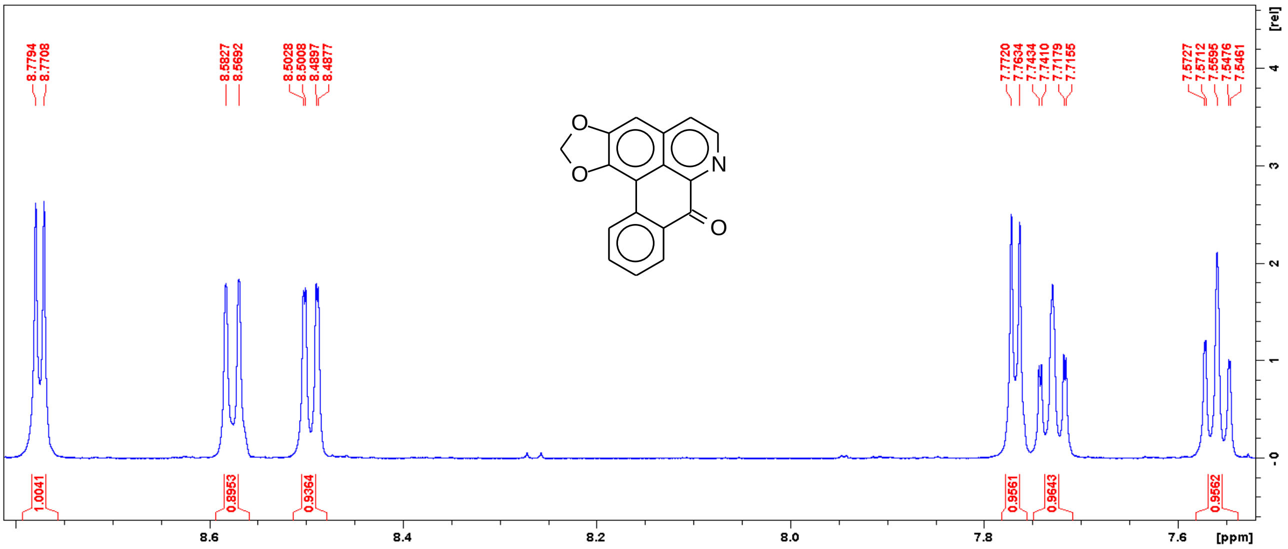

Figure S17. *Expansion (δ 8.81 – 7.52) of the* [1]*H NMR spectrum of* ***liriodenine (3)*** *in* $CDCl_3$ *plus drop of* CD_3OD *at 600 MHz.*

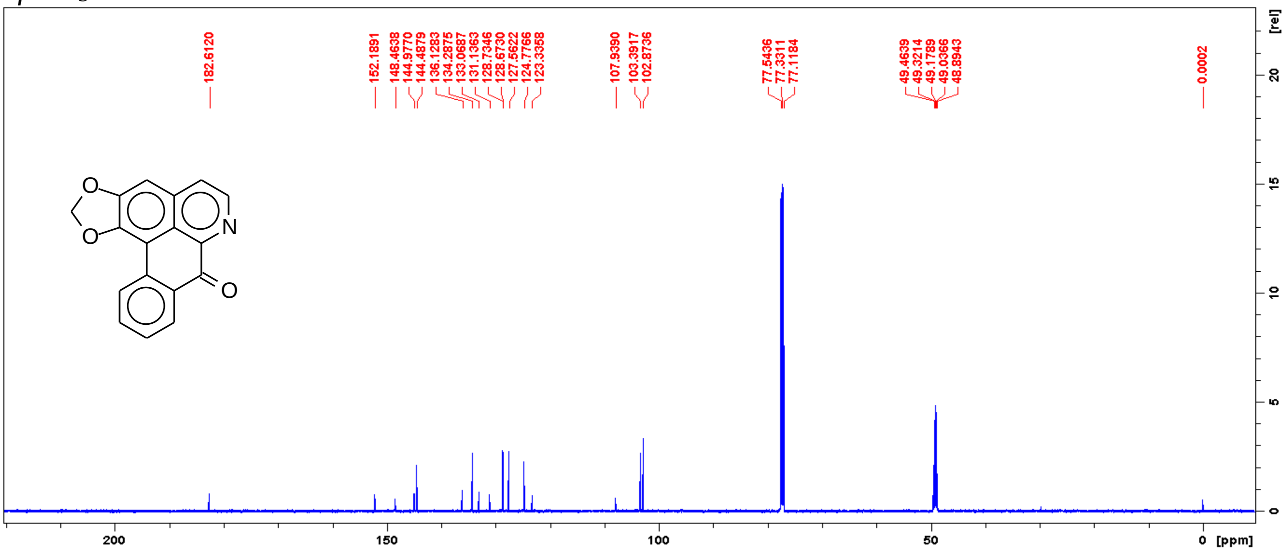

Figure S18. [13]*C NMR spectrum of* ***liriodenine (3)*** *in* $CDCl_3$ *plus drop of* CD_3OD *at 150 MHz.*

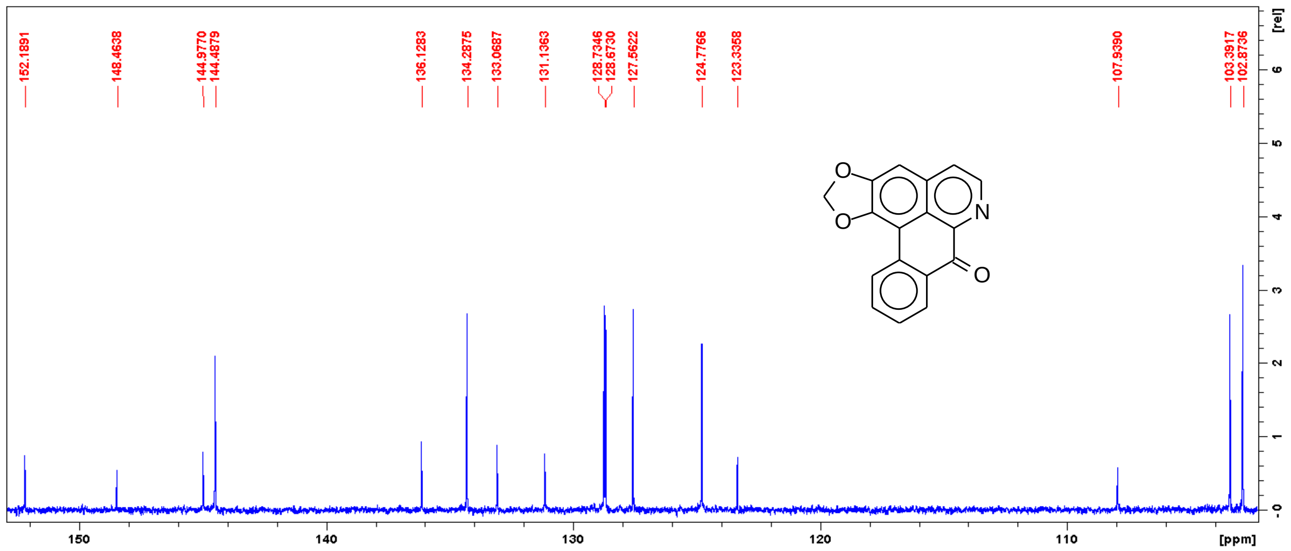

Figure S19. *Expansion (δ 152.1 – 102.4) of the* ^{13}*C NMR spectrum of* ***liriodenine (3)*** *in* $CDCl_3$ *plus drop of* CD_3OD *at 150 MHz.*

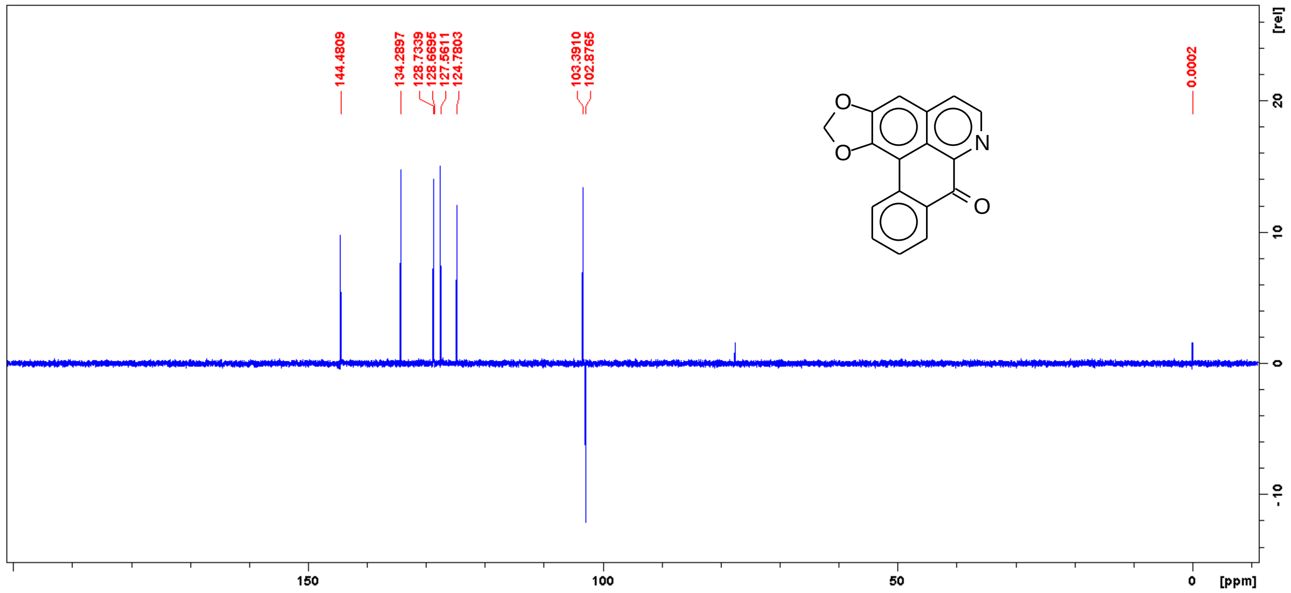

Figure S20. *DEPT 135 spectrum of* ***liriodenine (3)*** *in* $CDCl_3$ *plus drop of* CD_3OD *at 150 MHz.*

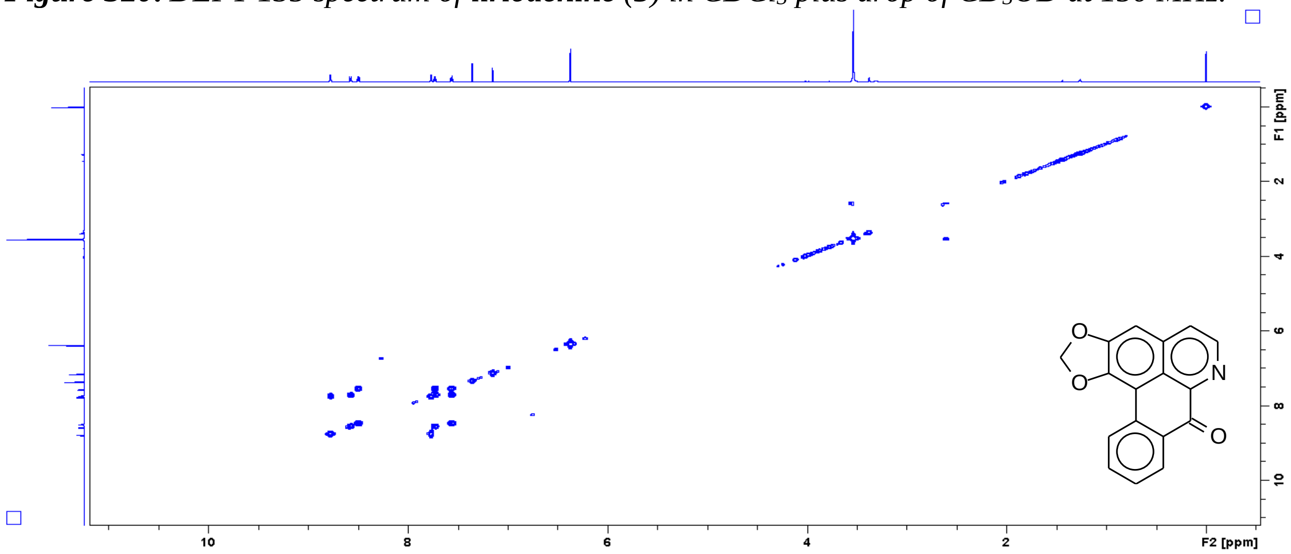

Figure S21. ^{1}*H-*^{1}*H correlation map from COSY NMR experiment of* ***liriodenine (3)*** *in* $CDCl_3$ *plus drop of* CD_3OD *at 600 MHz.*

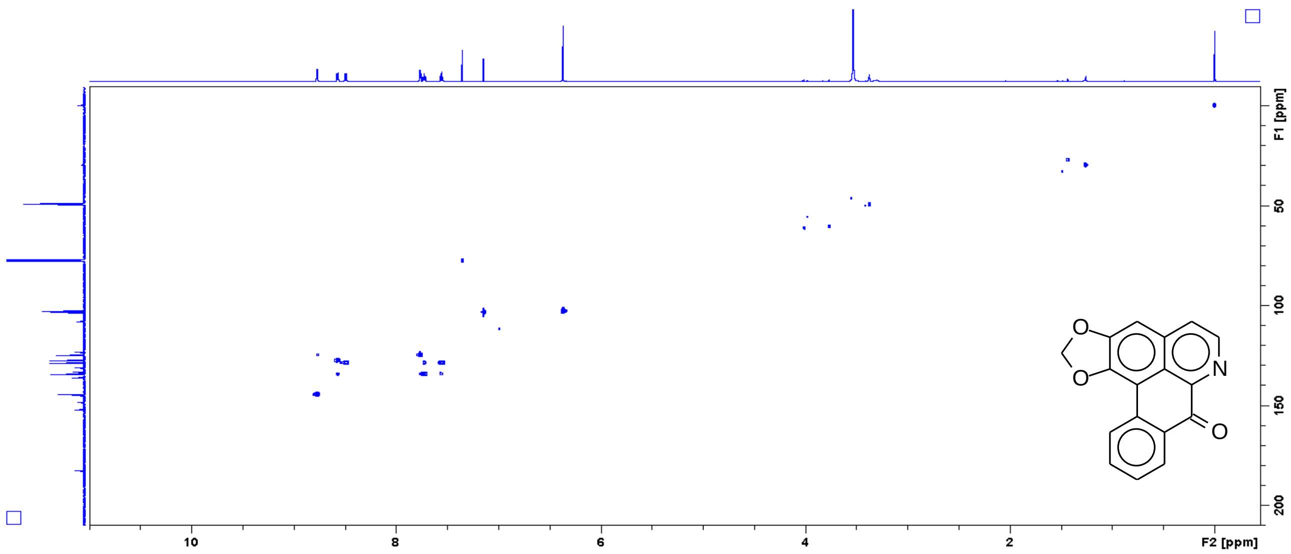

Figure S22. *One-bond ^{1}H-^{13}C correlation map from HSQC NMR experiment of* ***liriodenine (3)*** *in $CDCl_3$ plus drop of CD_3OD at 600 (^{1}H) and 150 MHz (^{13}C), respectively.*

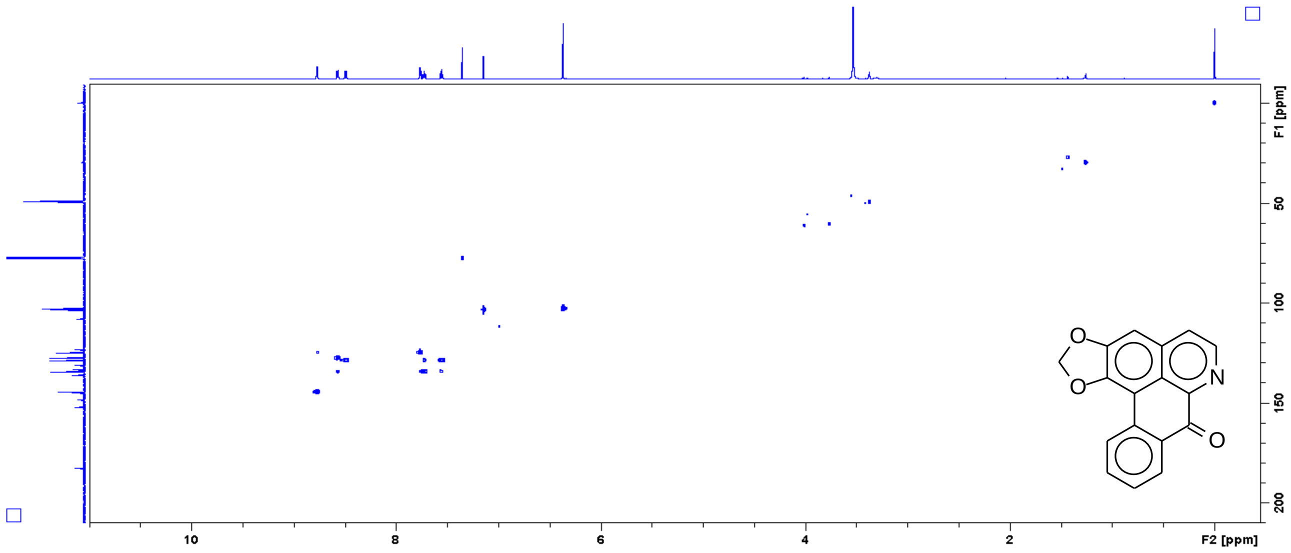

Figure S23. *Long-range ^{1}H-^{13}C correlation map from HMBC NMR experiment of* ***liriodenine (3)*** *in $CDCl_3$ plus drop of CD_3OD at 600 (^{1}H) and 150 MHz (^{13}C), respectively.*

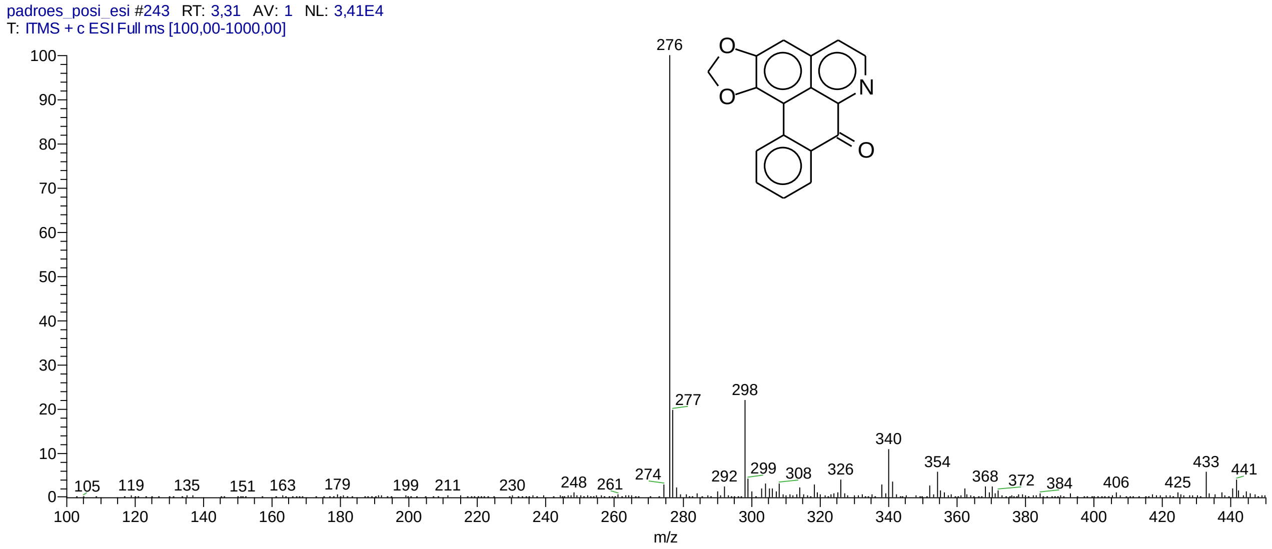

Figure S24. *LR-ESI(+)-MS spectrum of* ***liriodenine*** *(**3**) (m/z 276 $[M+H]^{+}$).*

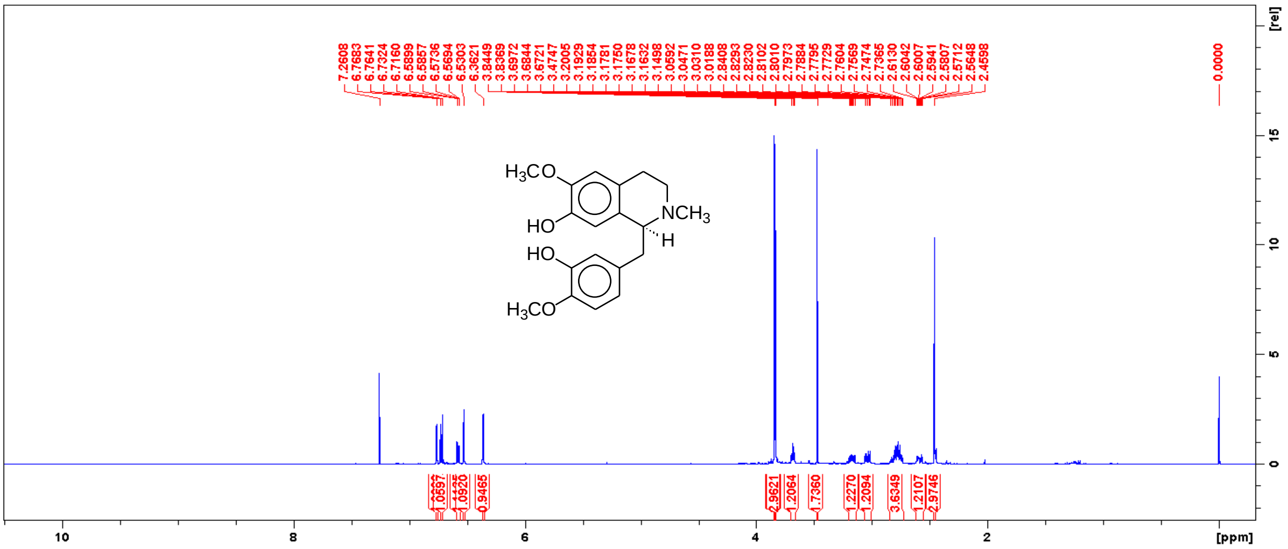

Figure S25. *^{1}H NMR spectrum of* ***reticuline (4)*** *in $CDCl_3$ at 500 MHz.*

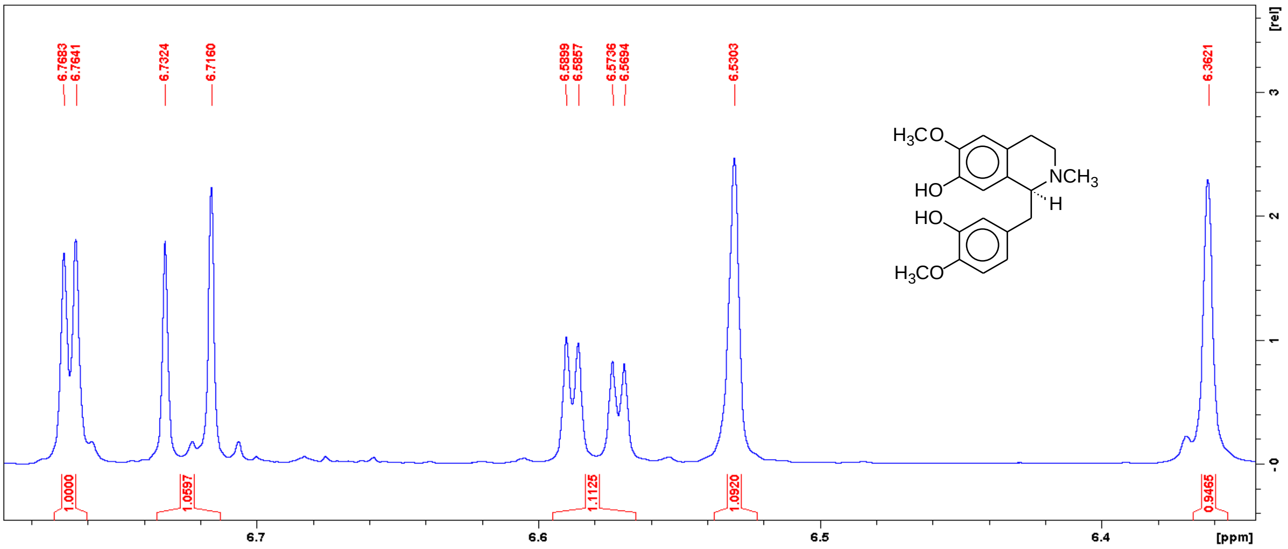

Figure S26. *Expansion (δ 6.78 – 6.34) of the ^{1}H NMR spectrum of* ***reticuline (4)*** *in $CDCl_3$ at 500 MHz.*

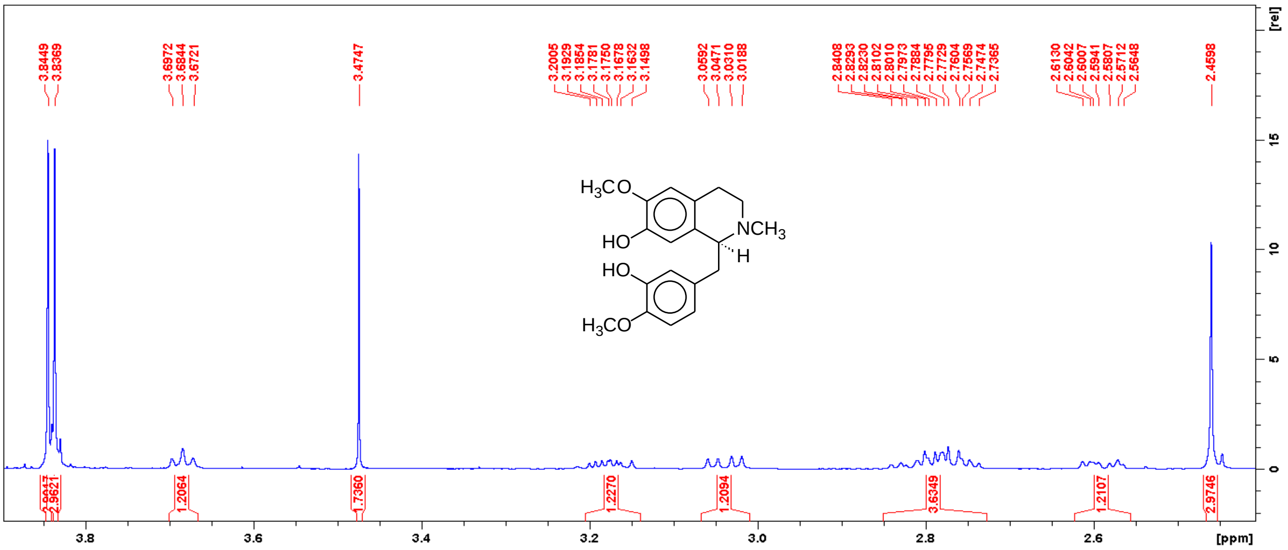

Figure S27. *Expansion (δ 3.88 – 2.41) of the ^{1}H NMR spectrum of* ***reticuline (4)*** *in $CDCl_3$ at 500 MHz.*

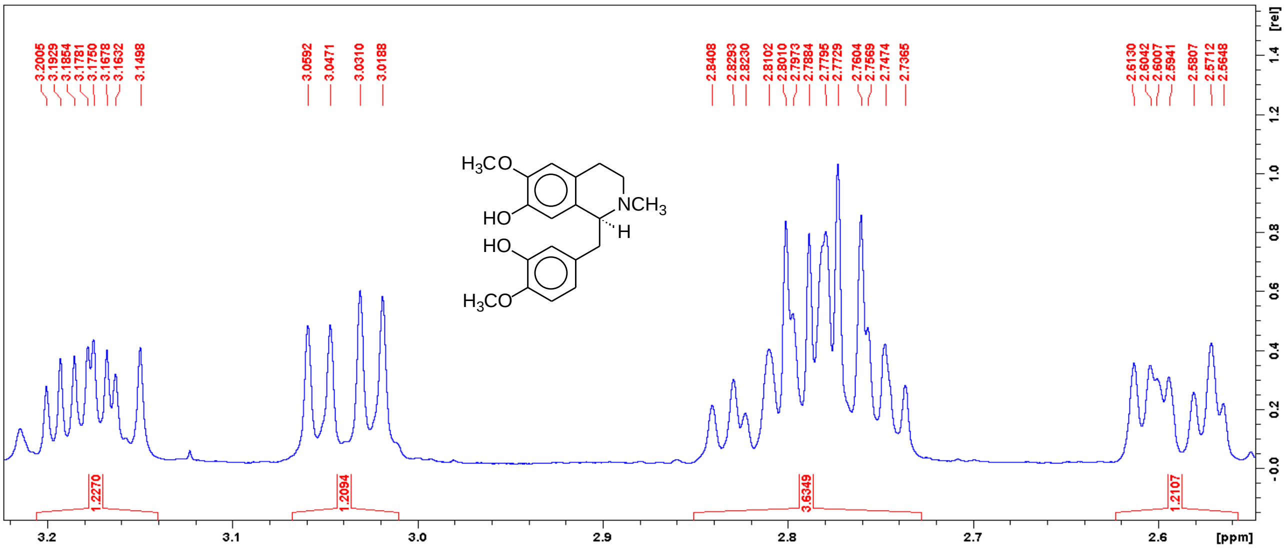

Figure S28. *Expansion (δ 3.22 – 2.55) of the ^{1}H NMR spectrum of* ***reticuline (4)*** *in $CDCl_3$ at 500 MHz.*

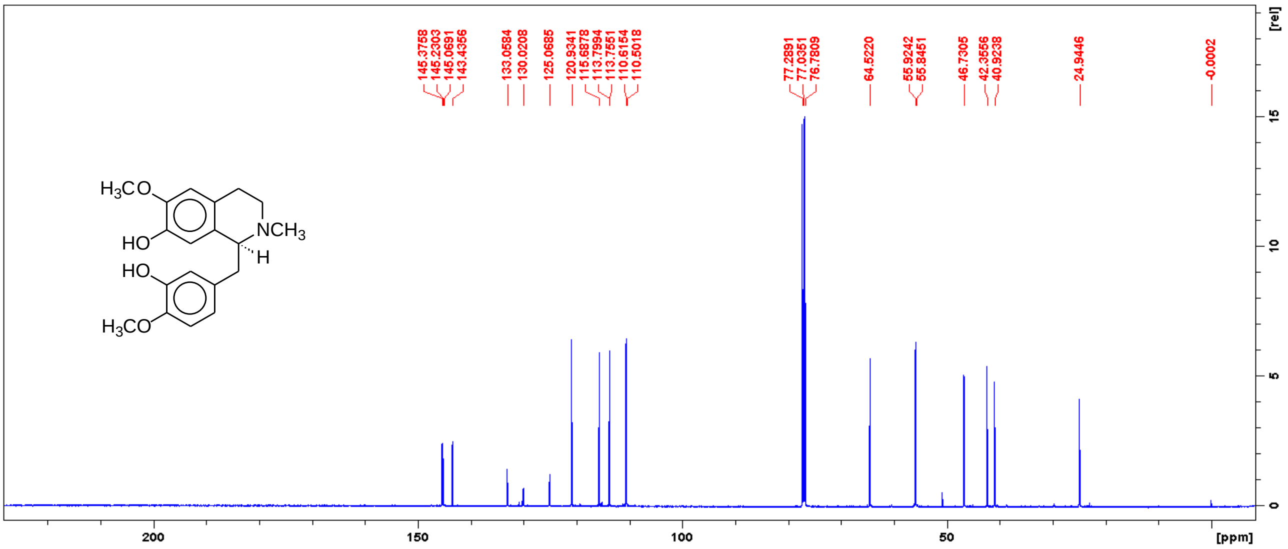

Figure S29. *^{13}C NMR spectrum of* ***reticuline (4)*** *in $CDCl_3$ at 125 MHz.*

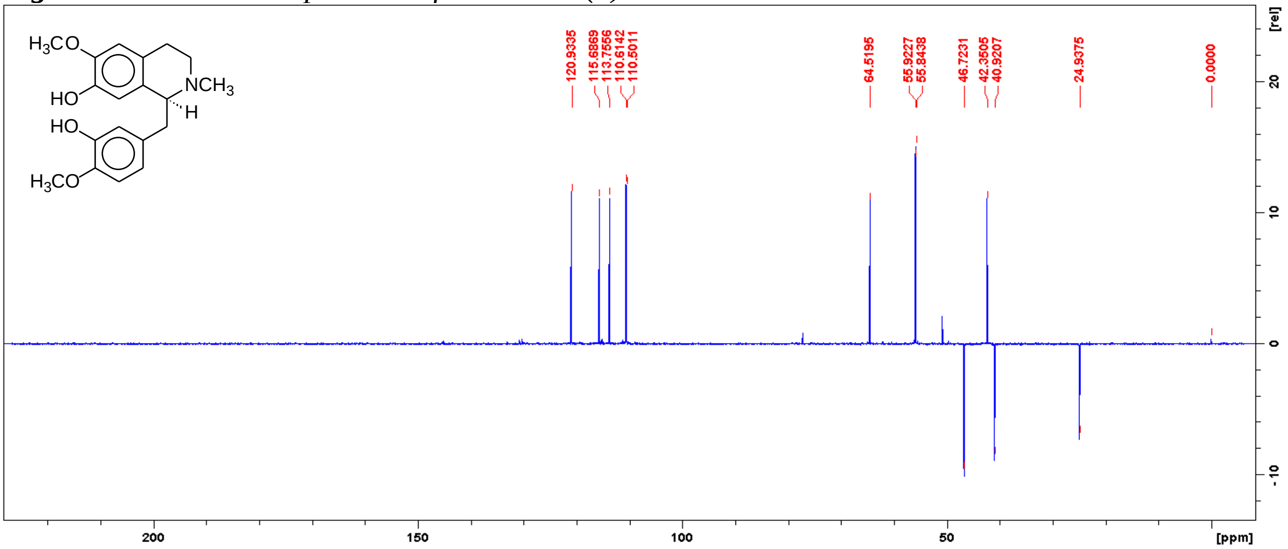

Figure S30. *DEPT 135 spectrum of* ***reticuline (4)*** *in $CDCl_3$ at 125 MHz.*

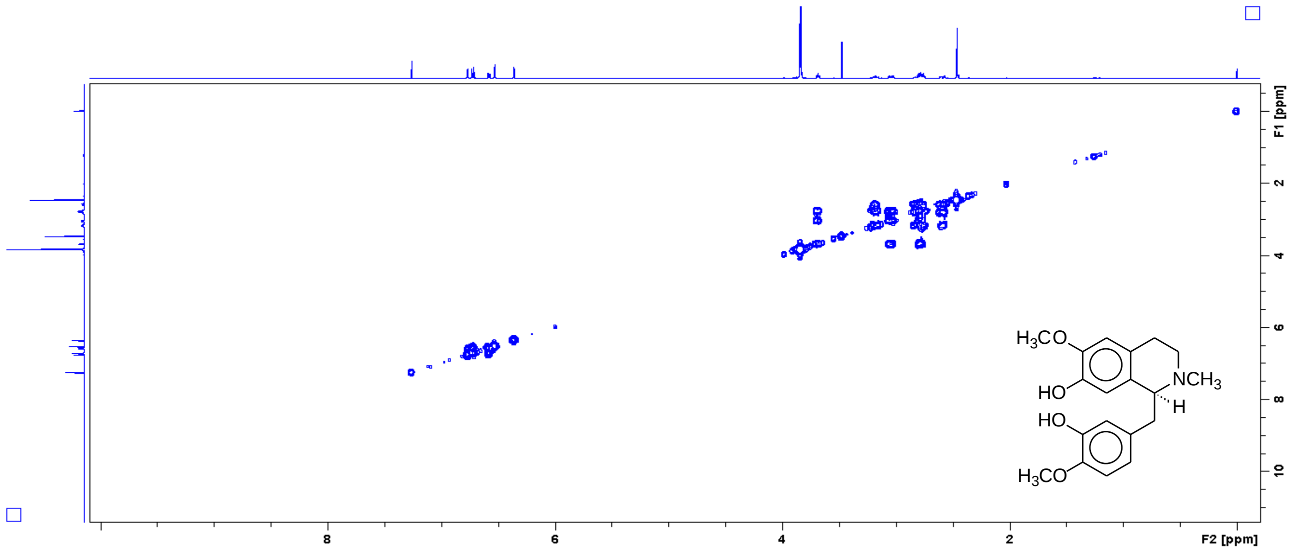

Figure S31. *^1H-^1H correlation map from COSY NMR experiment of* ***reticuline (4)*** *in $CDCl_3$ at 500 MHz.*

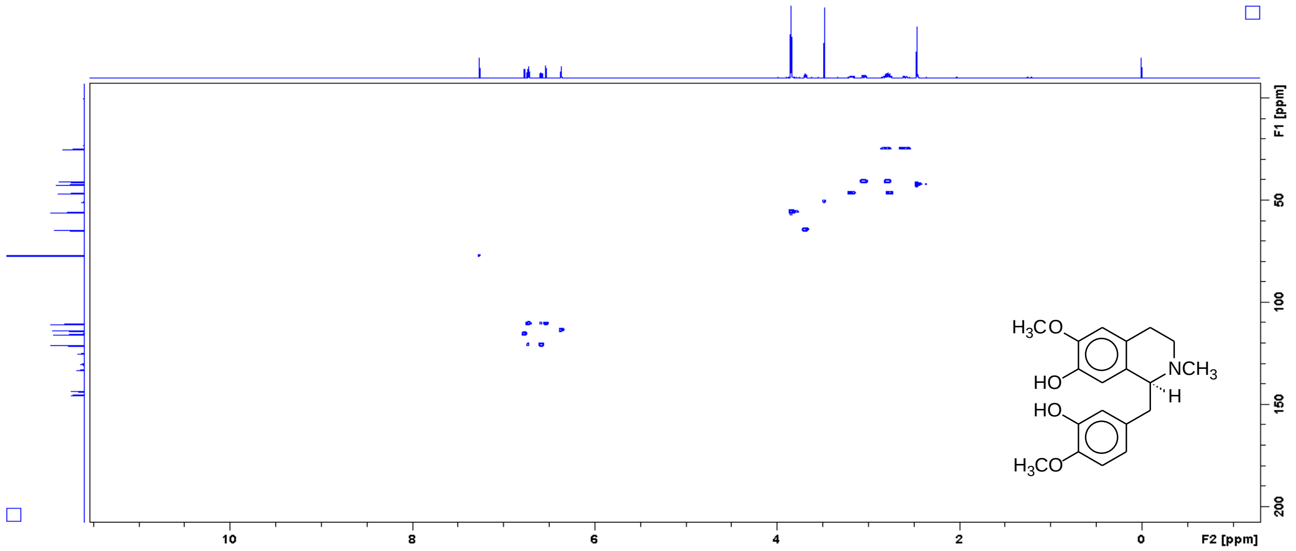

Figure S32. *One-bond ^1H-^{13}C correlation map from HSQC NMR experiment of* ***reticuline (4)*** *in $CDCl_3$ at 500 (^1H) and 125 MHz (^{13}C), respectively.*

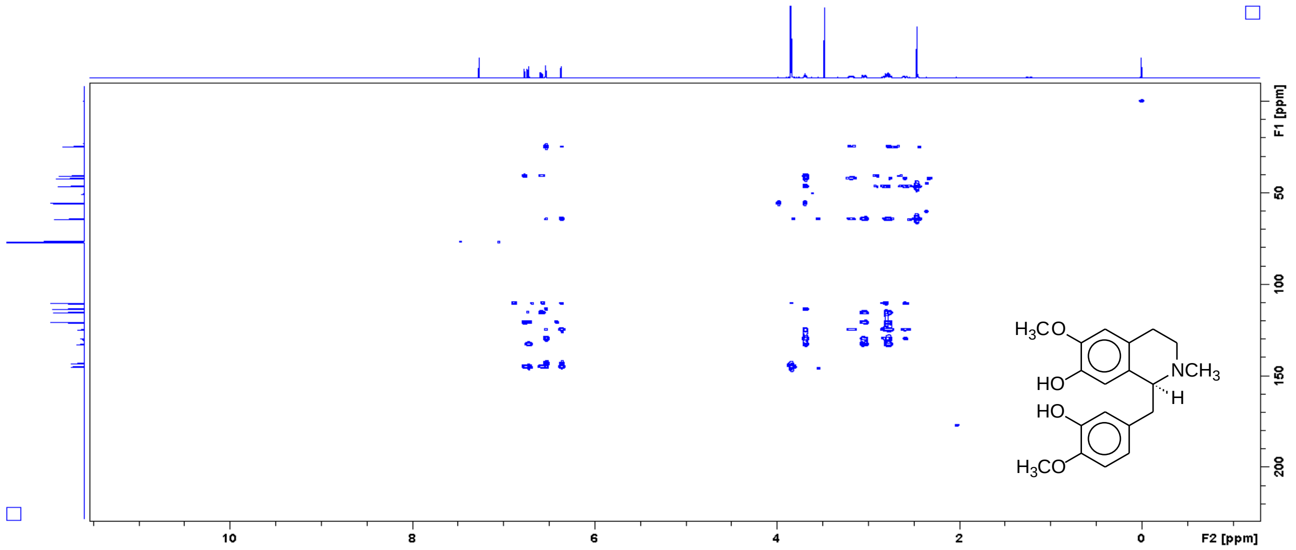

Figure S33. *Long-range ^1H-^{13}C correlation map from HMBC NMR experiment of* ***reticuline (4)*** *in $CDCl_3$ at 500 (^1H) and 125 MHz (^{13}C), respectively.*

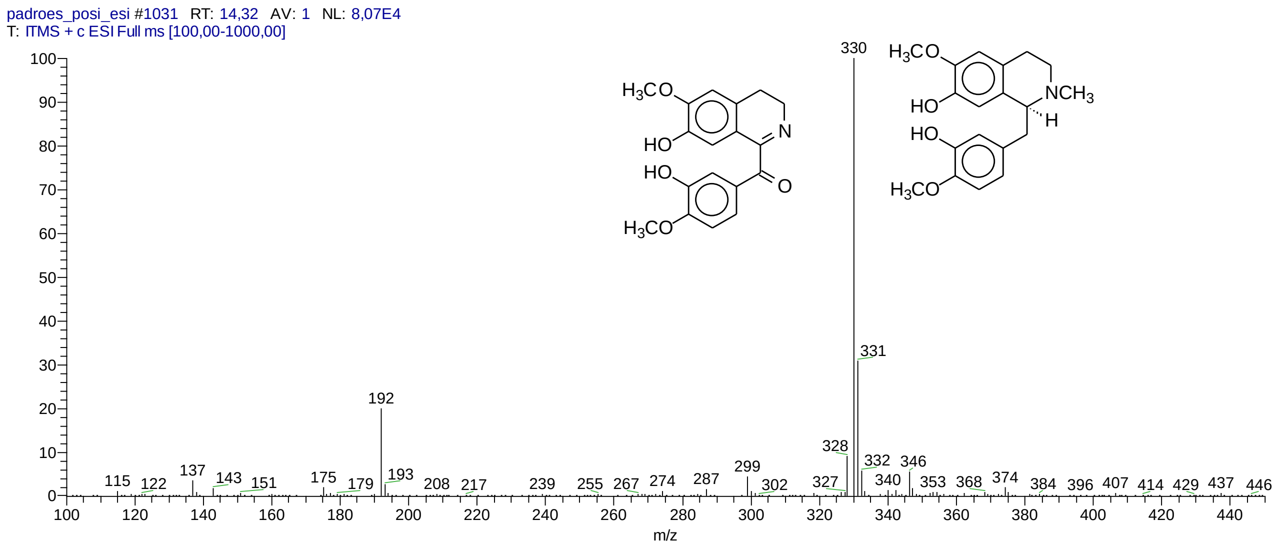

Figure S34. *LR-ESI(+)-MS spectrum of* ***reticuline*** *(4) (m/z 330 [M+H]$^+$).*

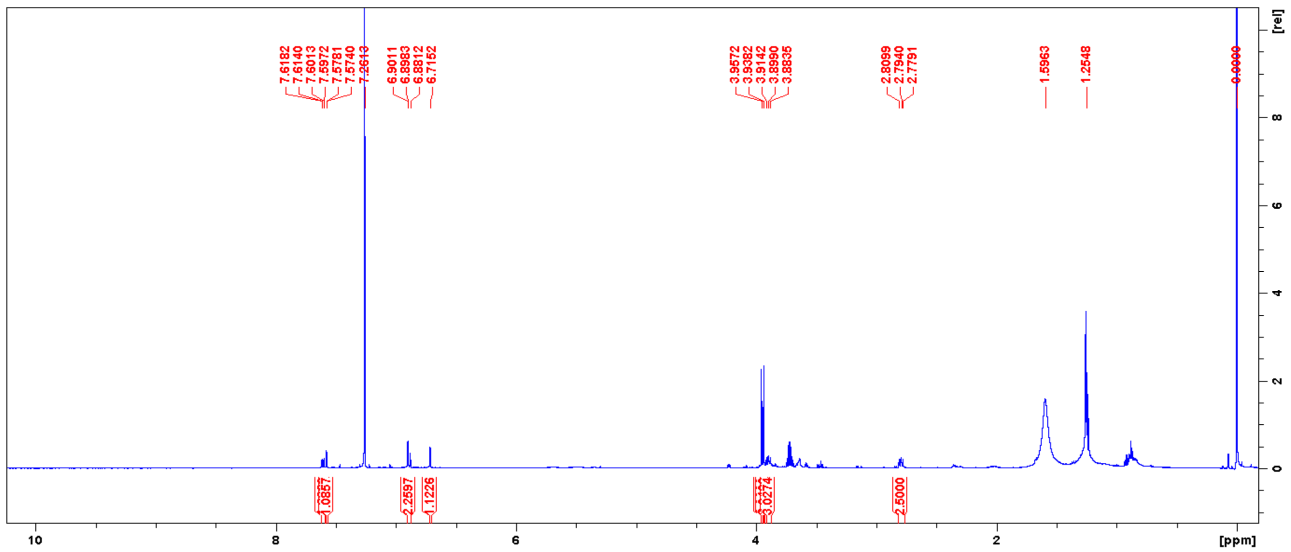

Figure S35. *^{1}H NMR spectrum of* ***dehydro-oxonorreticuline*** *(5) in $CDCl_3$ at 500 MHz.*

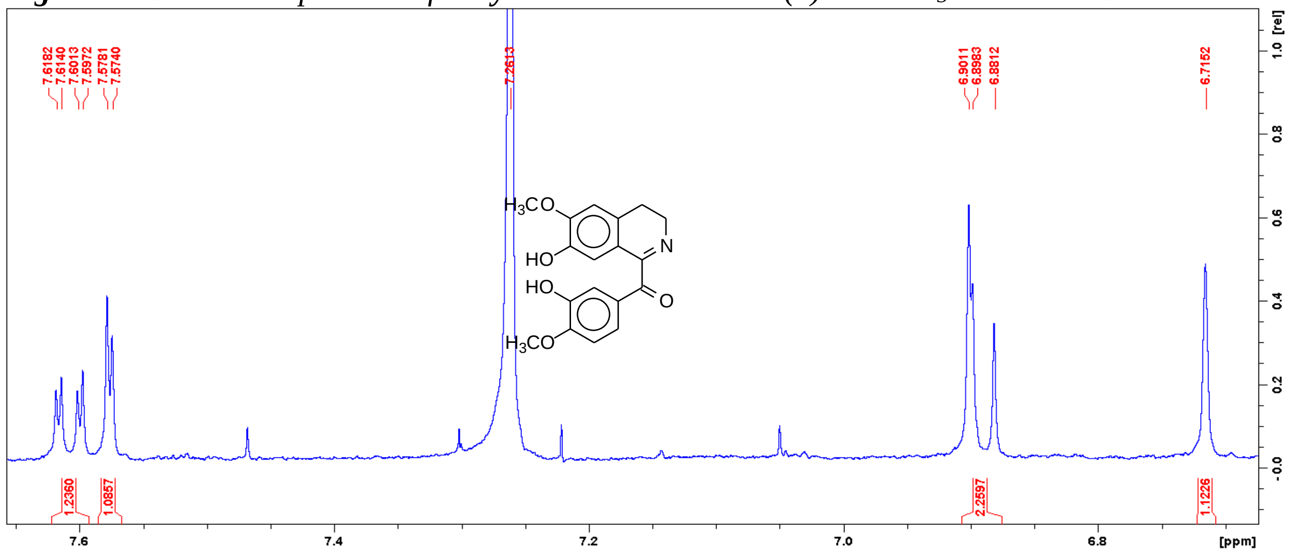

Figure S36. *Expansion (δ 7,65 – 6.68) of the ^{1}H NMR spectrum of* ***dehydro-oxonorreticuline*** *(5) in $CDCl_3$ at 500 MHz.*

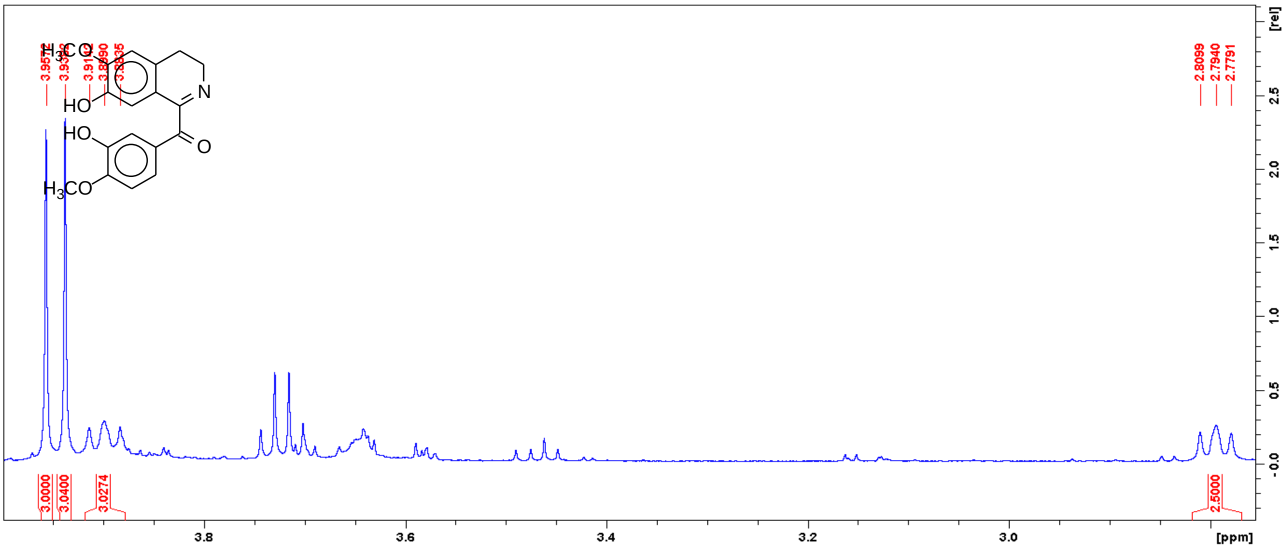

Figure S37. *Expansion (δ 3.99 – 2.76) of the ^{1}H NMR spectrum of* ***dehydro-oxonorreticuline (5)*** *in $CDCl_3$ at 500 MHz.*

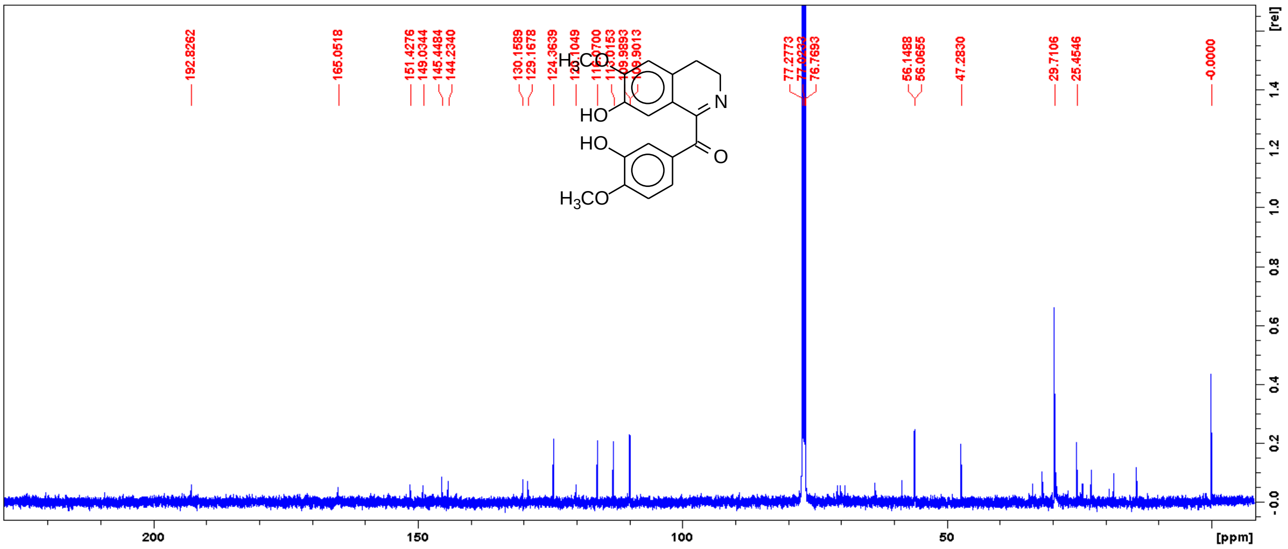

Figure S38. *^{13}C NMR spectrum of* ***dehydro-oxonorreticuline*** *(5) in $CDCl_3$ at 125 MHz.*

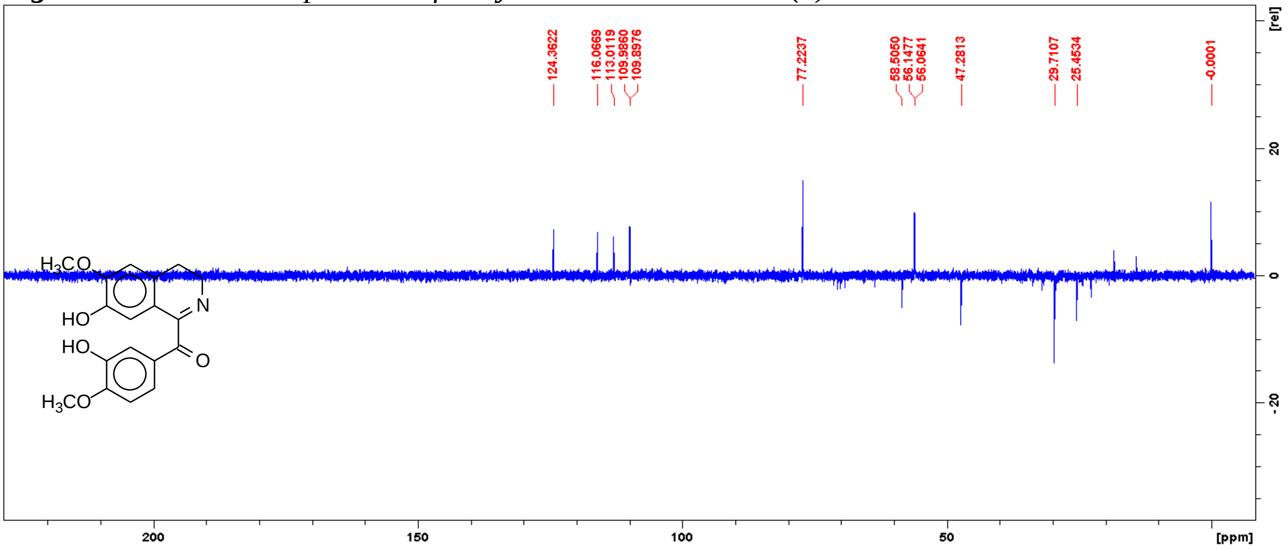

Figure S39. *DEPT 135 spectrum of* ***dehydro-oxonorreticuline*** *(5) in $CDCl_3$ at 125 MHz.*

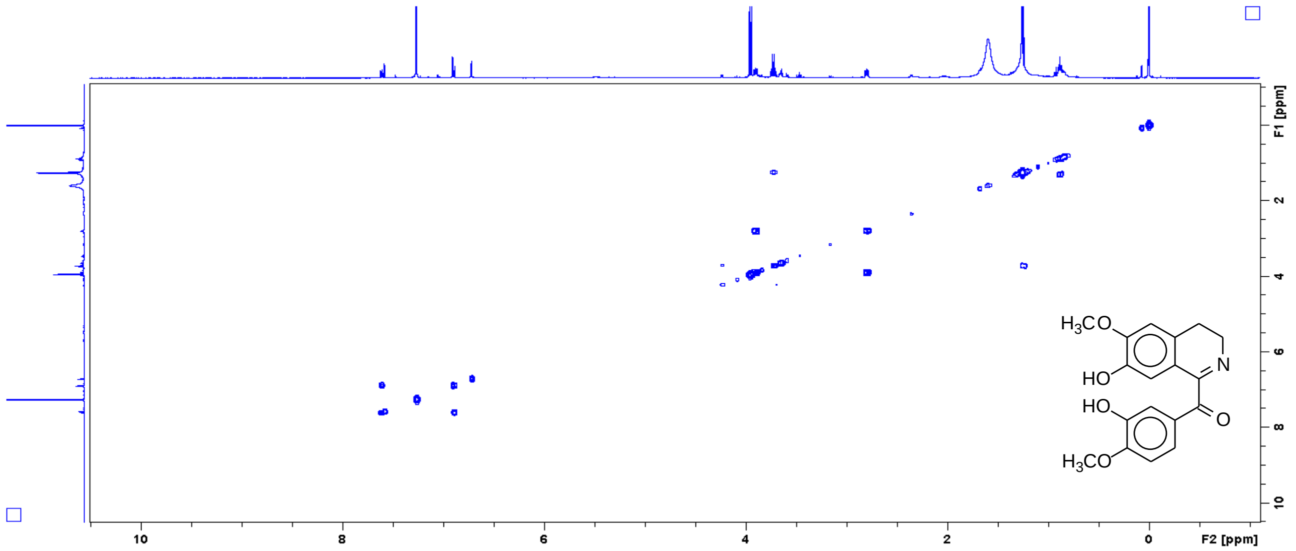

Figure S40. *^1H-^1H correlation map from COSY NMR experiment of* ***dehydro-oxonorreticuline*** ***(5)*** *in $CDCl_3$ at 500 MHz.*

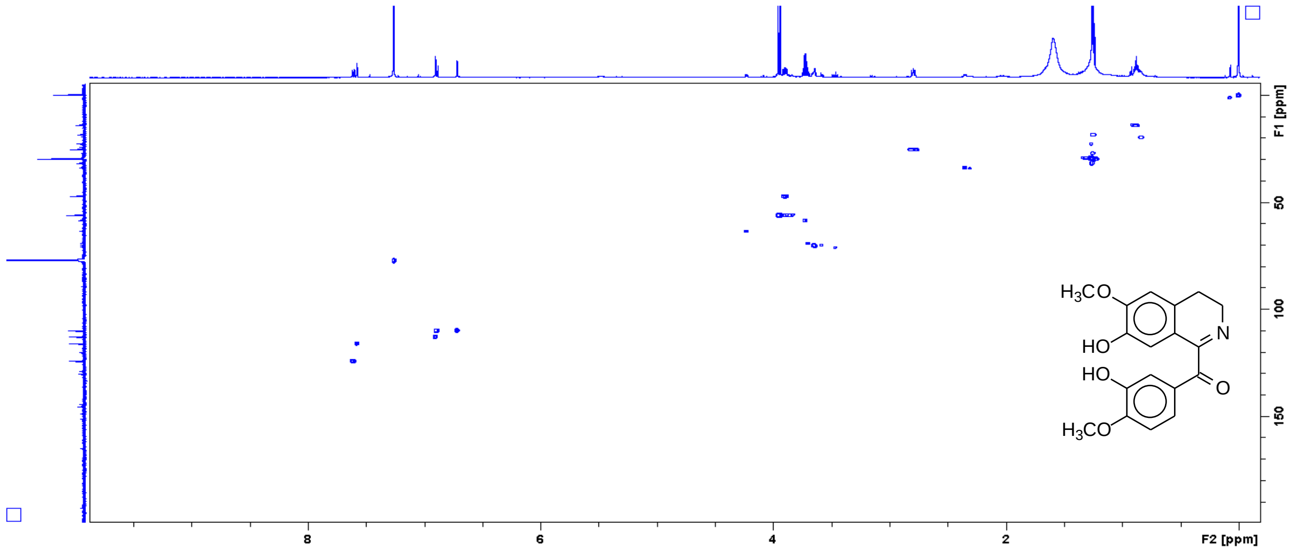

Figure S41. *One-bond ^1H-^{13}C correlation map from HSQC NMR experiment of* ***dehydro-oxonorreticuline*** ***(5)*** *in $CDCl_3$ at 500 (^1H) and 125 MHz (^{13}C), respectively.*

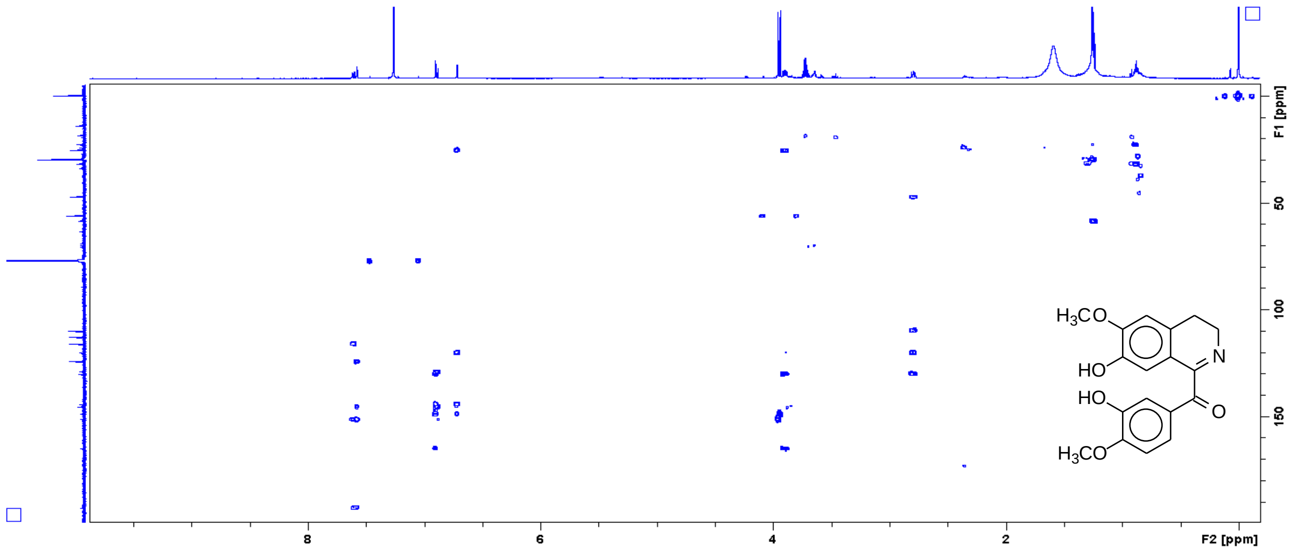

Figure S42. *Long-range ^1H-^{13}C correlation map from HMBC NMR experiment of* ***dehydro-oxonorreticuline*** ***(5)*** *in $CDCl_3$ at 500 (^1H) and 125 MHz (^{13}C), respectively.*

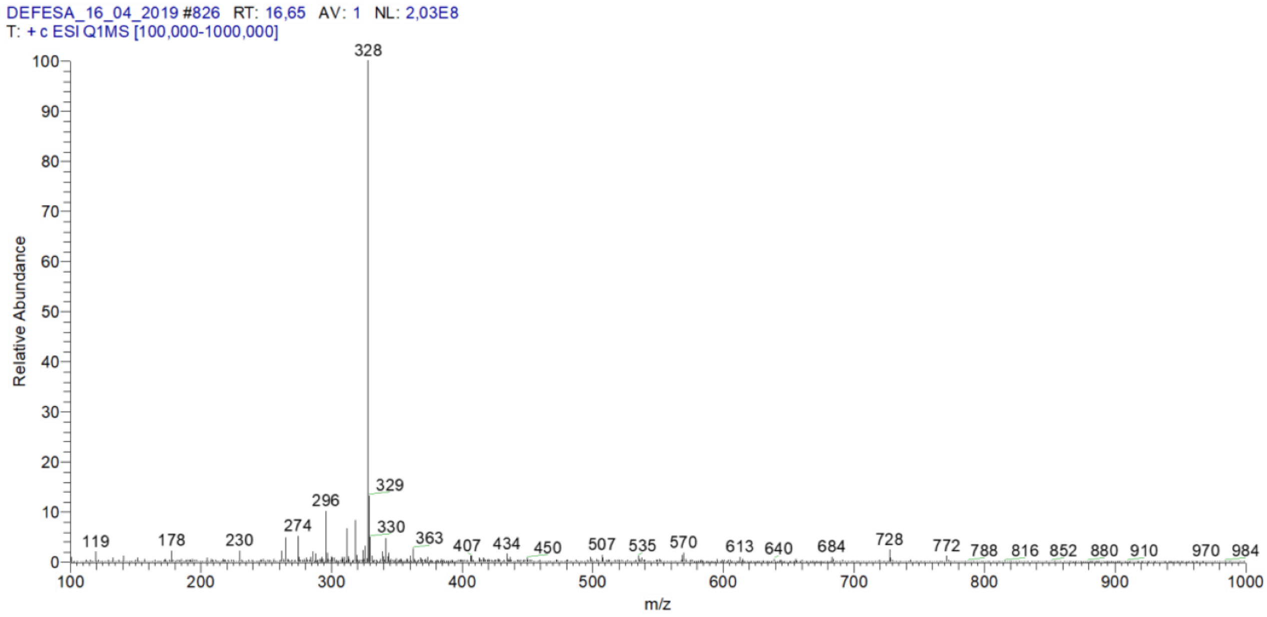

Figure S43. *LR-ESI(+)-MS spectrum of* ***dehydro-oxonorreticuline*** *(5) (m/z 328 [M+H]$^{+}$).*

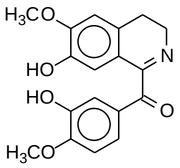

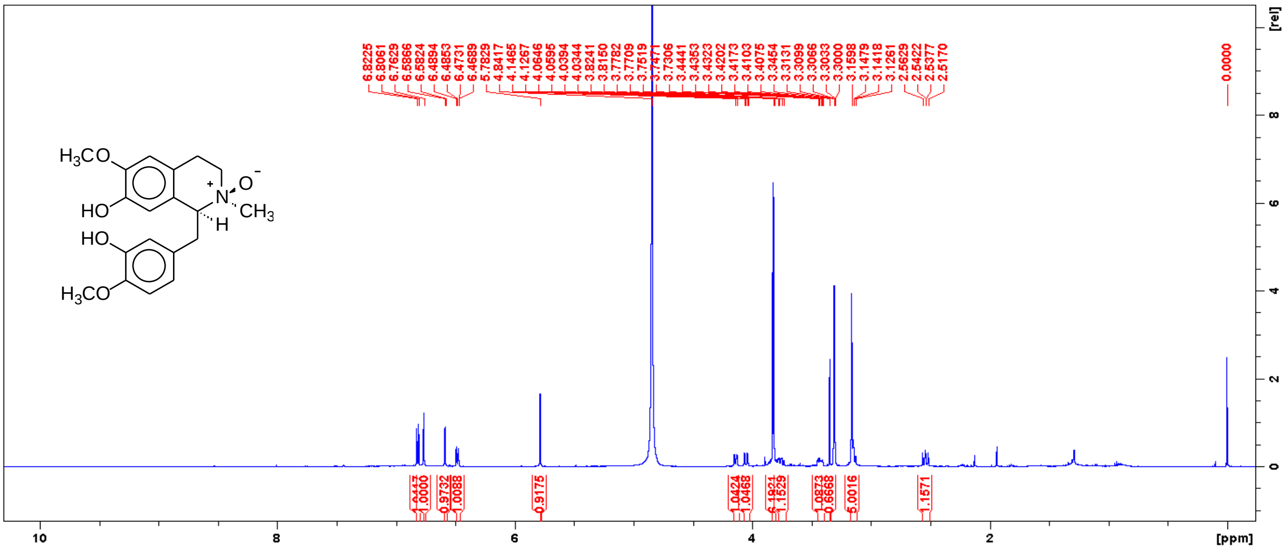

Figure S44. *^{1}H NMR spectrum of* ***(+)-1S,2R-reticuline-Nβ-oxide*** *(6) in CD$_3$OD at 500 MHz.*

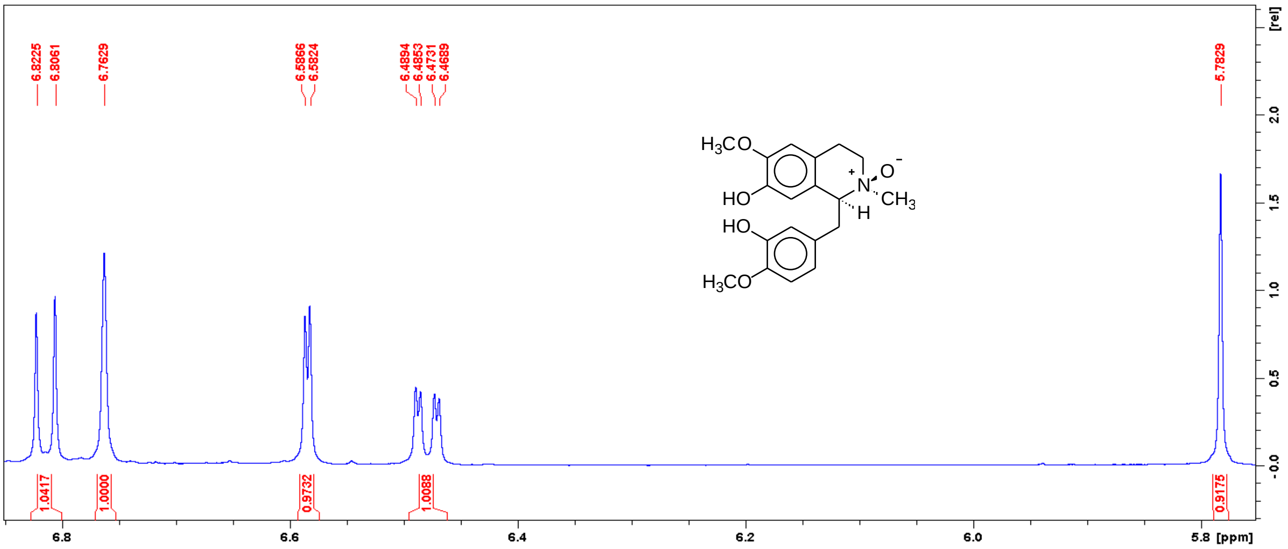

Figure S45. *Expansion (δ 6.84 – 5.75) of the* [1]*H NMR spectrum of* ***(+)-1S,2R-reticuline-N$_\beta$-oxide (6)*** *in CD$_3$OD at 500 MHz.*

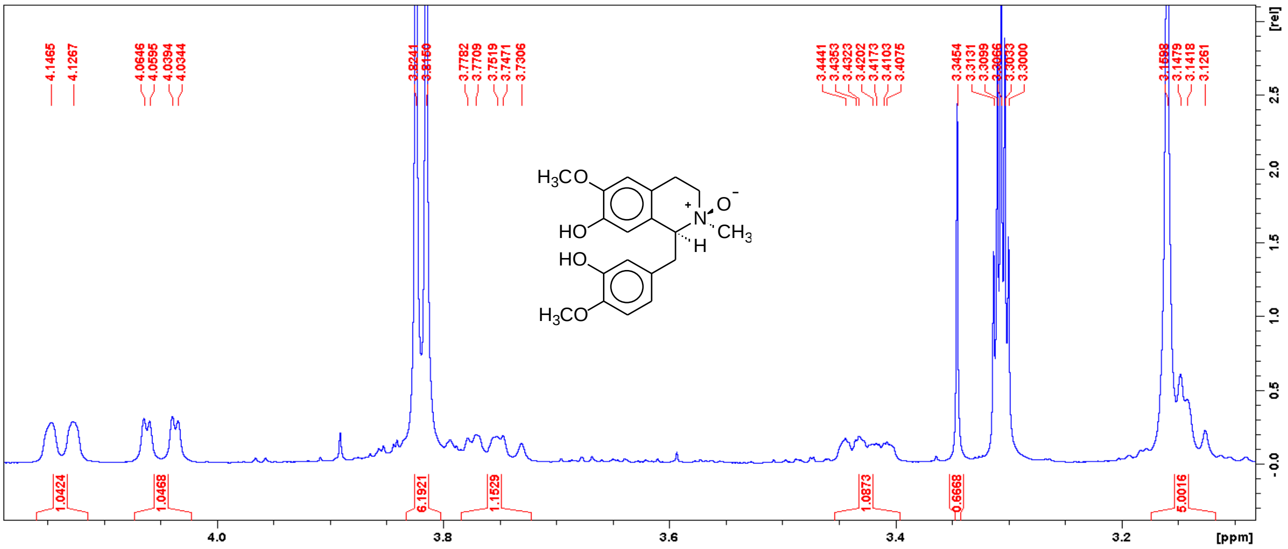

Figure S46. *Expansion (δ 3.88 – 2.41) of the* [1]*H NMR spectrum of* ***(+)-1S,2R-reticuline-N$_\beta$-oxide (6)*** *in CD$_3$OD at 500 MHz.*

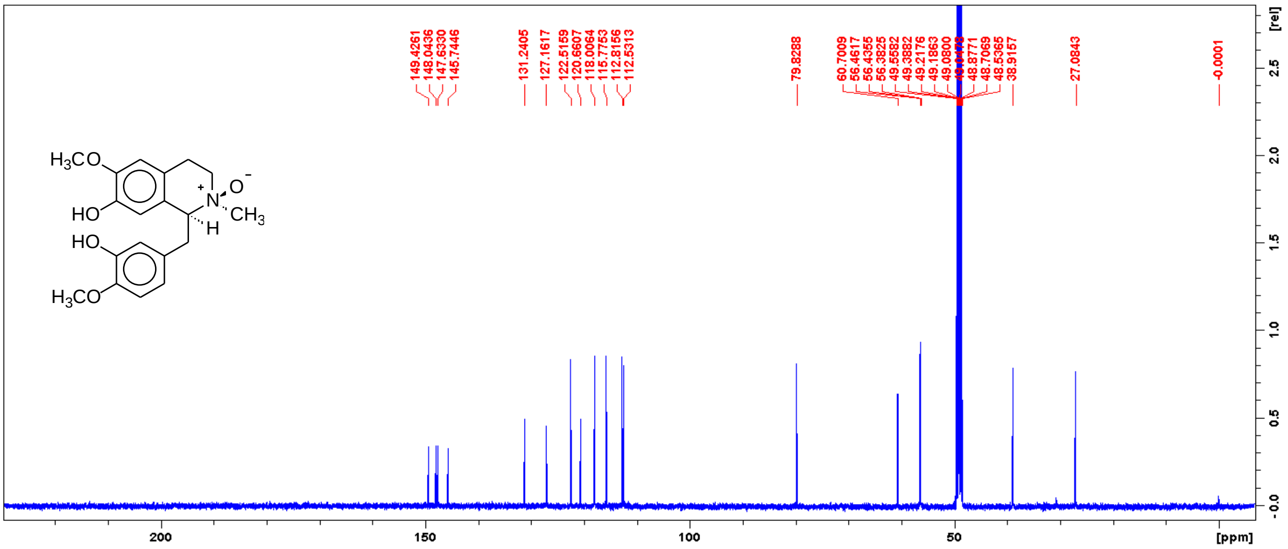

Figure S47. [13]*C NMR spectrum of* ***(+)-1S,2R-reticuline-N$_\beta$-oxide (6)*** *in CD$_3$OD at 125 MHz.*

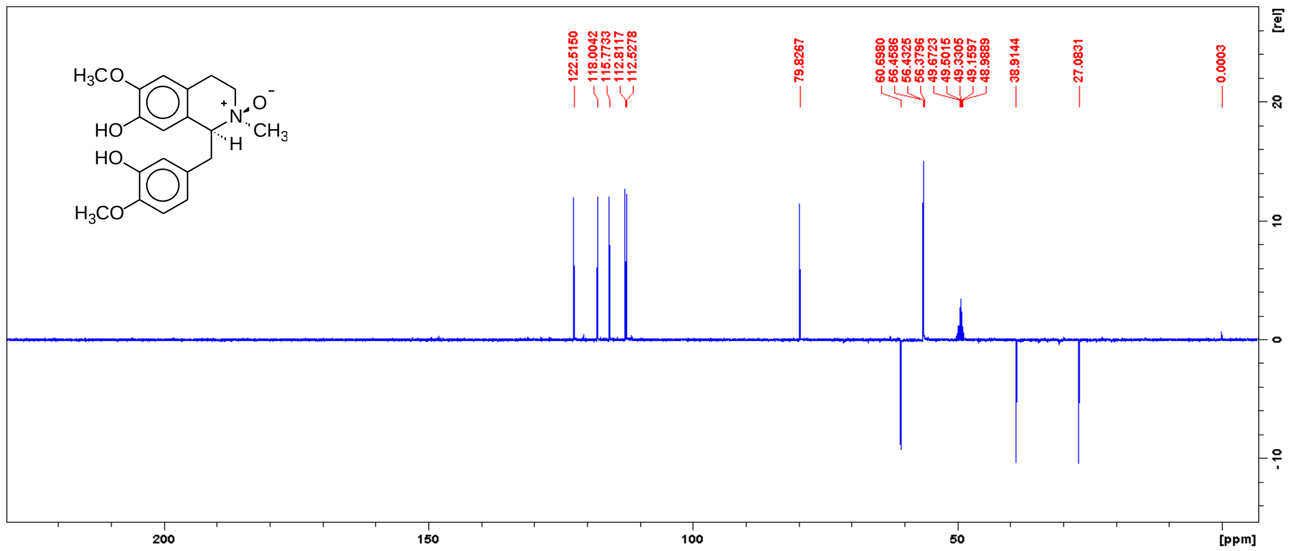

Figure S48. *DEPT 135 spectrum of* ***(+)-1S,2R-reticuline-Nβ-oxide (6)*** *in CD_3OD at 125 MHz.*

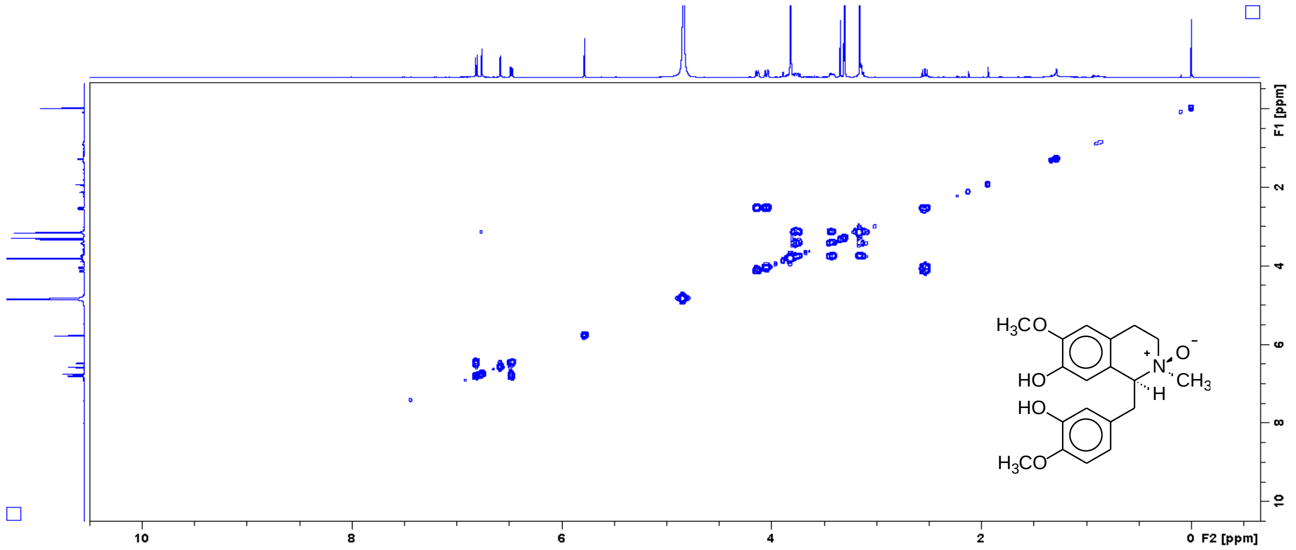

Figure S49. *^1H-^1H correlation map from COSY NMR experiment of* ***(+)-1S,2R-reticuline-Nβ-oxide (6)*** *in CD_3OD at 500 MHz.*

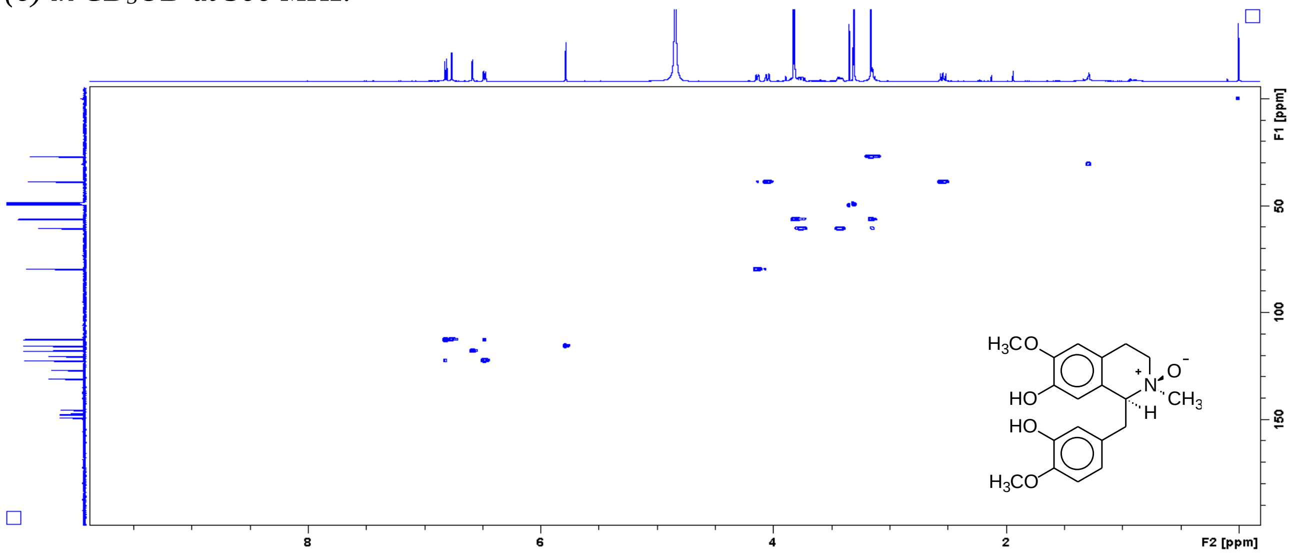

Figure S50. *One-bond ^1H-^{13}C correlation map from HSQC NMR experiment of* ***(+)-1S,2R-reticuline-Nβ-oxide (6)*** *in CD_3OD at 500 (^1H) and 125 MHz (^{13}C), respectively.*

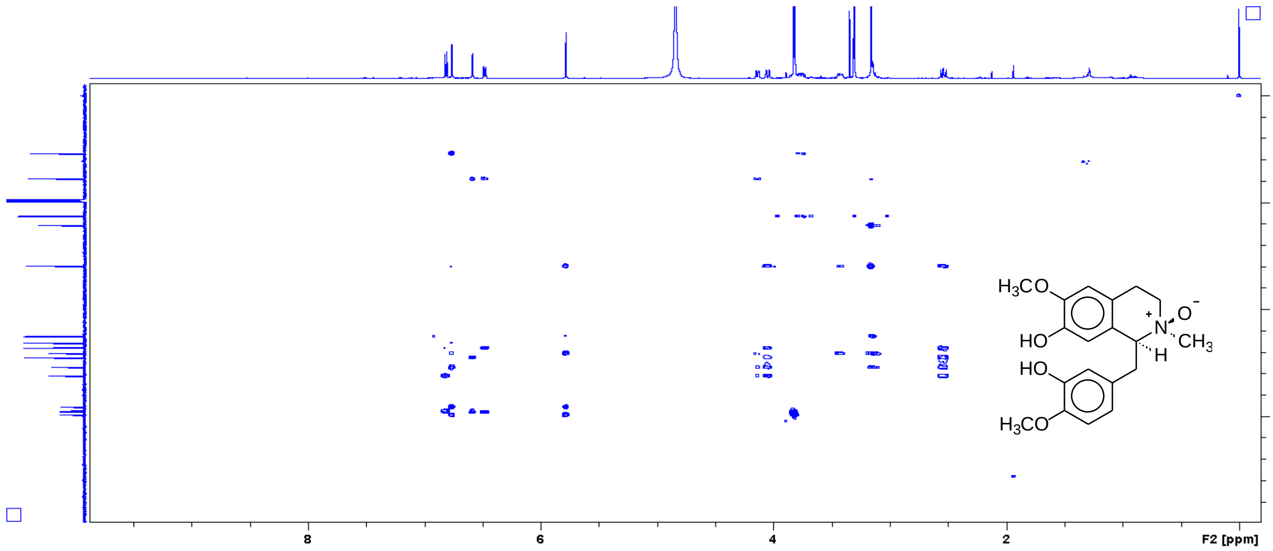

Figure S51. *Long-range ^{1}H-^{13}C correlation map from HMBC NMR experiment of* ***(+)-1S,2R-reticuline-N$_{\beta}$-oxide*** *(**6**) in CD_3OD at 500 (^{1}H) and 125 MHz (^{13}C), respectively.*

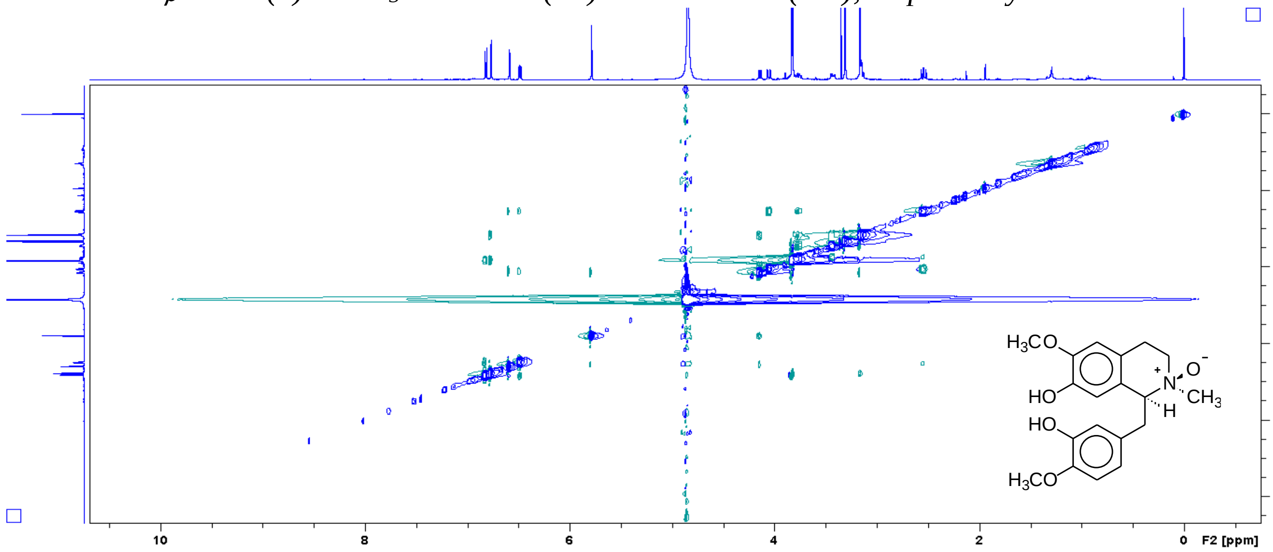

Figure S52. *^{1}H-^{1}H correlation map from NOESY NMR experiment of* ***(+)-1S,2R-reticuline-N$_{\beta}$-oxide*** *(**6**) in CD_3OD at 500 (^{1}H) and 125 MHz (^{13}C), respectively.*

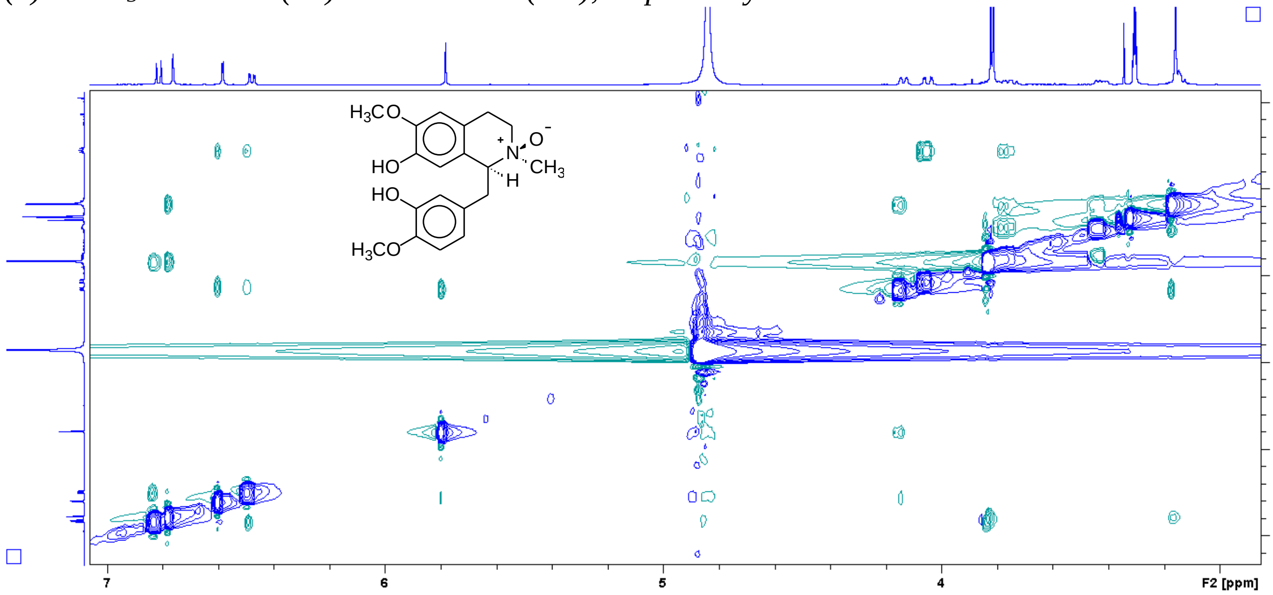

Figure S53. *Expansion of ^{1}H-^{1}H correlation map from NOESY NMR experiment of* ***(+)-1S,2R-reticuline-N$_{\beta}$-oxide*** *(**6**) in CD_3OD at 500 (^{1}H) and 125 MHz (^{13}C), respectively.*

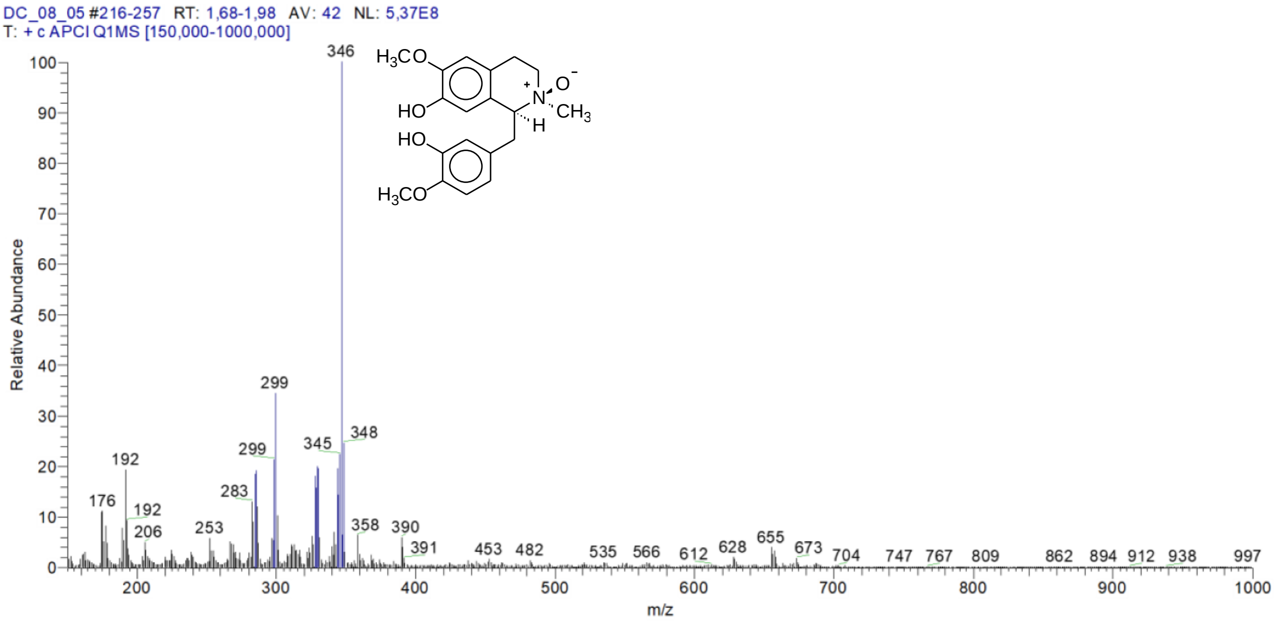

Figure S54. *LR-APCI(+)-MS spectrum of* ***(+)-1S,2R-reticuline-N$_{\beta}$-oxide*** *(**6**) (m/z 346 [M+H]$^+$).*

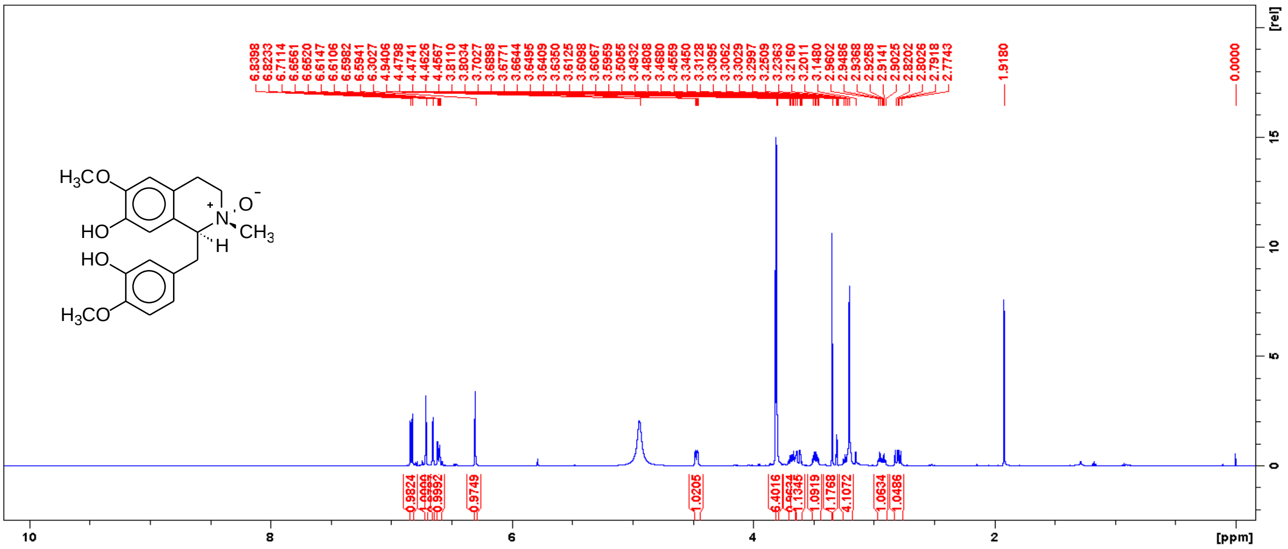

Figure S55. *^{1}H NMR spectrum of* ***(+)-1S,2S-reticuline-N$_{\alpha}$-oxide*** *(**7**) in CD$_3$OD at 500 MHz.*

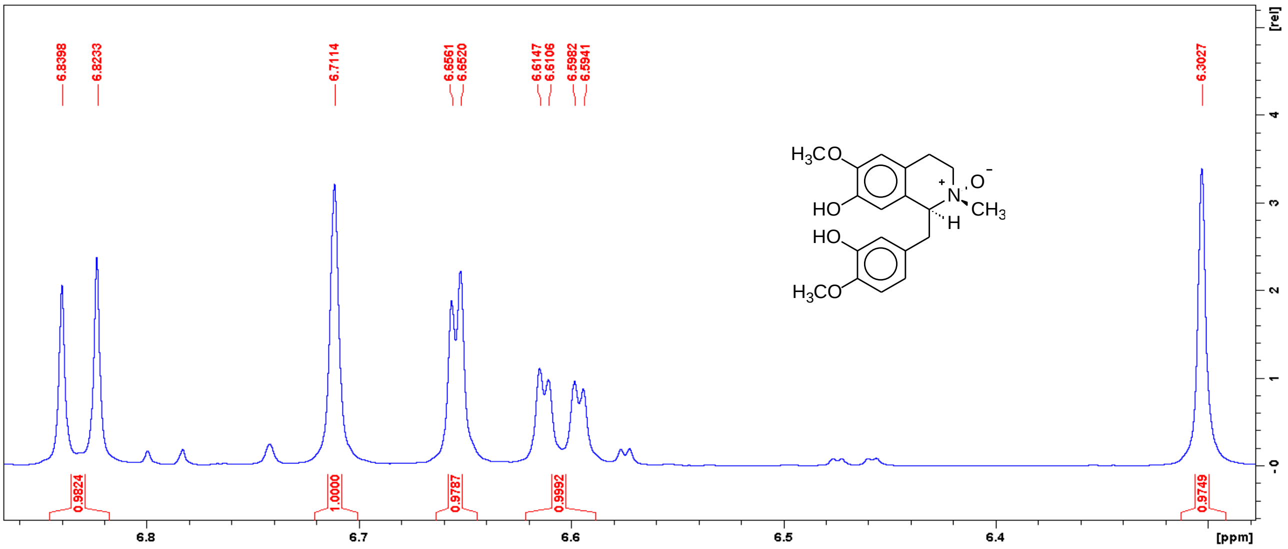

Figure S56. *Expansion (δ 6.86 – 6.28) of the* ^{1}*H NMR spectrum of* ***(+)-1S,2S-reticuline-N$_{\alpha}$-oxide*** *(7) in* CD_3OD *at 500 MHz.*

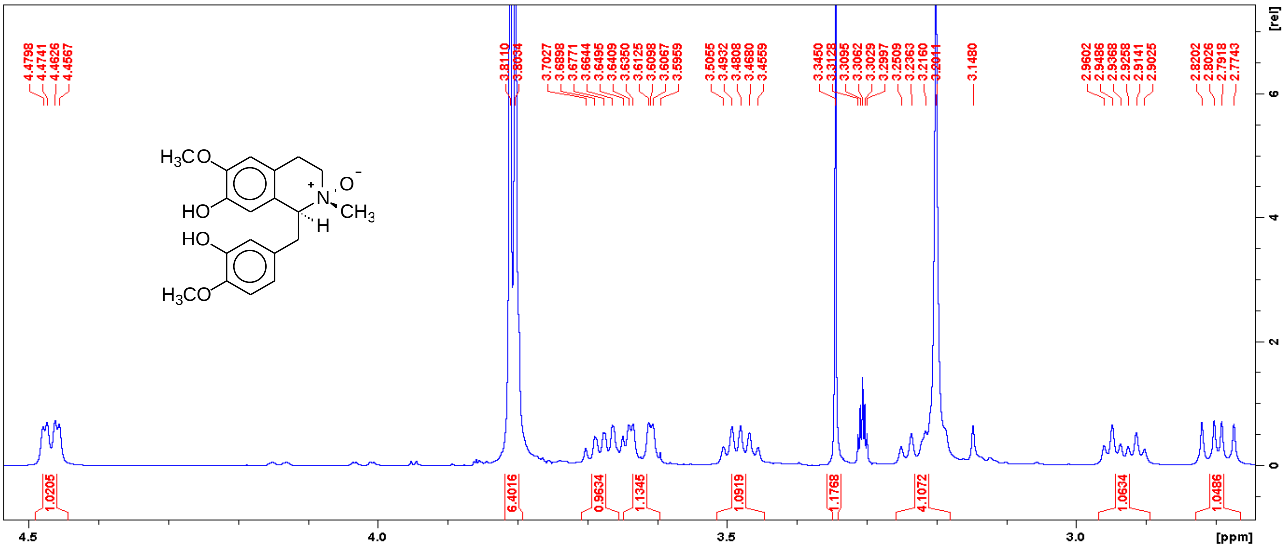

Figure S57. *Expansion (δ 4.53 – 2.75) of the* ^{1}*H NMR spectrum of* ***(+)-1S,2S-reticuline-N$_{\alpha}$-oxide*** *(7) in* CD_3OD *at 500 MHz.*

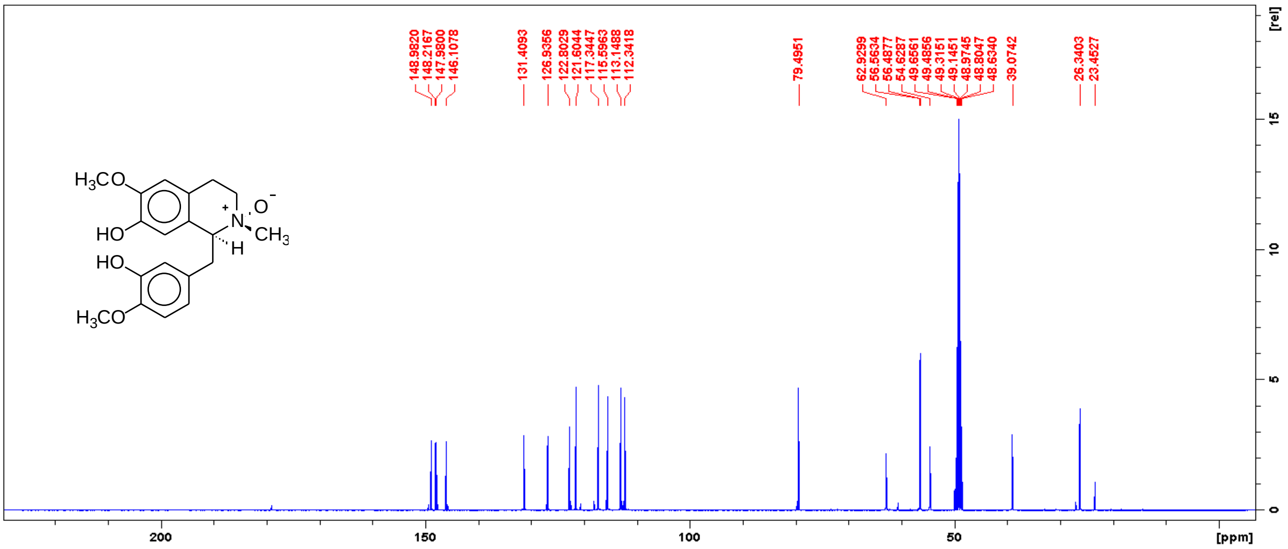

Figure S58. ^{13}*C NMR spectrum of* ***(+)-1S,2S-reticuline-N$_{\alpha}$-oxide*** *(7) in* CD_3OD *at 125 MHz.*

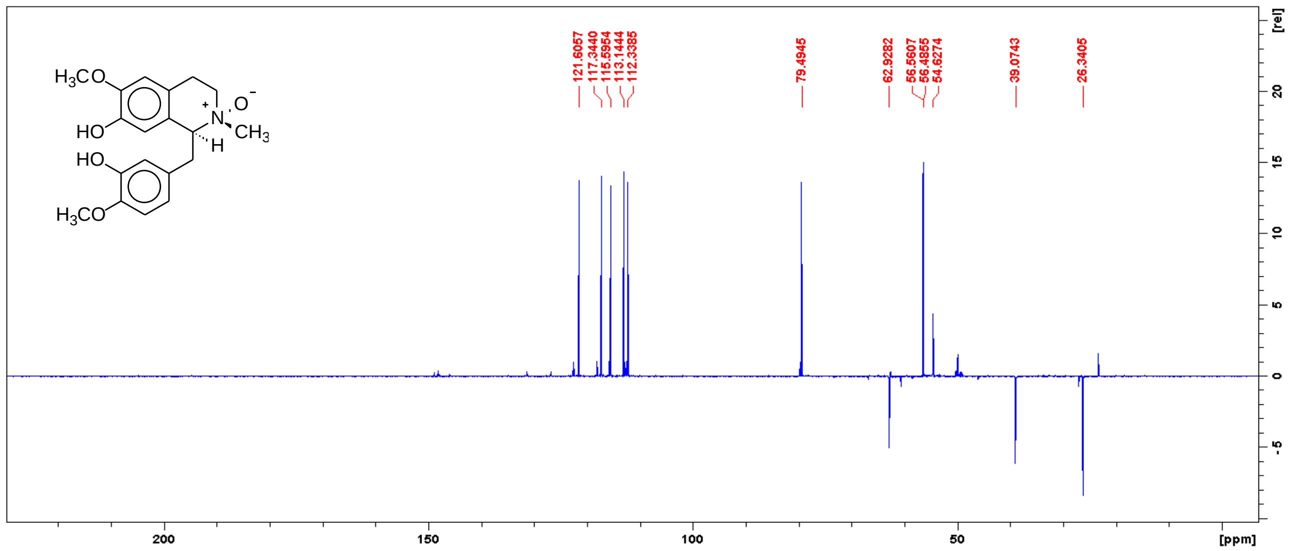

Figure S59. *DEPT 135 spectrum of* ***(+)-1S,2S-reticuline-Nα-oxide*** *(7) in CD3OD at 125 MHz.*

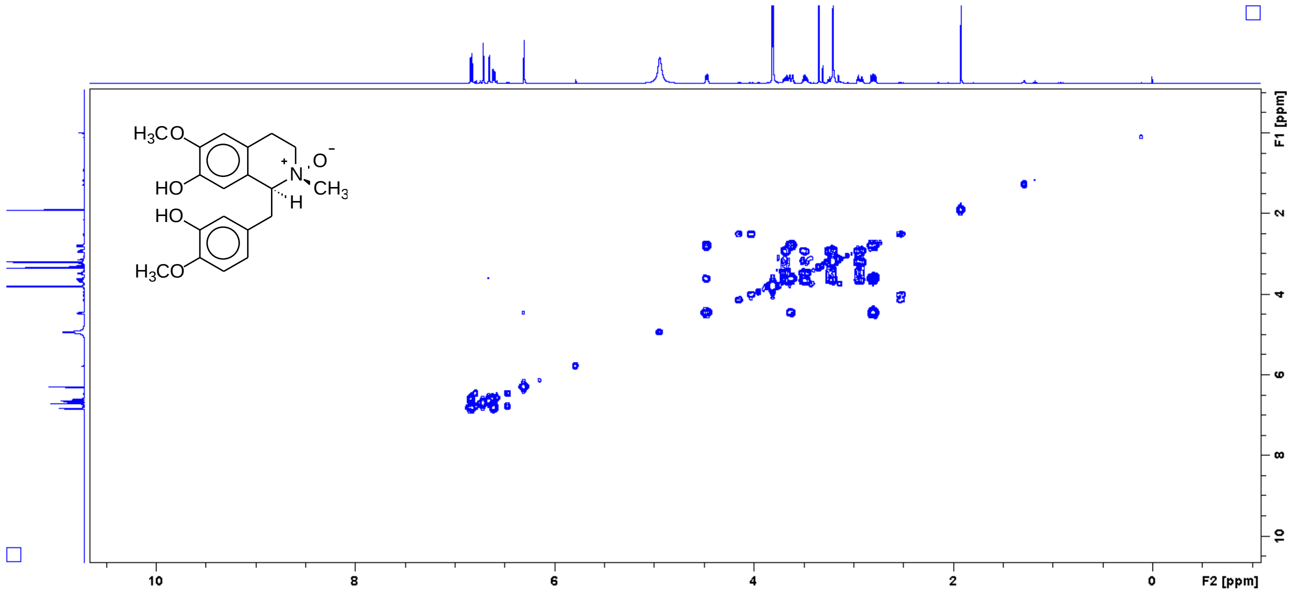

Figure S60. *^{1}H-^{1}H correlation map from COSY NMR experiment of* ***(+)-1S,2S-reticuline-Nα-oxide*** *(7) in CD3OD at 500 MHz.*

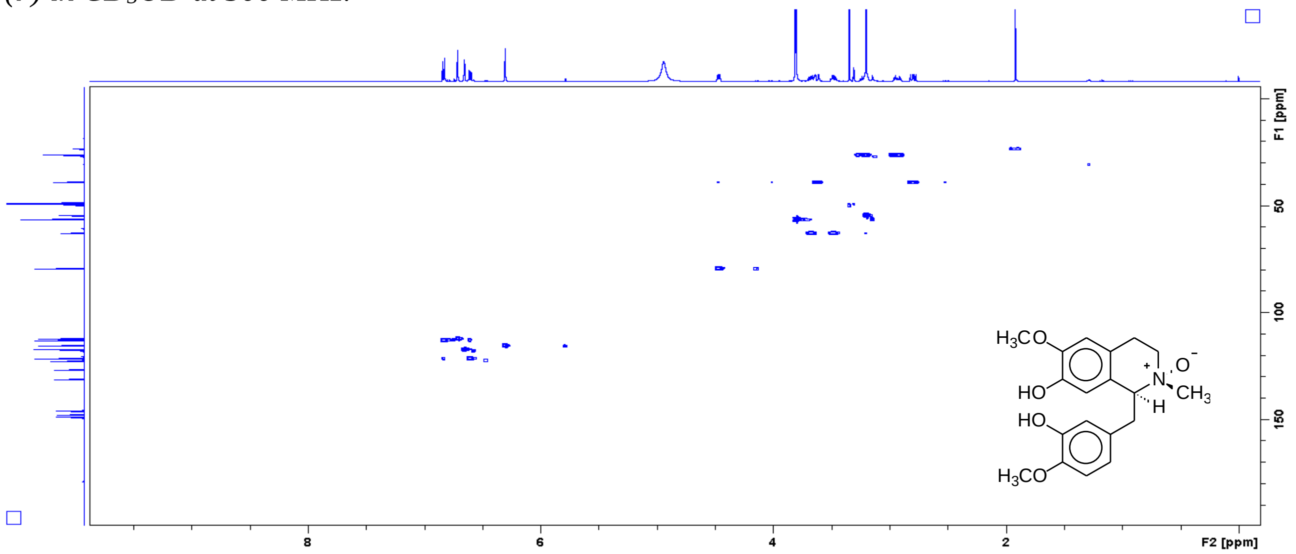

Figure S61. *One-bond ^{1}H-^{13}C correlation map from HSQC NMR experiment of* ***(+)-1S,2S-reticuline-Nα-oxide*** *(7) in CD3OD at 500 (^{1}H) and 125 MHz (^{13}C), respectively.*

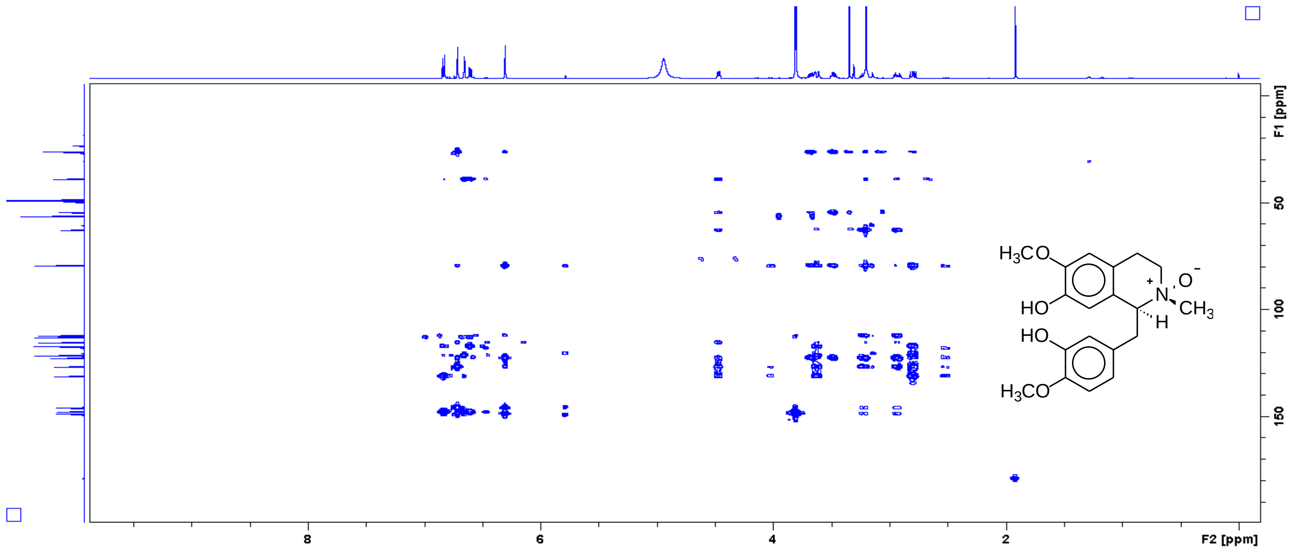

Figure S62. *Long-range* ^{1}*H-*^{13}*C correlation map from HMBC NMR experiment of* ***(+)-1S,2S-reticuline-N***$_{\alpha}$***-oxide*** *(7) in* CD_3OD *at 500 (*^{1}*H) and 125 MHz (*^{13}*C), respectively.*

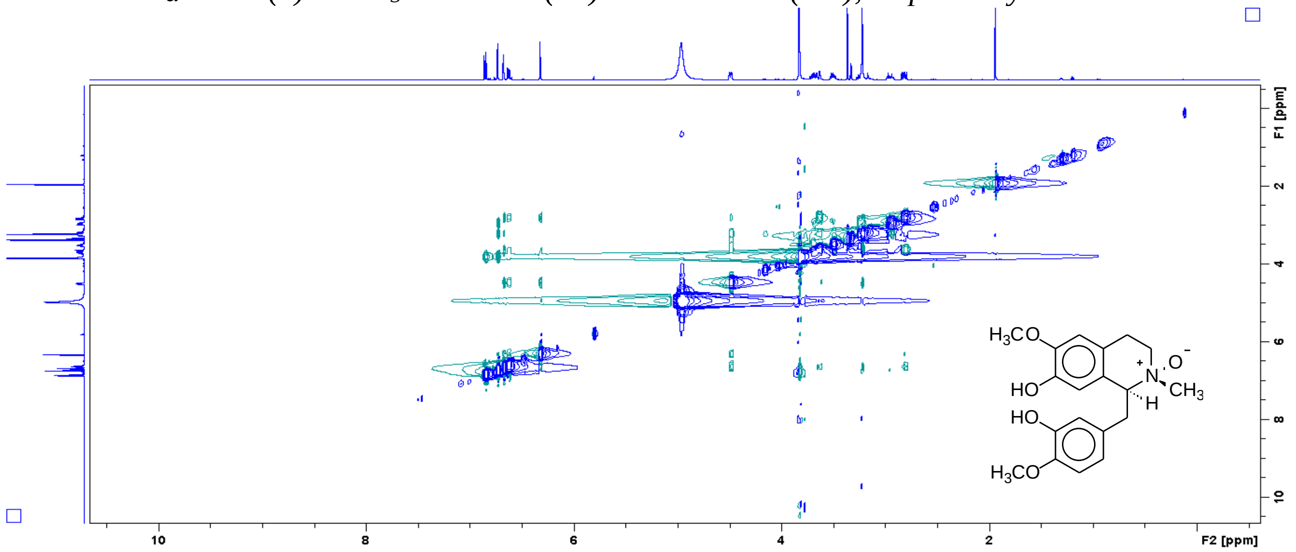

Figure S63. ^{1}*H-*^{1}*H correlation map from NOESY NMR experiment of* ***(+)-1S,2S-reticuline-N***$_{\alpha}$***-oxide*** *(7) in* CD_3OD *at 500 (*^{1}*H) and 125 MHz (*^{13}*C), respectively.*

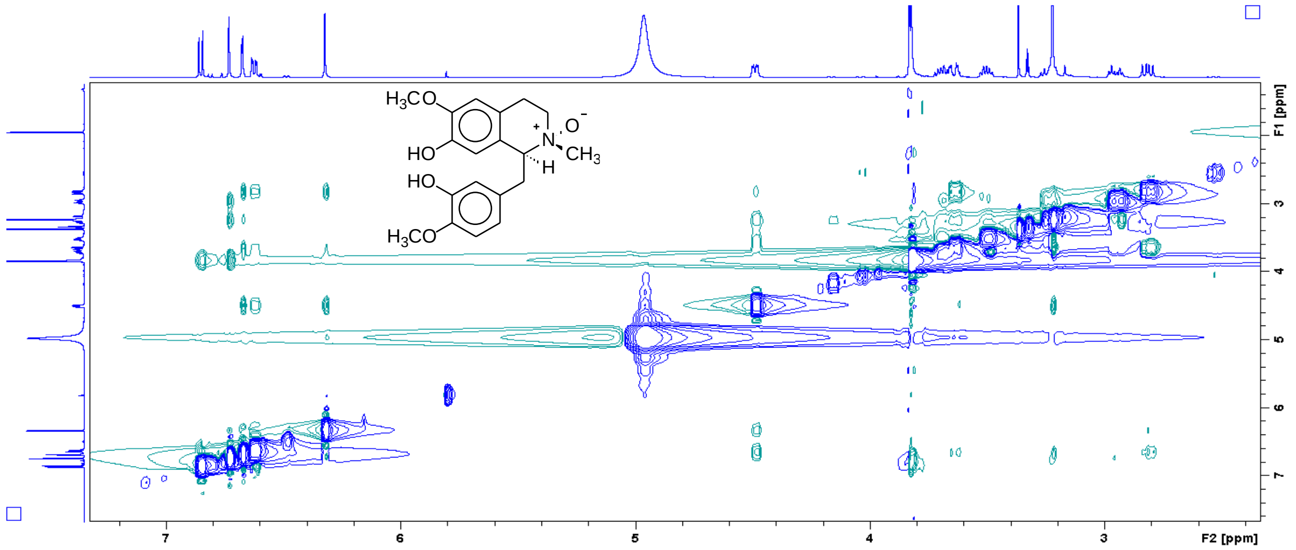

Figure S64. *Expansion of* ^{1}*H-*^{1}*H correlation map from NOESY NMR experiment of* ***(+)-1S,2S-reticuline-N***$_{\alpha}$***-oxide*** *(7) in* CD_3OD *at 500 (*^{1}*H) and 125 MHz (*^{13}*C), respectively.*

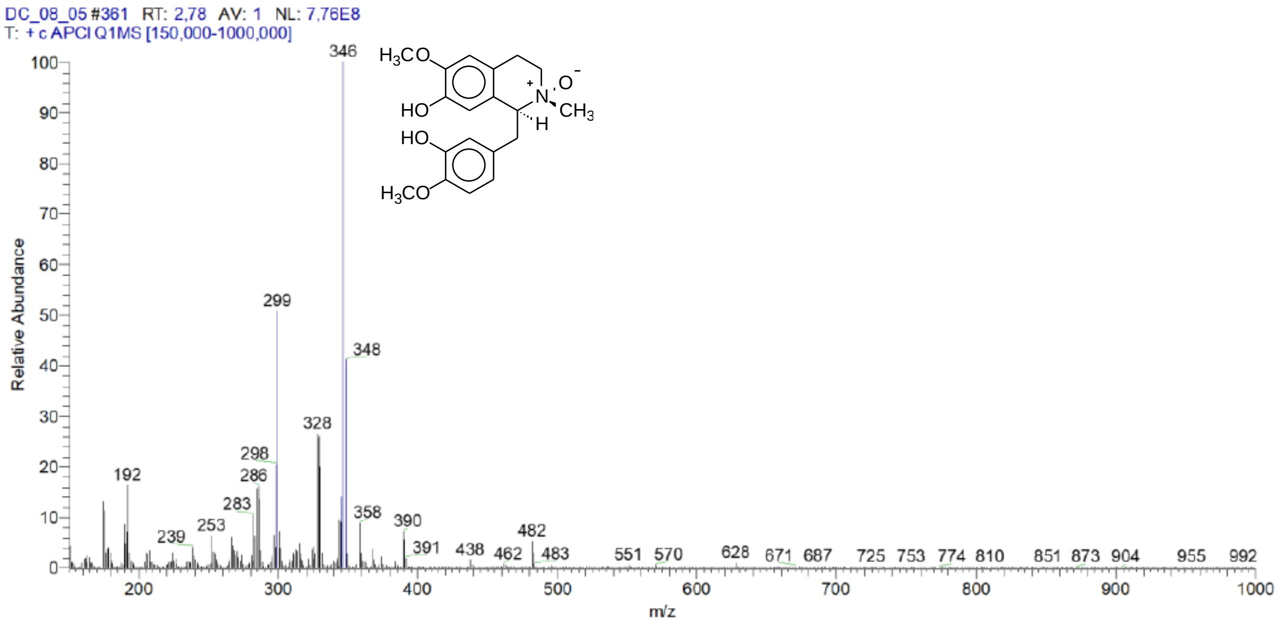

Figure S65. *LR-APCI(+)-MS spectrum of* ***(+)-1S,2S-reticuline-N$_\alpha$-oxide*** *(****7****) (m/z 345 $[M+H]^+$).*

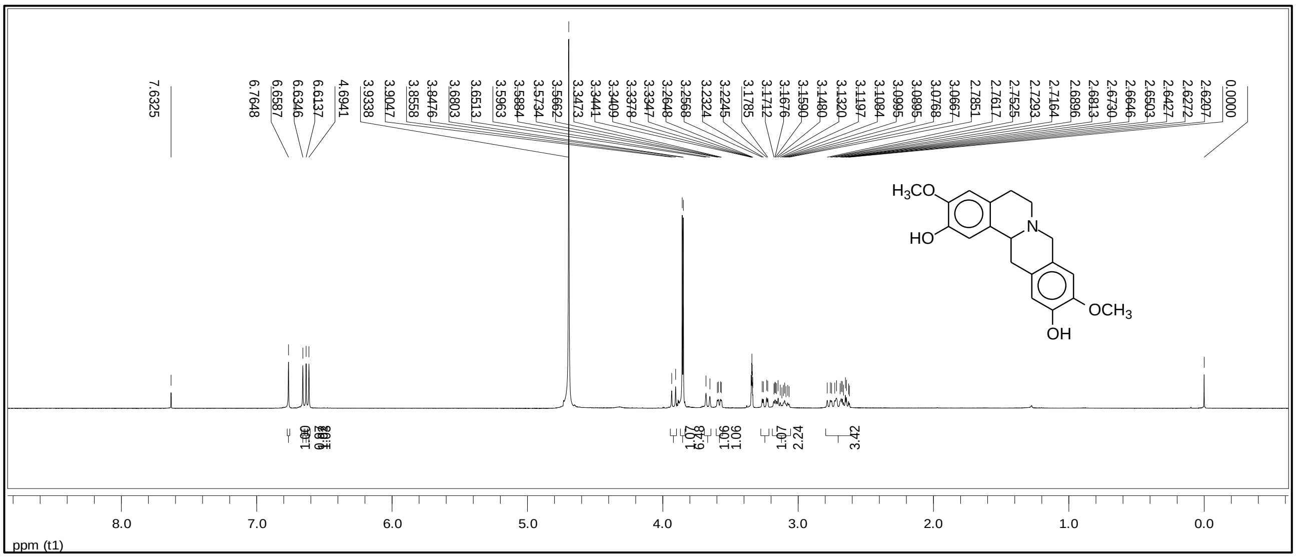

Figure S66. *^{1}H NMR spectrum of* ***coreximine*** *(****8****) in $CDCl_3$ plus drops of CD_3OD at 500 MHz.*

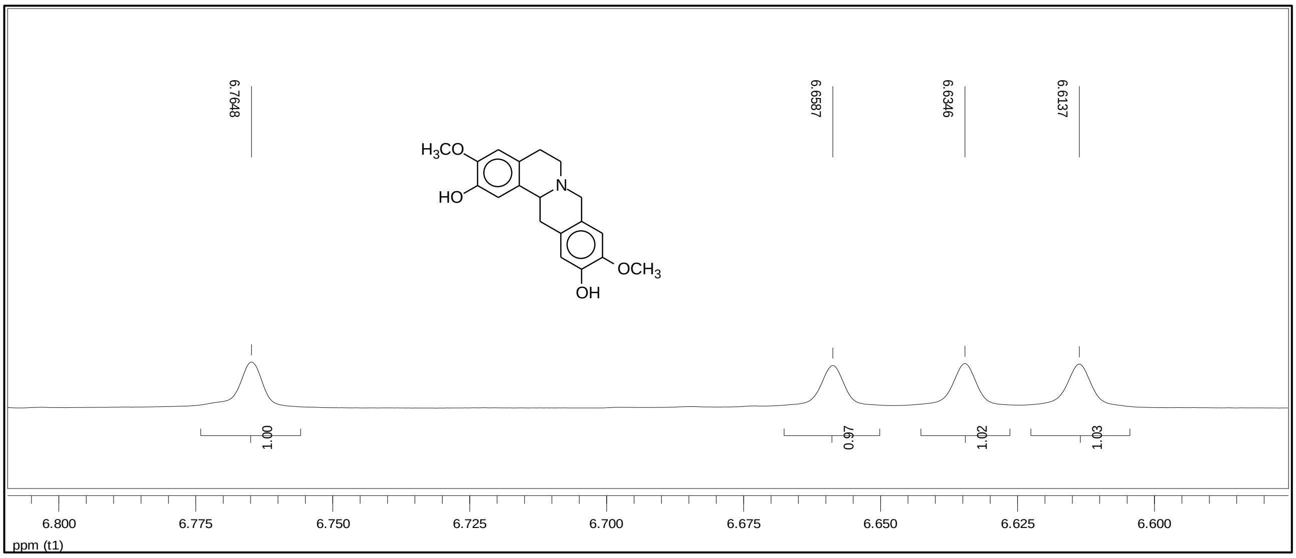

Figure S67. *Expansion (δ 6.80 – 6.57) of the* [1]*H NMR spectrum of* ***coreximine (8)*** *in* $CDCl_3$ *plus drops of* CD_3OD *at 500 MHz.*

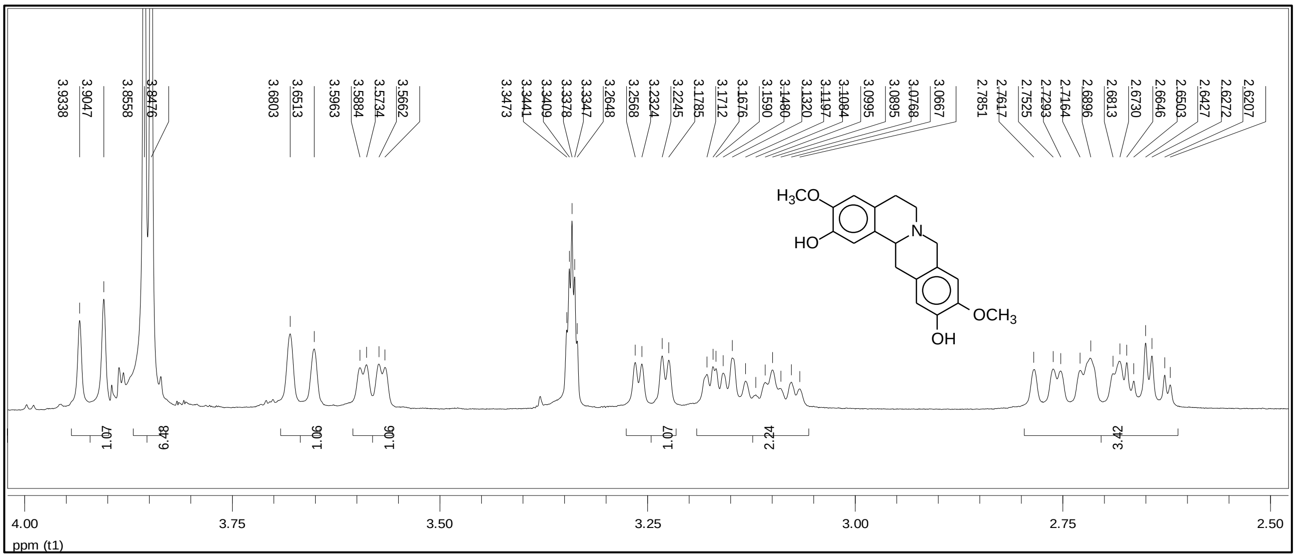

Figure S68. *Expansion (δ 4.00 – 2.49) of the* [1]*H NMR spectrum of* ***coreximine (8)*** *in* $CDCl_3$ *plus drops of* CD_3OD *at 500 MHz.*

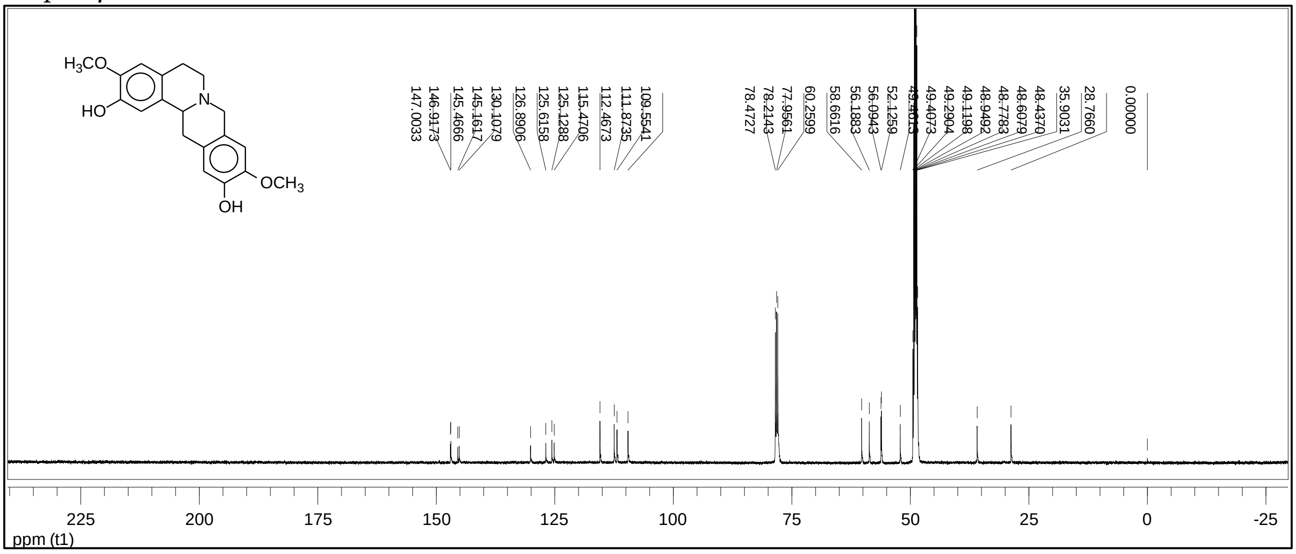

Figure S69. [13]*C NMR spectrum of* ***coreximine (8)*** *in* $CDCl_3$ *plus drops of* CD_3OD *in* CD_3OD *at 125 MHz.*

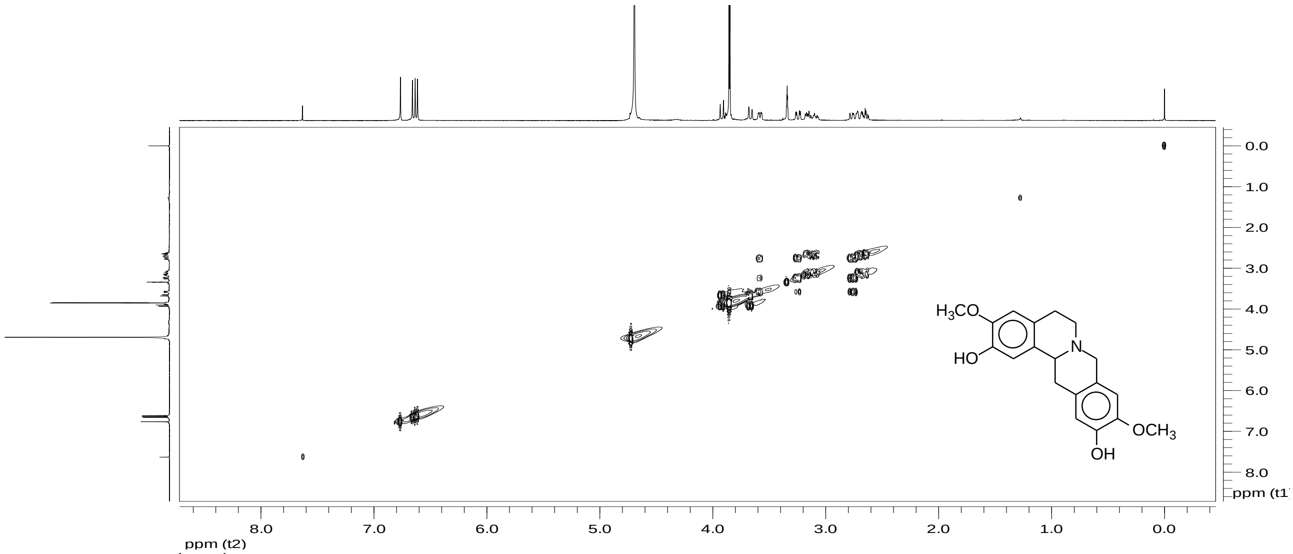

Figure S70. *^{1}H-^{1}H correlation map from COSY NMR experiment of* ***coreximine (8)*** *in $CDCl_3$ plus drops of CD_3OD in CD_3OD at 500 MHz.*

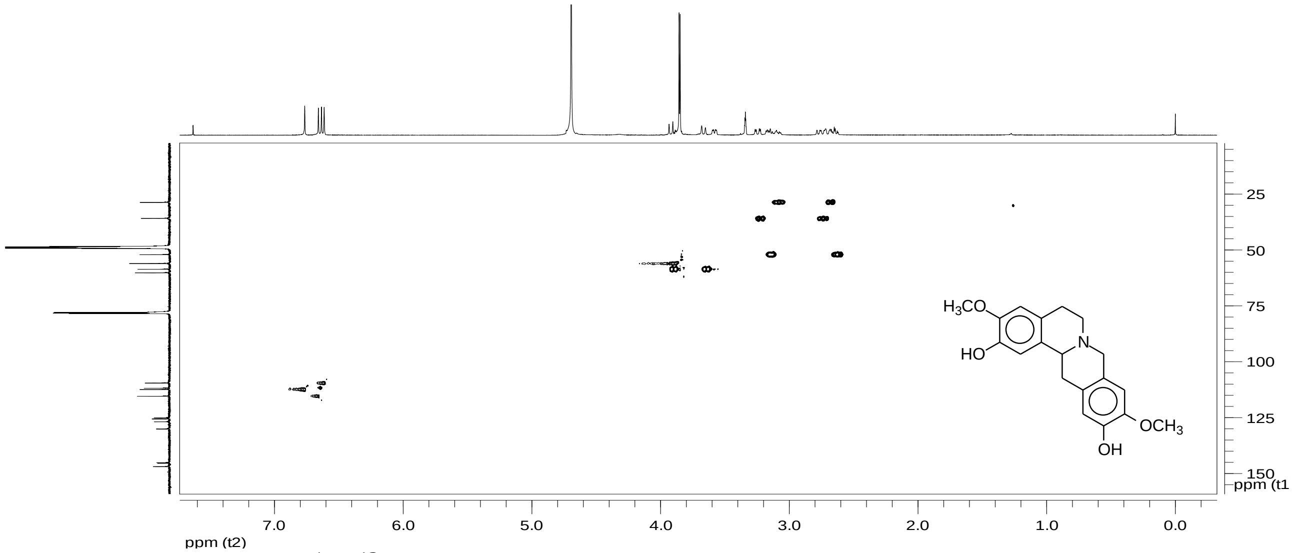

Figure S71. *One-bond ^{1}H-^{13}C correlation map from HSQC NMR experiment of* ***coreximine (8)*** *in $CDCl_3$ plus drops of CD_3OD at 500 (^{1}H) and 125 MHz (^{13}C), respectively.*

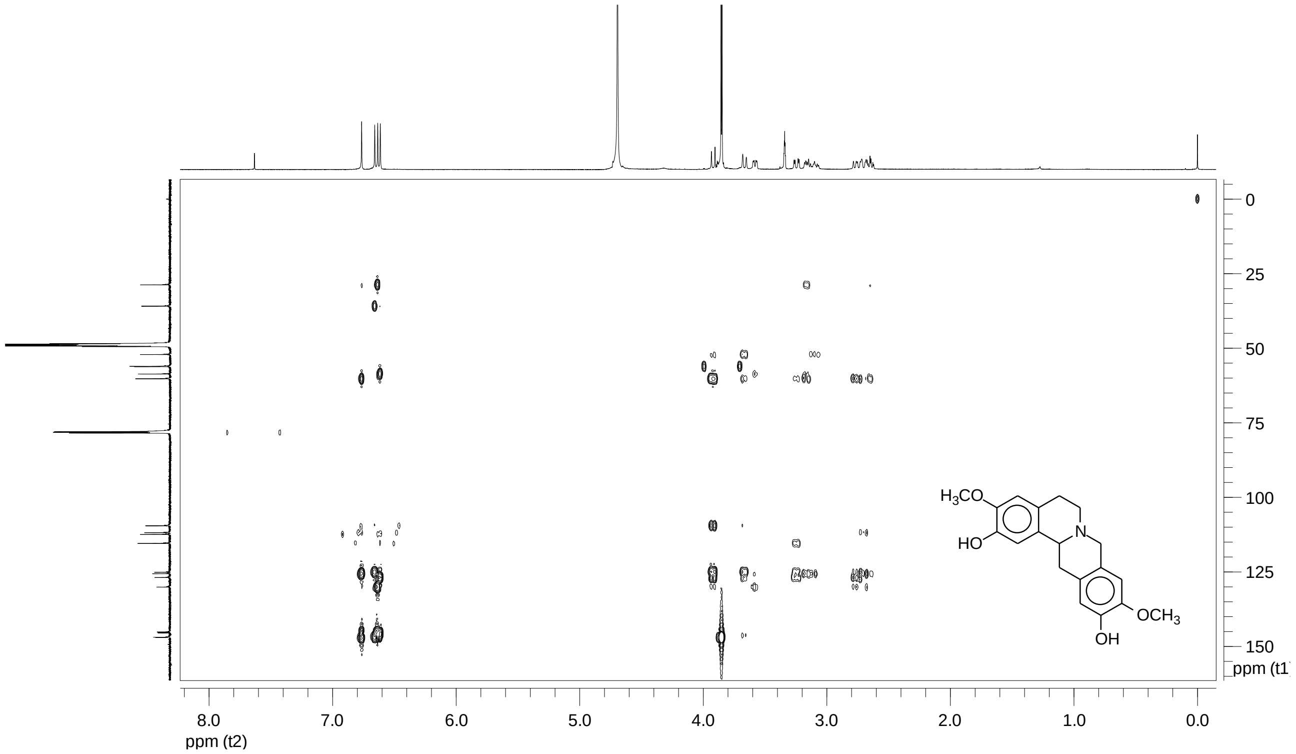

Figure S72. *Long-range* ^{1}H*-*^{13}C *correlation map from HMBC NMR experiment of* ***coreximine (8)*** *in* $CDCl_3$ *plus drops of* CD_3OD *at 500 (*^{1}H*) and 125 MHz (*^{13}C*), respectively.*

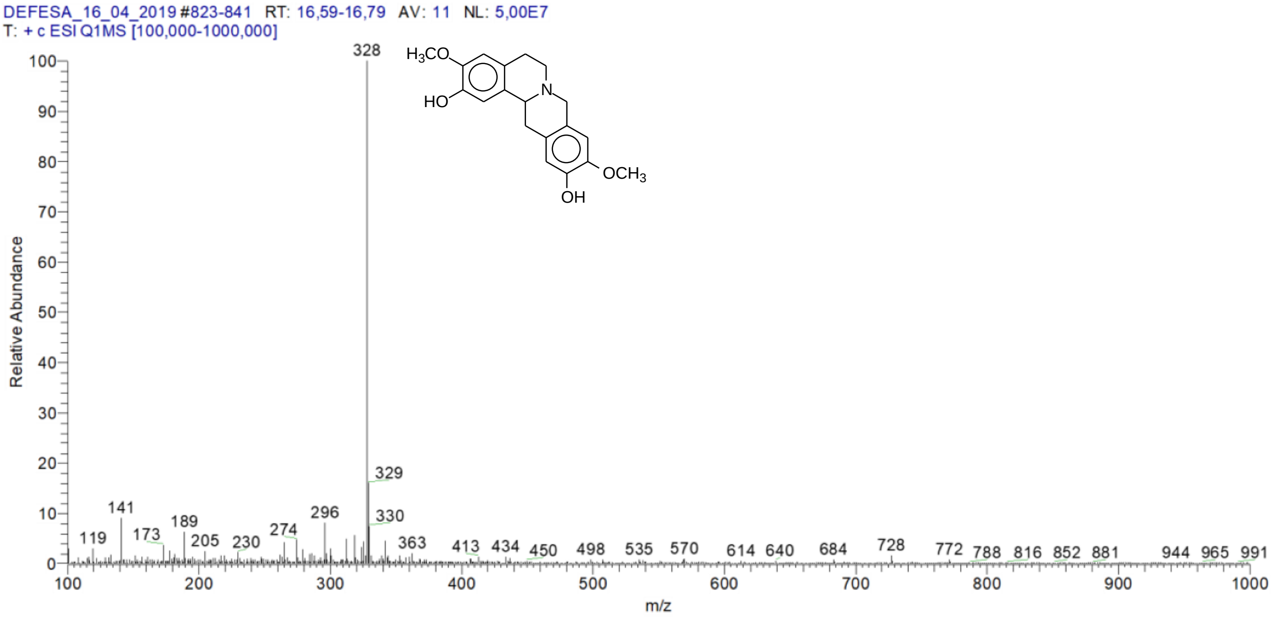

Figure S73. *LR-ESI(+)-MS spectrum of* ***coreximine (8)*** *(m/z 328* $[M+H]^{+}$*).*

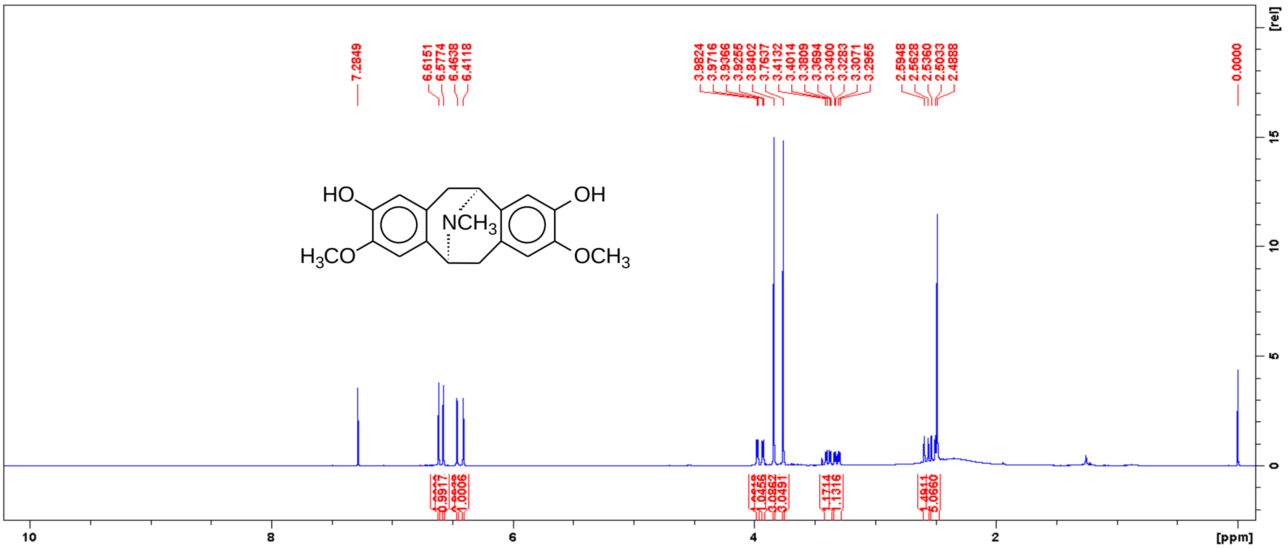

Figure S74. *^{1}H NMR spectrum of* ***bisnorargemonine*** *(****9****) in* $CDCl_3$ *plus drop of* CD_3OD *at 500 MHz.*

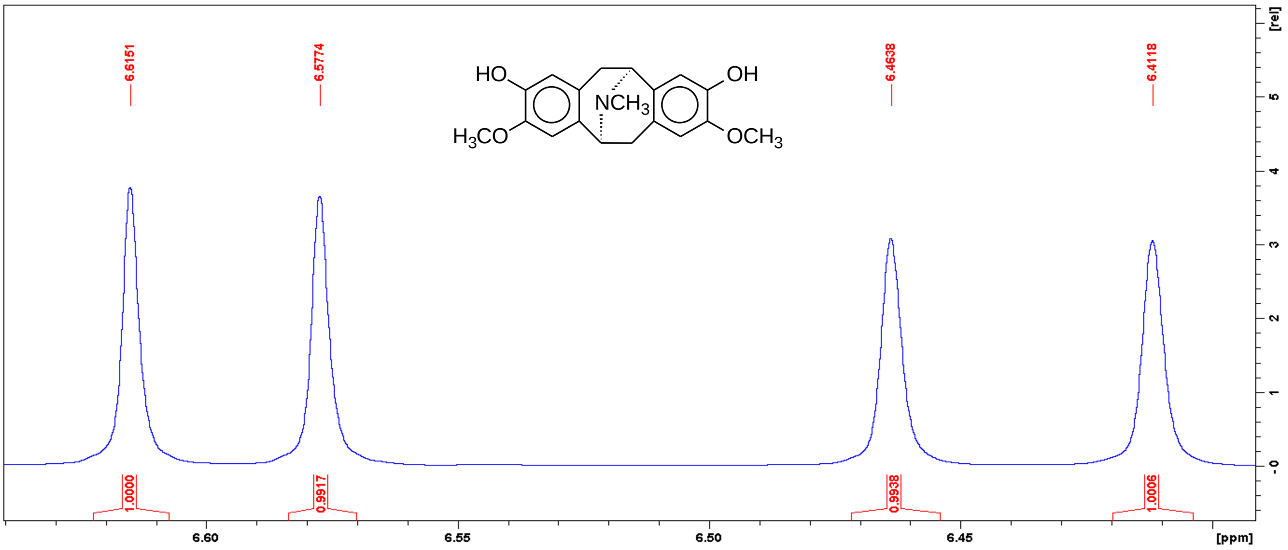

Figure S75. *Expansion (δ 6.63 – 6.39) of the ^{1}H NMR spectrum of* ***bisnorargemonine*** *(****9****) in* $CDCl_3$ *plus drop of* CD_3OD *at 500 MHz.*

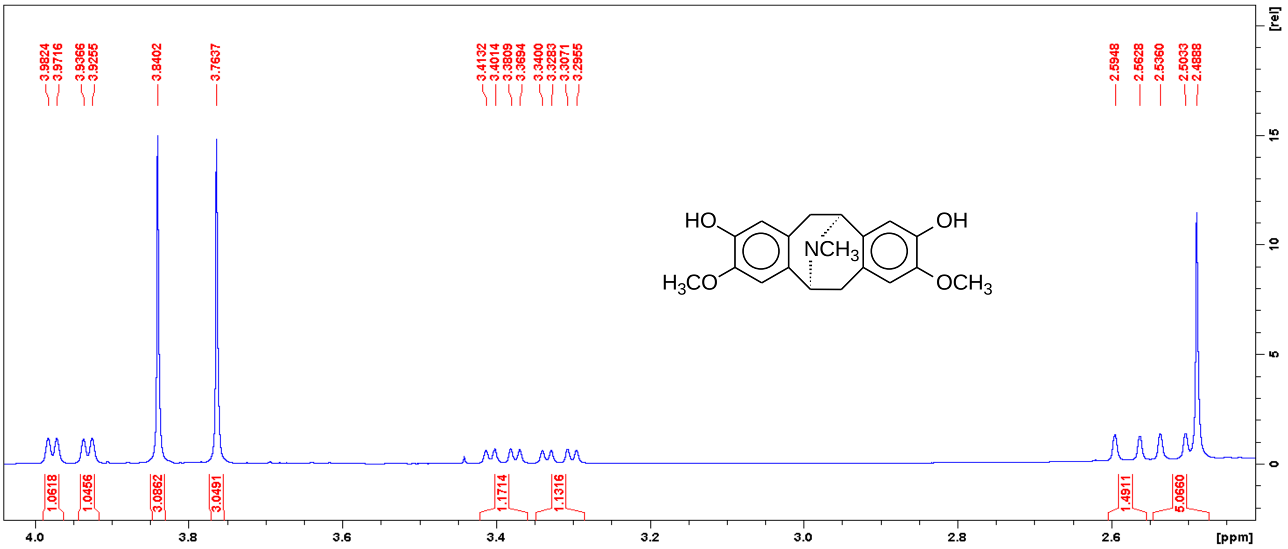

Figure S76. *Expansion (δ 4.03 – 2.42) of the ^{1}H NMR spectrum of* ***bisnorargemonine*** *(****9****) in* $CDCl_3$ *plus drop of* CD_3OD *at 500 MHz.*

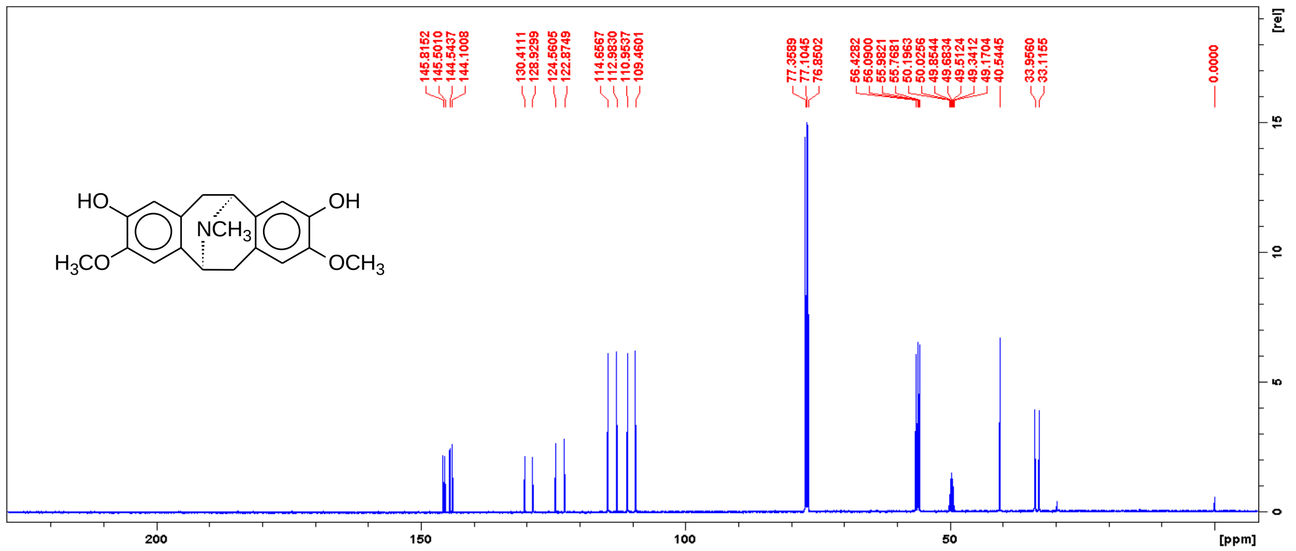

Figure S77. *^{13}C NMR spectrum of* ***bisnorargemonine*** *(**9**) in $CDCl_3$ plus drop of CD_3OD at 125 MHz.*

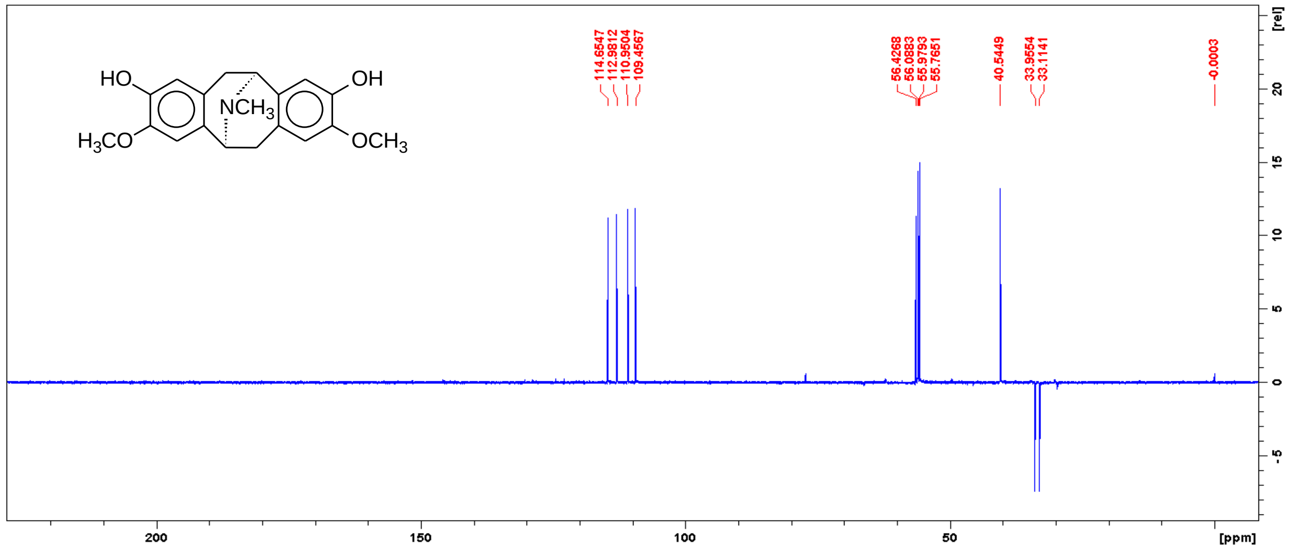

Figure S78. *DEPT 135 spectrum of* ***bisnorargemonine*** *(**9**) in $CDCl_3$ plus drop of CD_3OD at 125 MHz.*

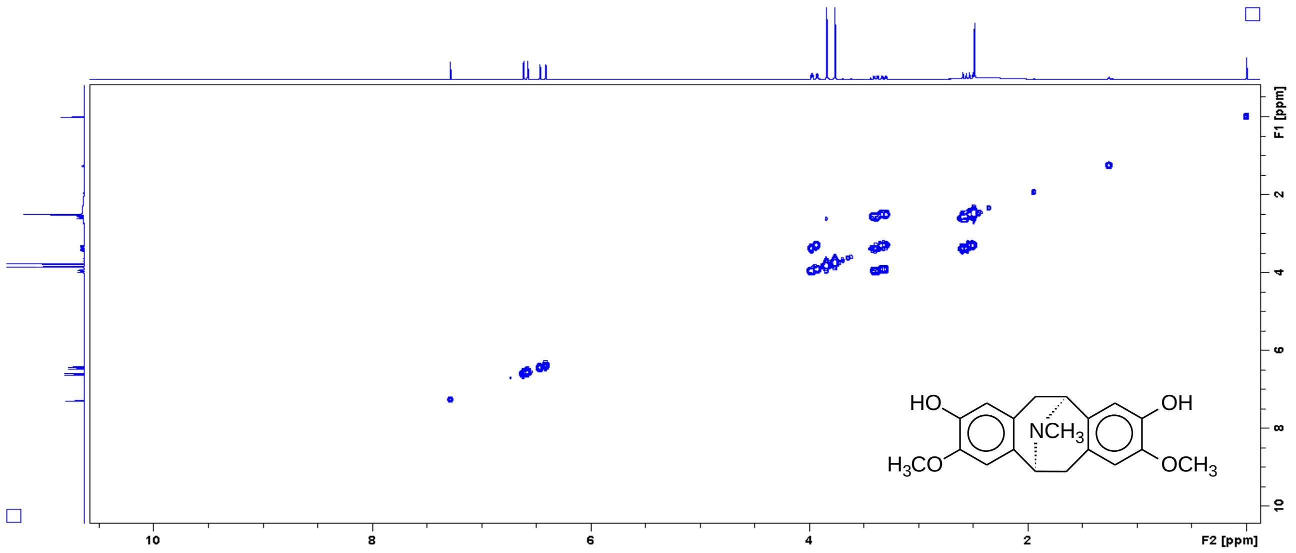

Figure S79. *^1H-^1H correlation map from COSY NMR experiment of* ***bisnorargemonine*** *(**9**) in $CDCl_3$ plus drop of CD_3OD at 500 MHz.*

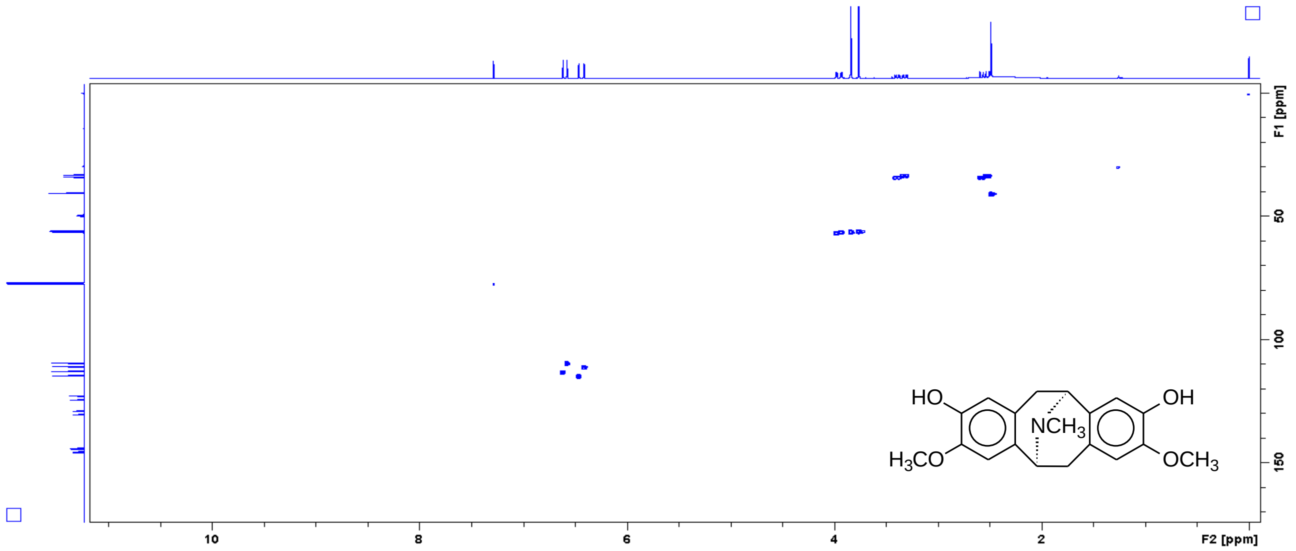

Figure S80. *One-bond* ^{1}H-^{13}C *correlation map from HSQC NMR experiment of* ***bisnorargemonine*** *(****9****) in* $CDCl_3$ *plus drop of* CD_3OD *at 500 (*^{1}H*) and 125 MHz (*^{13}C*), respectively.*

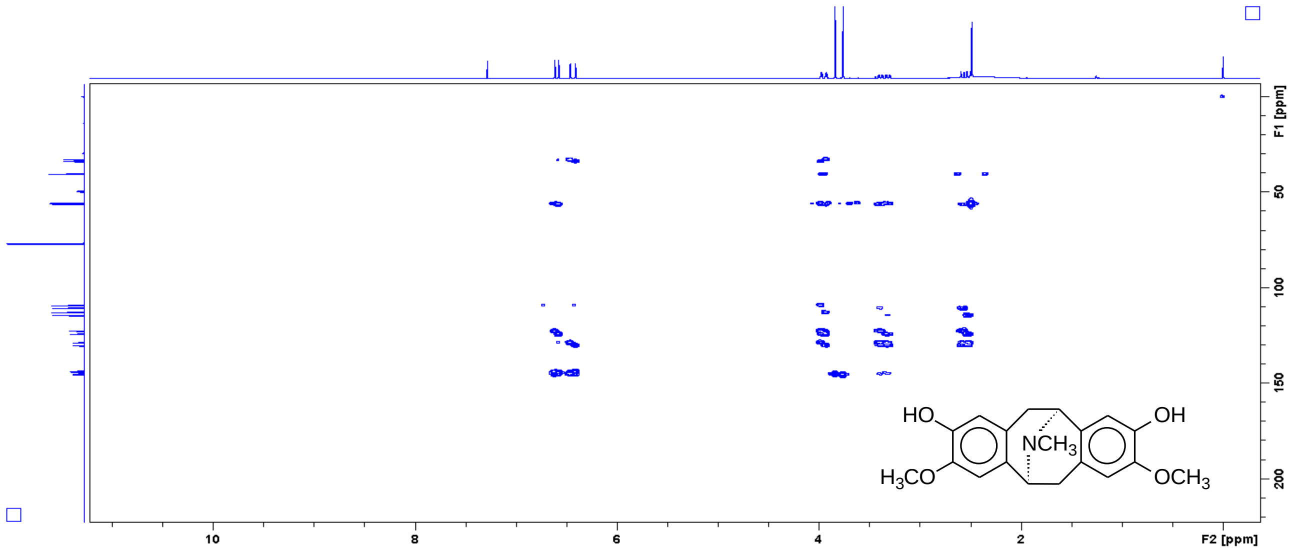

Figure S81. *Long-range* ^{1}H-^{13}C *correlation map from HMBC NMR experiment of* ***bisnorargemonine*** *(****9****) in* $CDCl_3$ *plus drop of* CD_3OD *at 500 (*^{1}H*) and 125 MHz (*^{13}C*), respectively.*

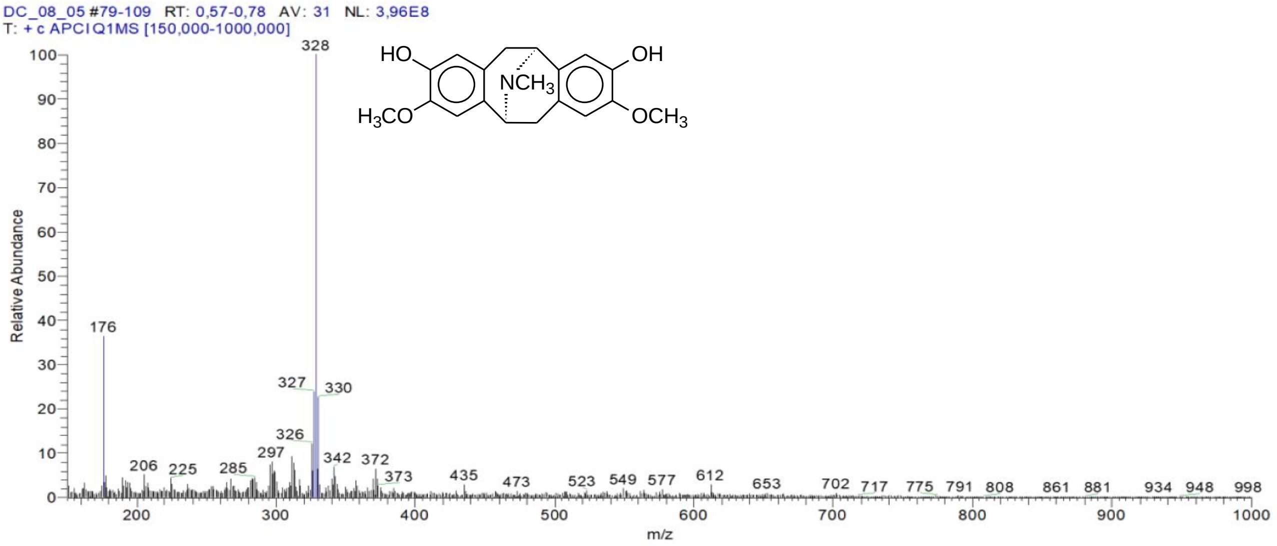

Figure S82. *LR-APCI(+)-MS spectrum of* ***bisnorargemonine*** *(****9****) (m/z 328* $[M+H]^{+}$*).*

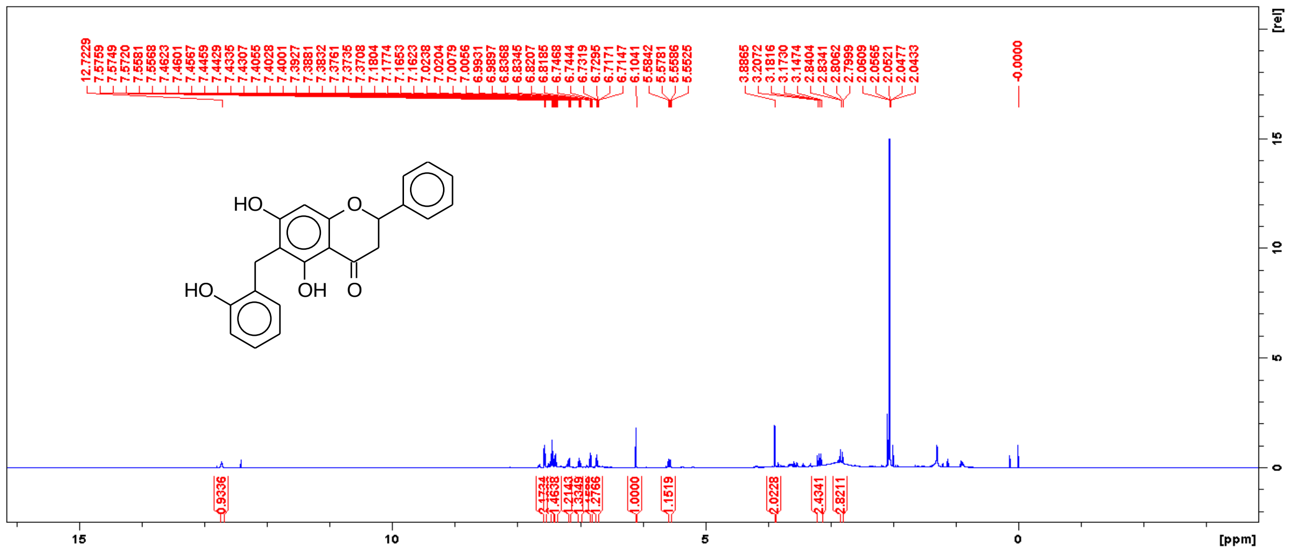

Figure S83. *^{1}H NMR spectrum of **isochamanetin (10)** in acetone-d6 at 500 MHz.*

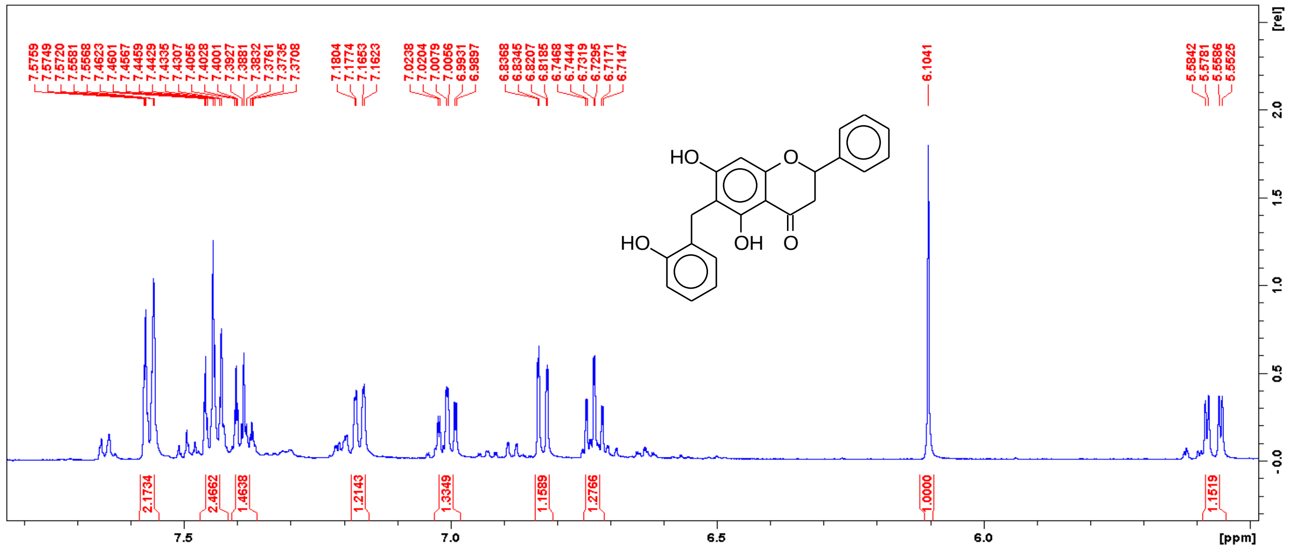

Figure S84. *Expansion (δ 7.82 – 7.49) of the ^{1}H NMR spectrum of **isochamanetin (10)** in acetone-d6 at 500 MHz.*

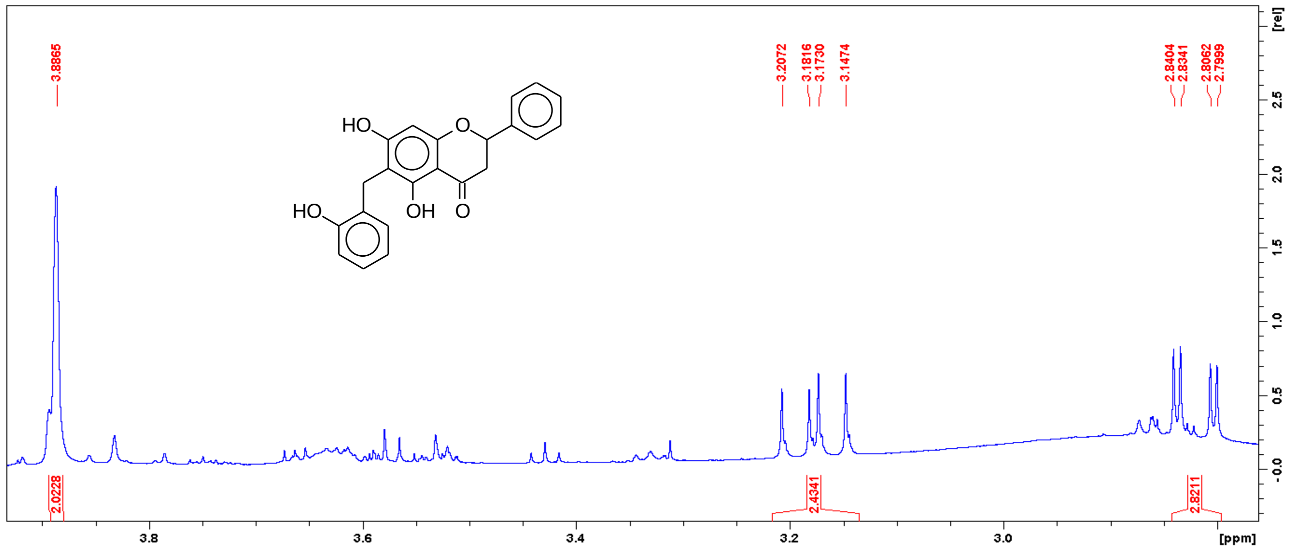

Figure S85. *Expansion (δ 3.92 – 2.76) of the ^{1}H NMR spectrum of **isochamanetin (10)** in acetone-d6 at 500 MHz.*

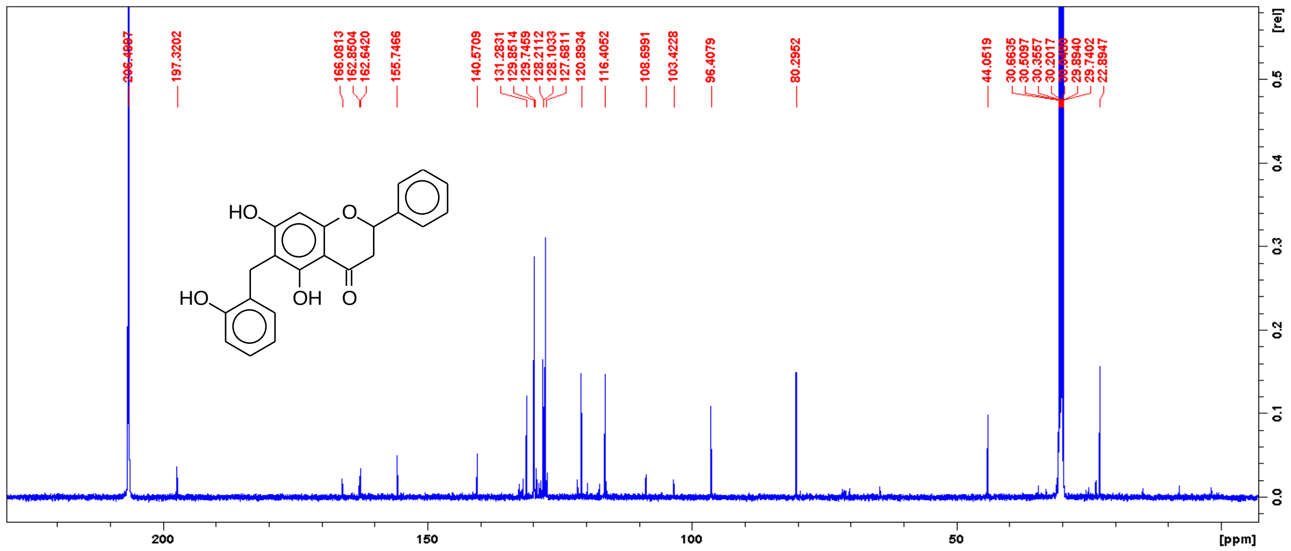

Figure S86. *^{13}C NMR spectrum of **isochamanetin (10)** in acetone-d_6 at 125 MHz.*

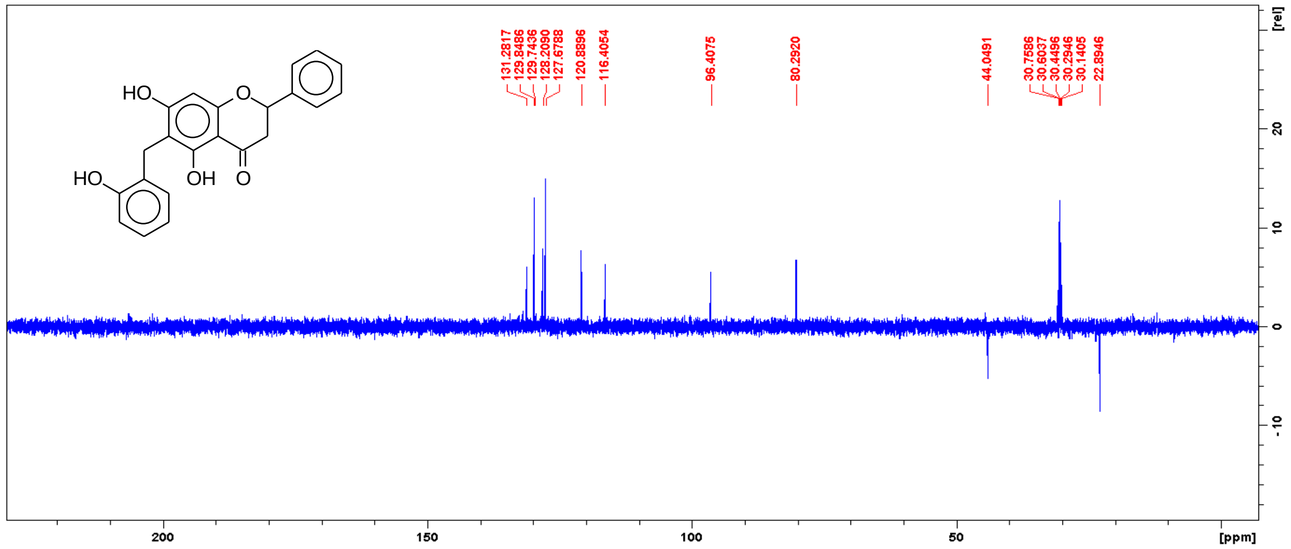

Figure S87. *DEPT 135 spectrum of **isochamanetin (10)** in acetone-d_6 at 125 MHz.*

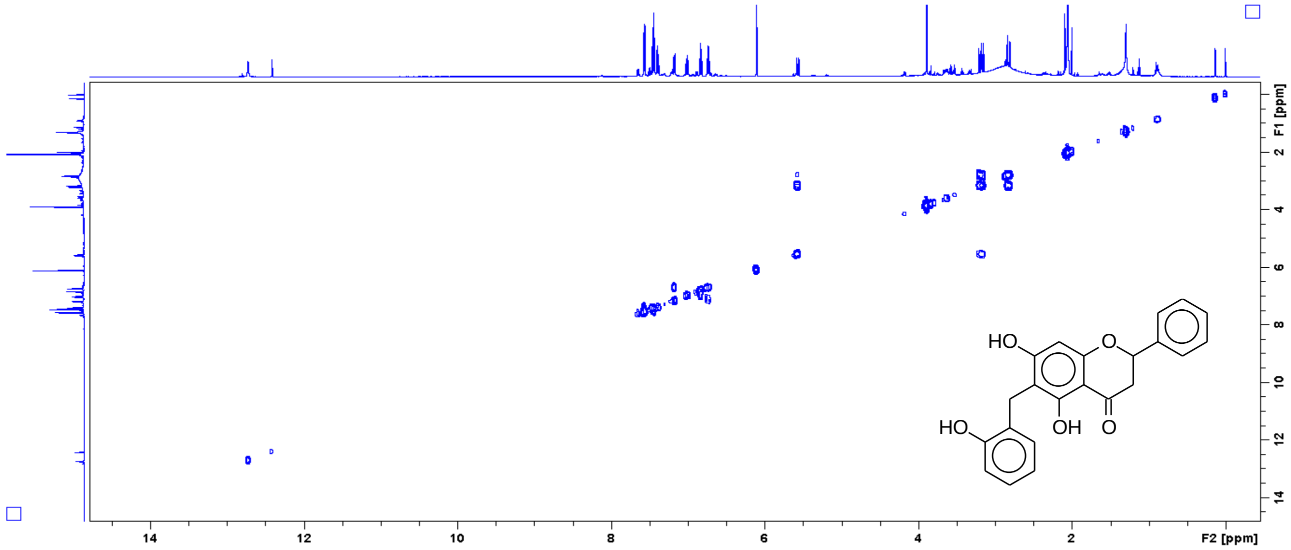

Figure S88. *^{1}H-^{1}H correlation map from COSY NMR experiment of **isochamanetin (10)** in acetone-d_6 at 500 MHz.*

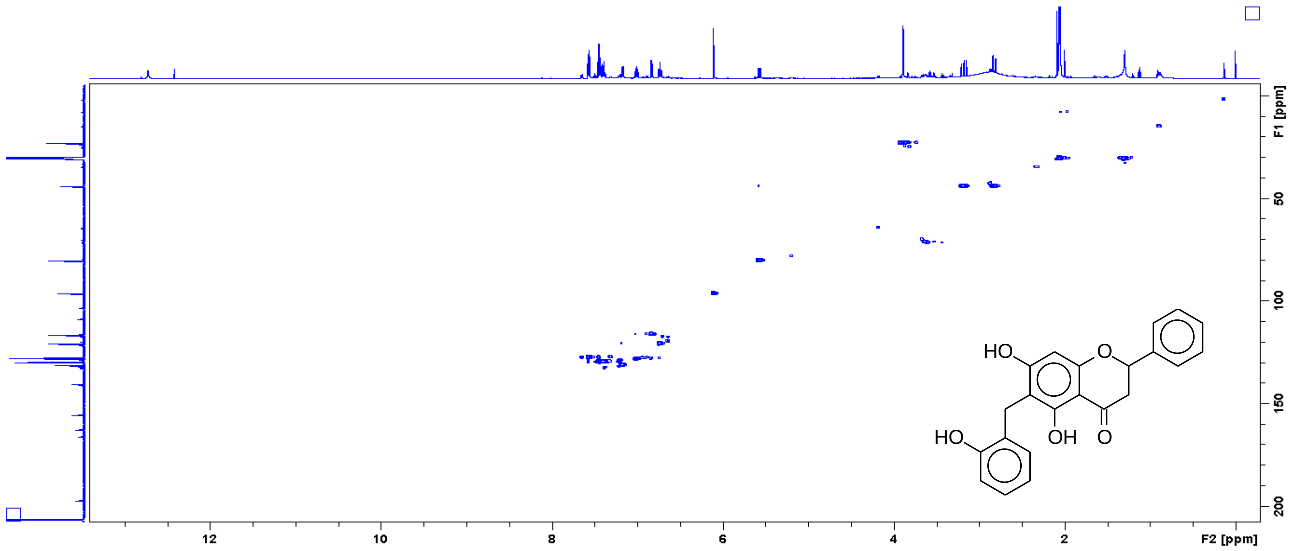

Figure S89. *One-bond* [1]*H-*[13]*C correlation map from HSQC NMR experiment of* ***isochamanetin (10)*** *in acetone-d*$_6$ *at 500 (*[1]*H) and 125 MHz (*[13]*C), respectively.*

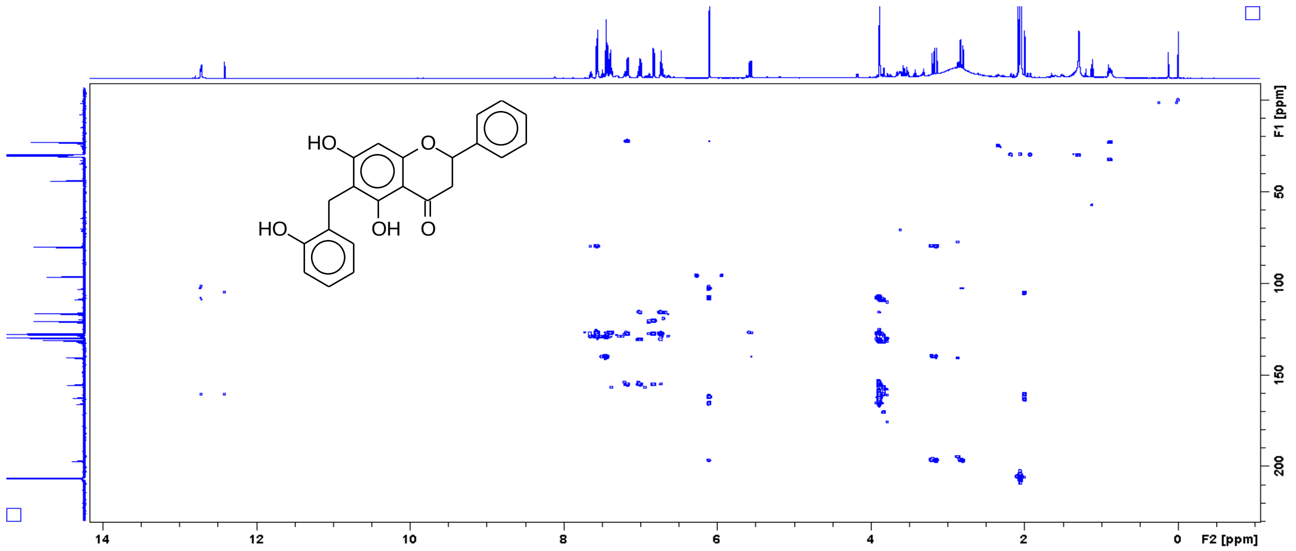

Figure S90. *Long-range* [1]*H-*[13]*C correlation map from HMBC NMR experiment of* ***isochamanetin (10)*** *in acetone-d*$_6$ *at 500 (*[1]*H) and 125 MHz (*[13]*C), respectively.*

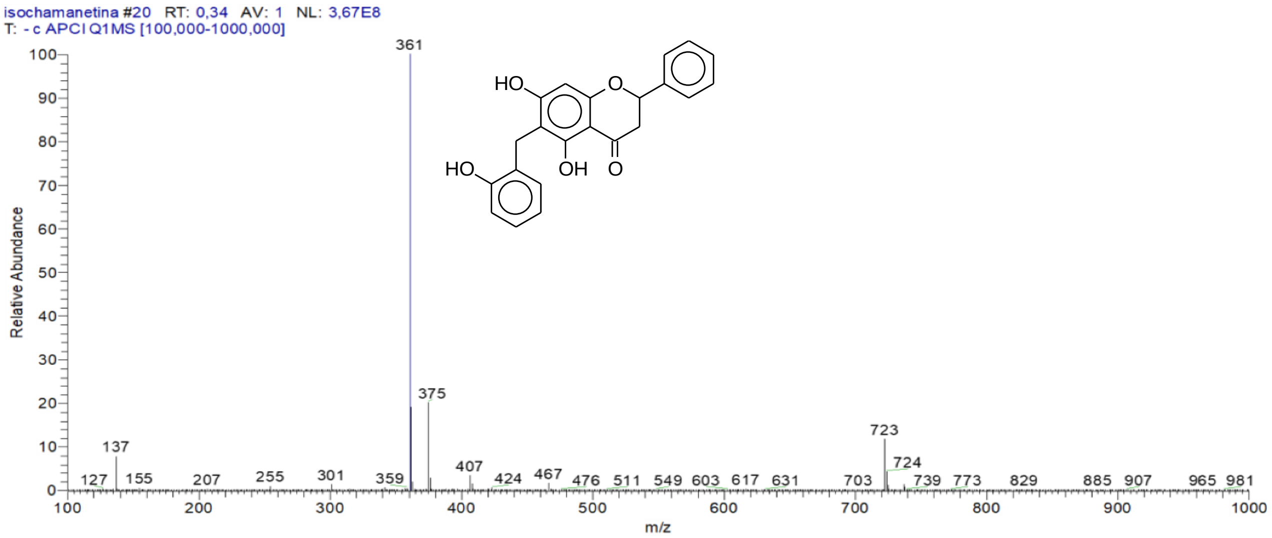

Figure S91. *LR-APCI(–)-MS spectrum of* ***isochamanetin (10)*** *(m/z 361 [M–H]*$^-$*).*

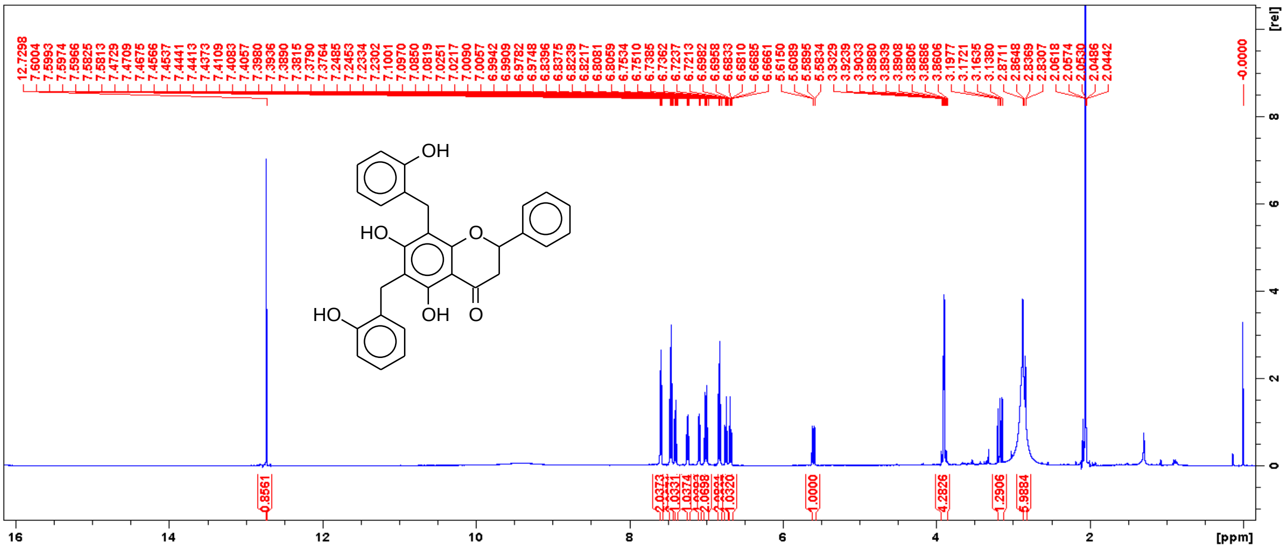

Figure S92. *¹H NMR spectrum of* ***dichamanetin (11)*** *in acetone-d$_6$ at 500 MHz.*

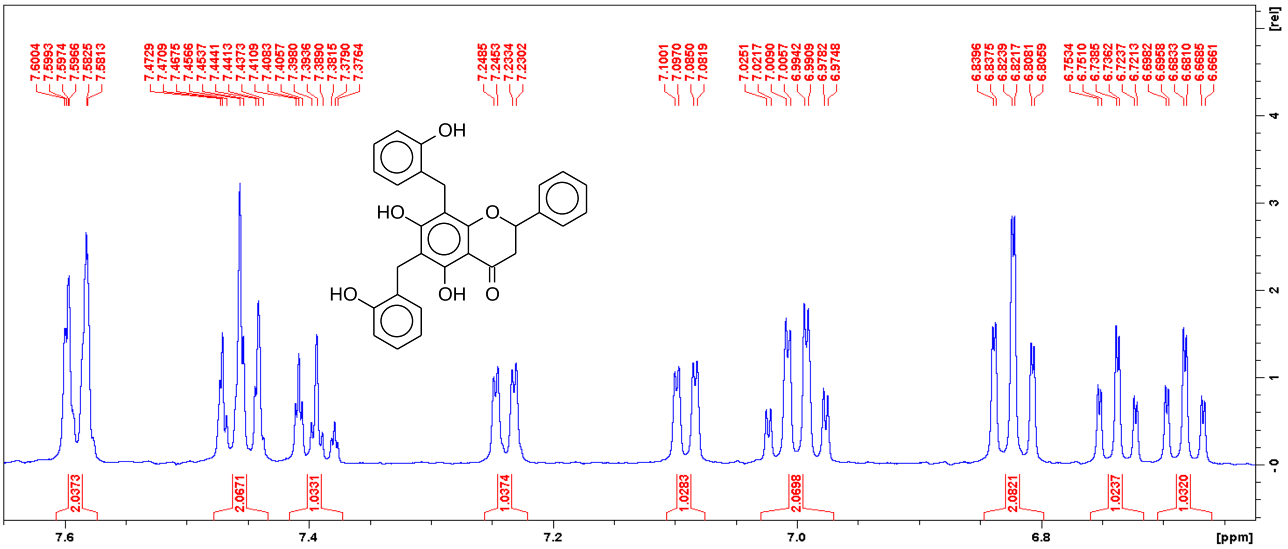

Figure S93. Expansion (*δ* 7.64 – 6.63) *of the ¹H NMR spectrum of* ***dichamanetin (11)*** *in acetone-d$_6$ at 500 MHz.*

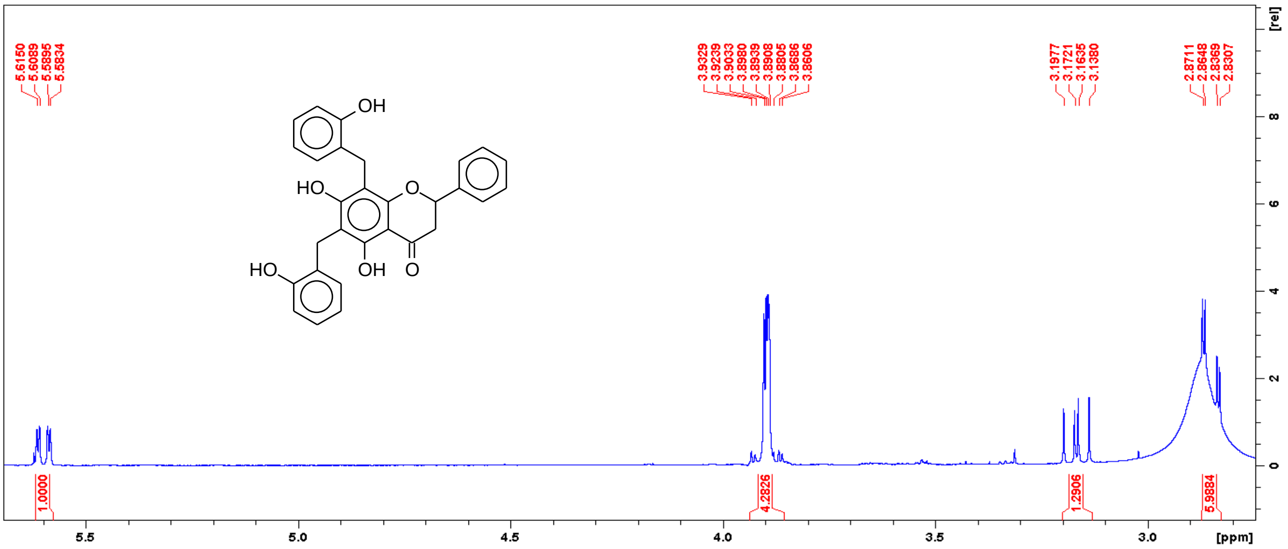

Figure S94. Expansion (*δ* 5.68 – 2.75) *of the ¹H NMR spectrum of* ***dichamanetin (11)****in acetone-d$_6$ at 500 MHz.*

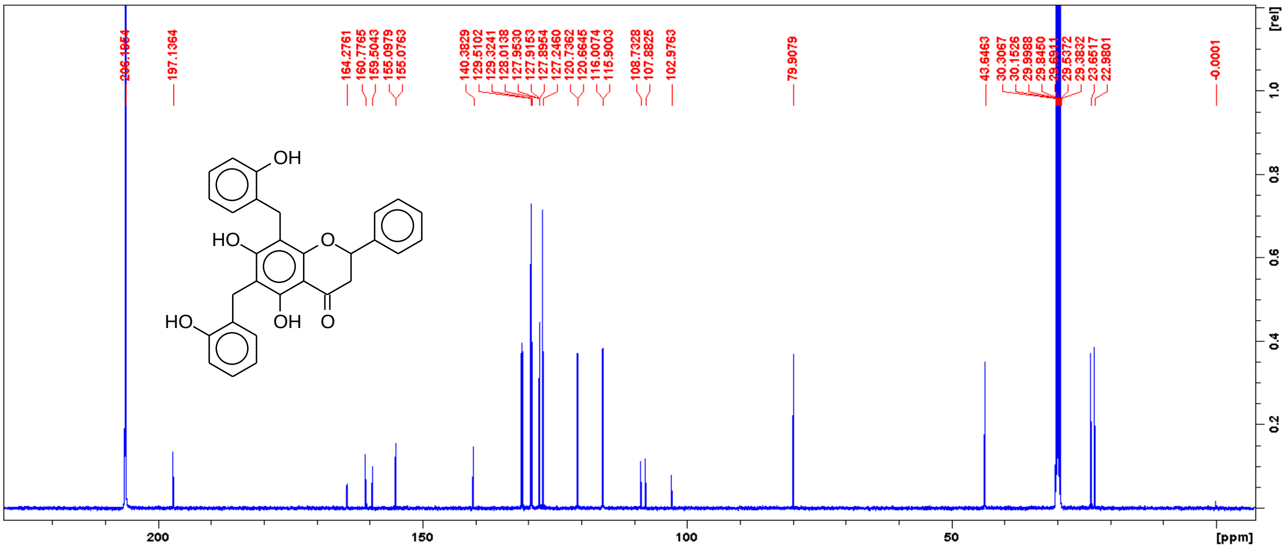

Figure S95. ^{13}*C NMR spectrum of* ***dichamanetin (11)*** *in acetone-d$_6$ at 125 MHz.*

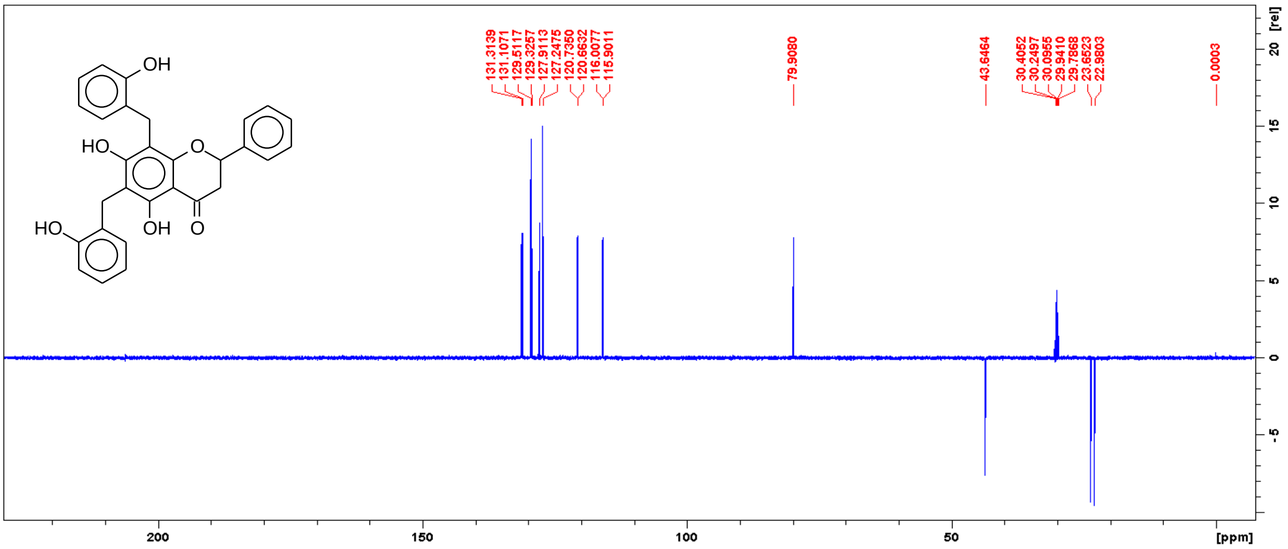

Figure S96. *DEPT 135 spectrum of* ***dichamanetin (11)*** *in acetone-d$_6$ at 125 MHz.*

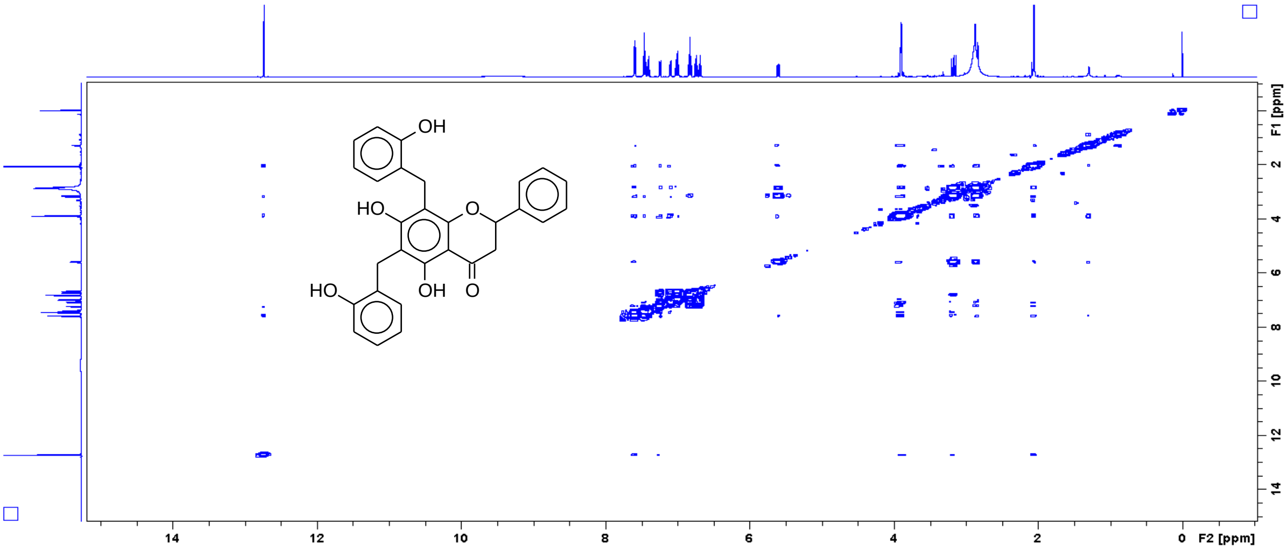

Figure S97. ^{1}*H-*^{1}*H correlation map from COSY NMR experiment of* ***dichamanetin (11)*** *in acetone-d$_6$ at 500 MHz.*

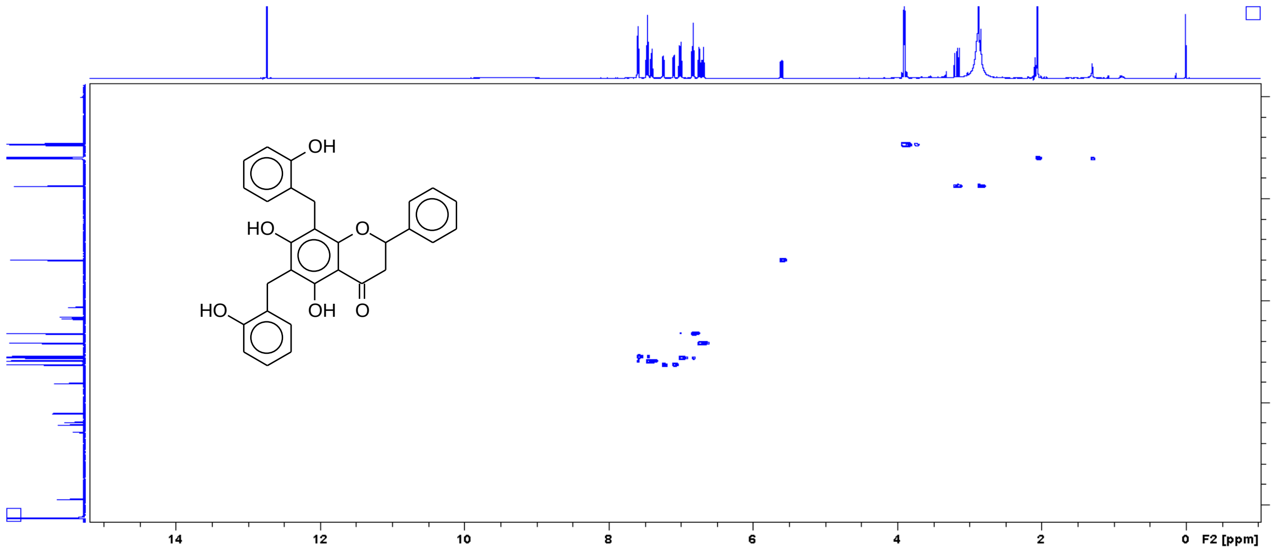

Figure S98. *One-bond ^{1}H-^{13}C correlation map from HSQC NMR experiment of* ***dichamanetin (11)*** *in acetone-d_6 at 500 (^{1}H) and 125 MHz (^{13}C), respectively.*

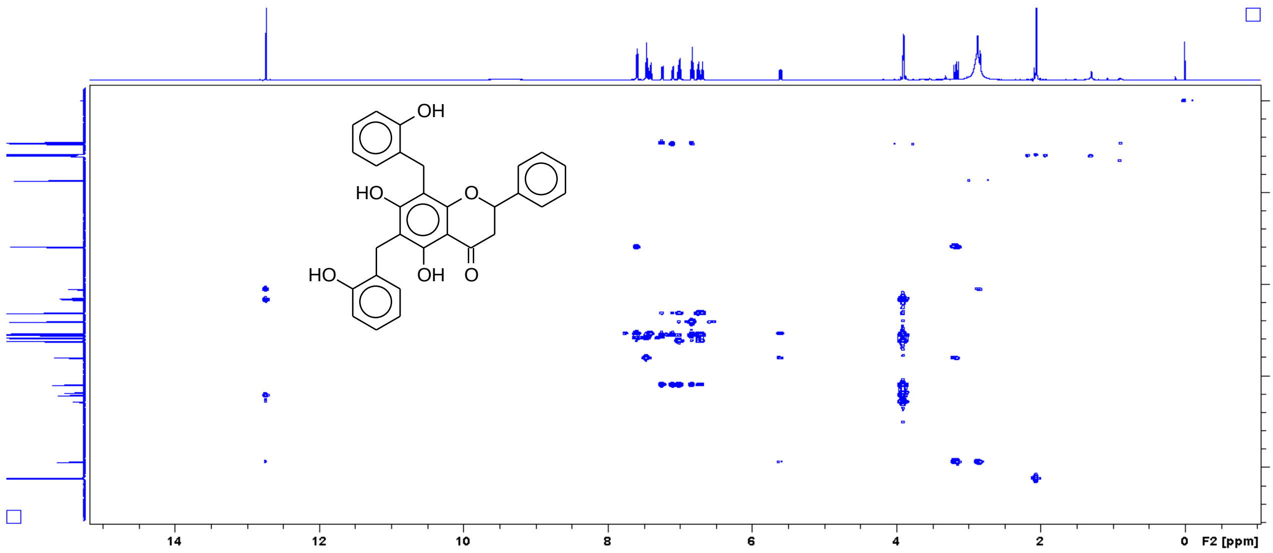

Figure S99. *Long-range ^{1}H-^{13}C correlation map from HMBC NMR experiment of* ***dichamanetin (11)*** *in acetone-d_6 at 500 (^{1}H) and 125 MHz (^{13}C), respectively.*

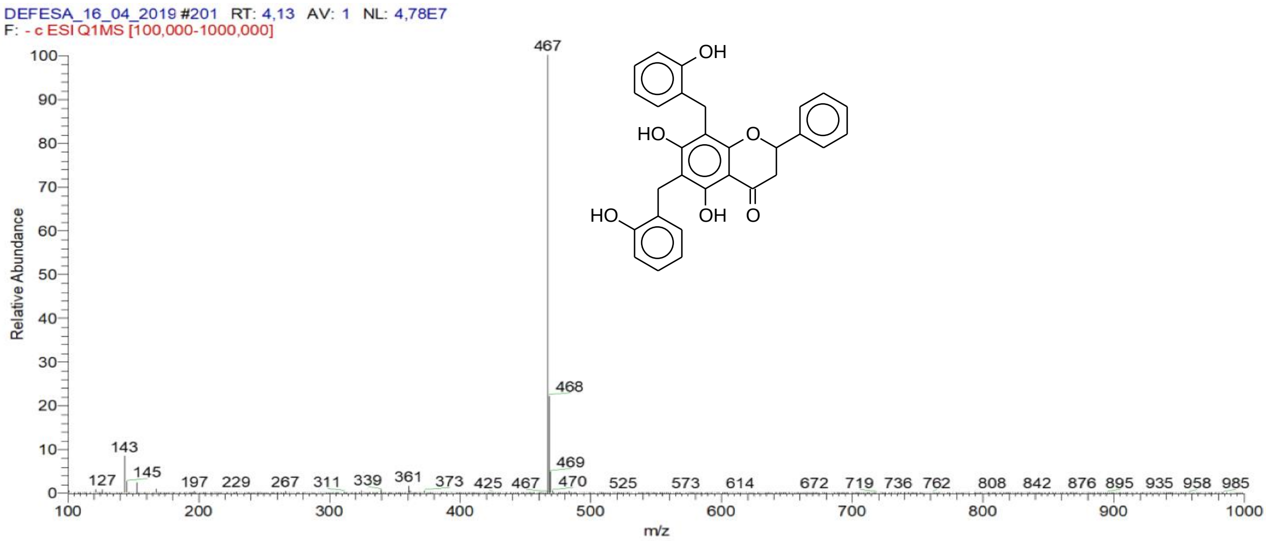

Figure S100. *LR-ESI(–)-MS spectrum of* ***dichamanetin (11)*** *(m/z 467 [M–H]⁻).*

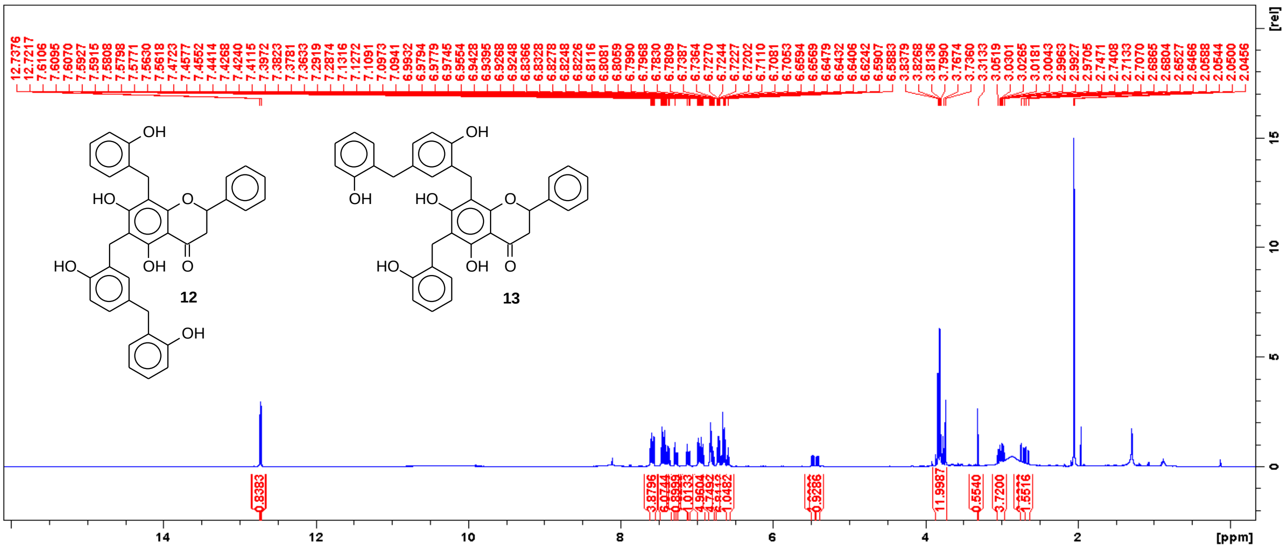

Figure S101. ^{1}H NMR spectrum of the mixture of **uvarinol (12)** and **isouvarinol (13)** in acetone-d_6 at 500 MHz.

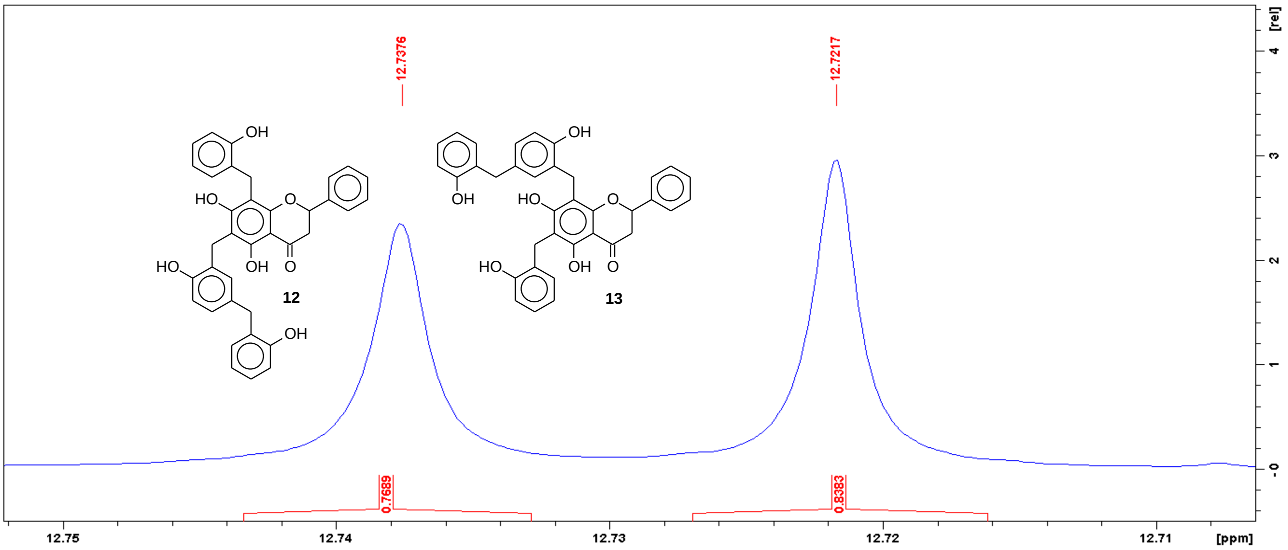

Figure S102. Expansion (δ 12.75 – 12.70) of the ^{1}H NMR spectrum of the mixture of **uvarinol (12)** and **isouvarinol (13)** in acetone-d_6 at 500 MHz.

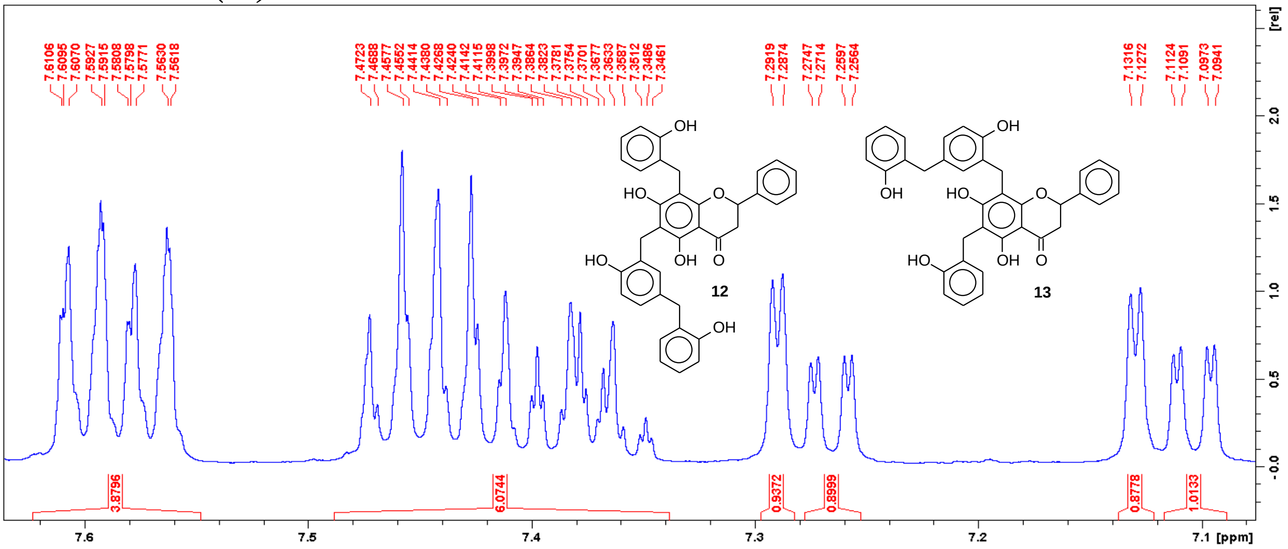

Figure S103. Expansion (δ 7.63 – 7.07) of the ^{1}H NMR spectrum of the mixture of **uvarinol (12)** and **isouvarinol (13)** in acetone-d_6 at 500 MHz.

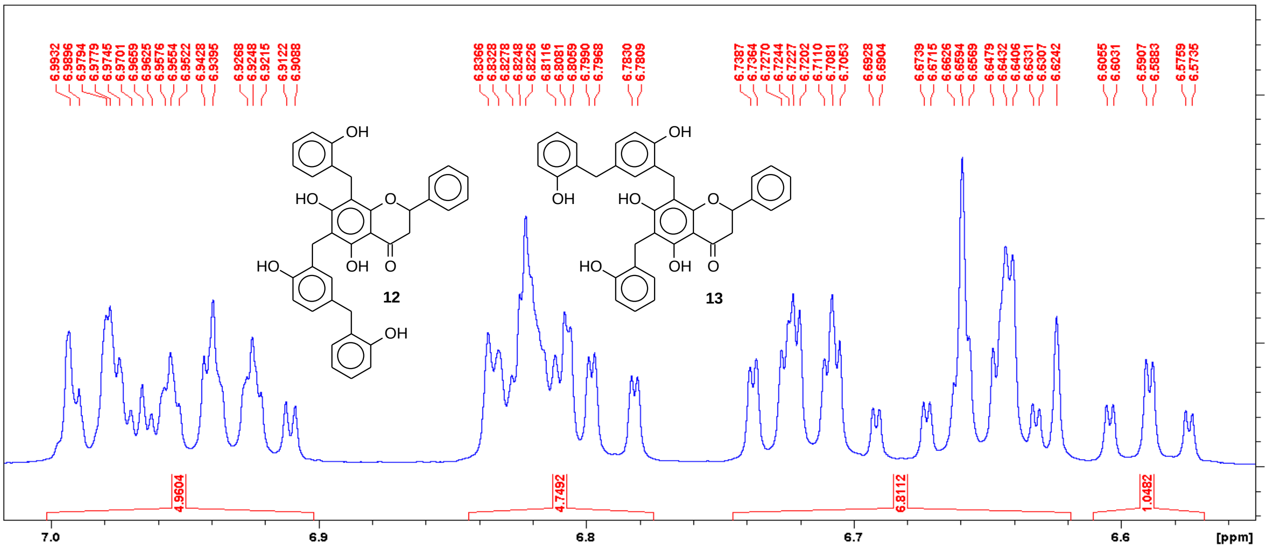

Figure S104. *Expansion (δ 7.01 – 6.55) of the* [1]*H NMR spectrum of the mixture of* ***uvarinol*** *(**12**) and* ***isouvarinol*** *(**13**) in acetone-d*$_6$ *at 500 MHz.*

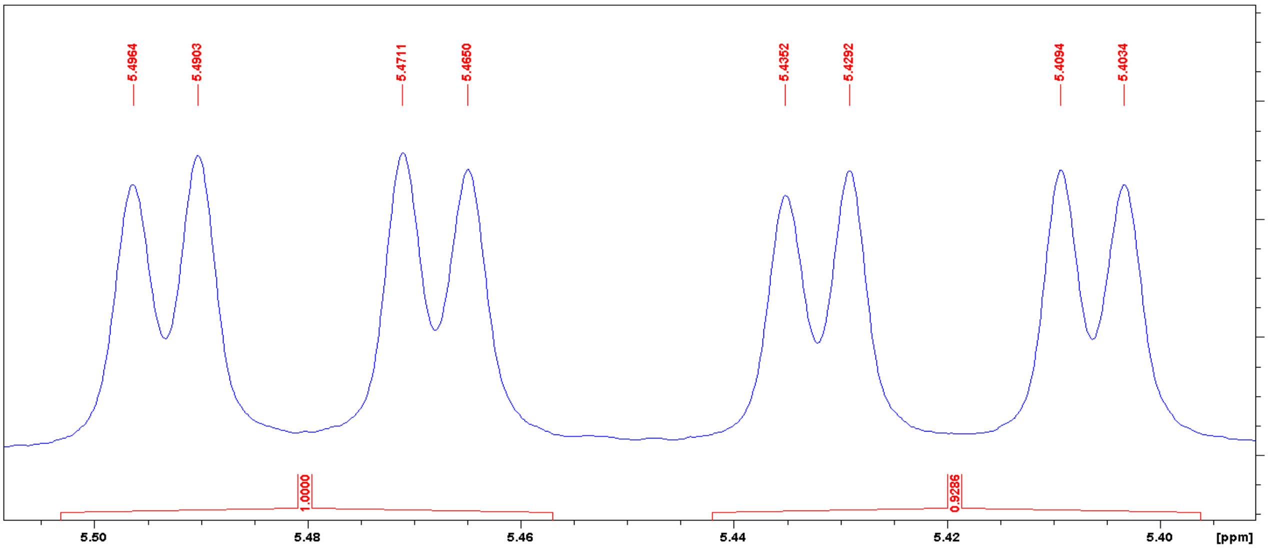

Figure S105. *Expansion (δ 5.50 – 5.39) of the* [1]*H NMR spectrum of the mixture of* ***uvarinol*** *(**12**) and* ***isouvarinol*** *(**13**) in acetone-d*$_6$ *at 500 MHz.*

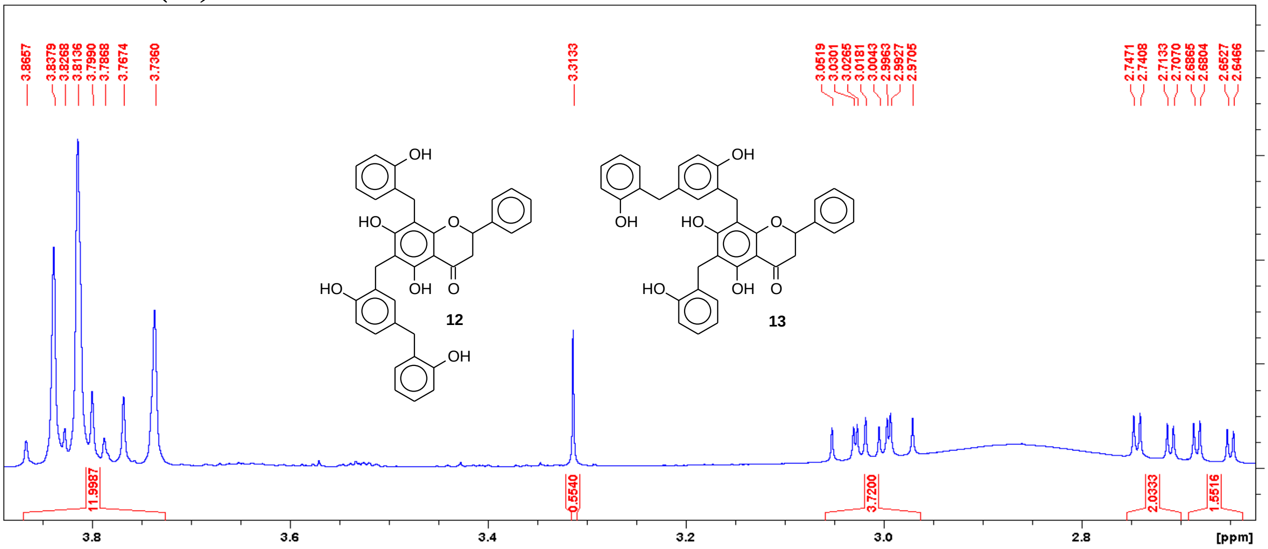

Figure S106. *Expansion (δ 5.50 – 5.39) of the* [1]*H NMR spectrum of the mixture of* ***uvarinol*** *(**12**) and* ***isouvarinol*** *(**13**) in acetone-d*$_6$ *at 500 MHz.*

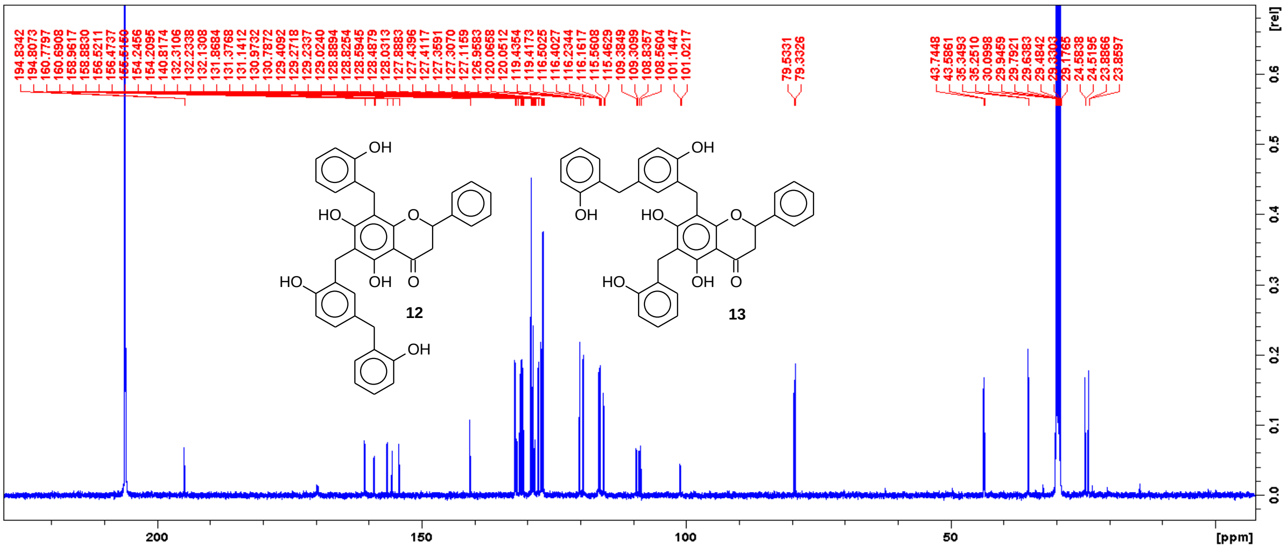

Figure S107. *^{13}C NMR spectrum of the mixture of* ***uvarinol (12)*** *and* ***isouvarinol (13)*** *in acetone-d_6 at 125 MHz.*

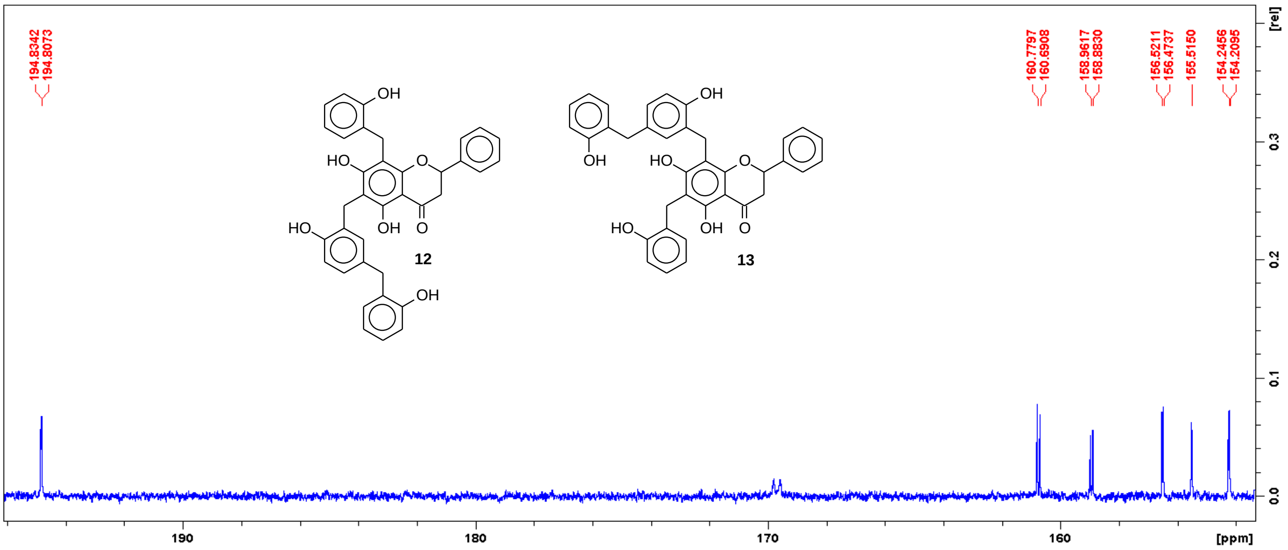

Figure S108. *Expansion (δ 196.0 – 153.4) of the ^{13}C NMR spectrum of the mixture of* ***uvarinol (12)*** *and* ***isouvarinol (13)*** *in acetone-d_6 at 125 MHz.*

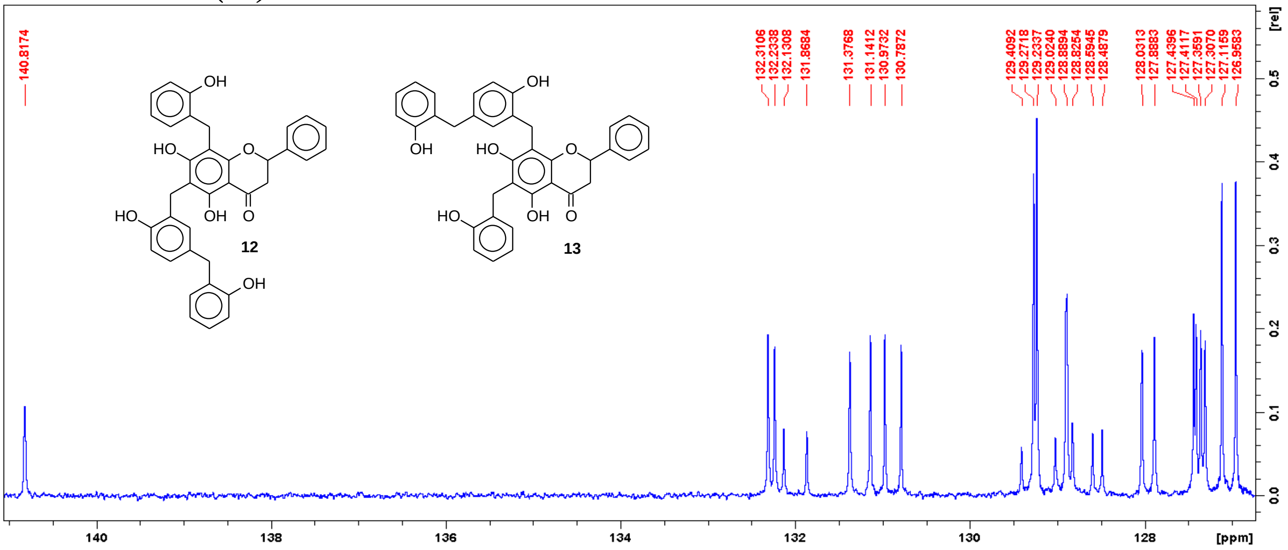

Figure S109. *Expansion (δ 141.0 – 126.7) of the ^{13}C NMR spectrum of the mixture of* ***uvarinol (12)*** *and* ***isouvarinol (13)*** *in acetone-d_6 at 125 MHz.*

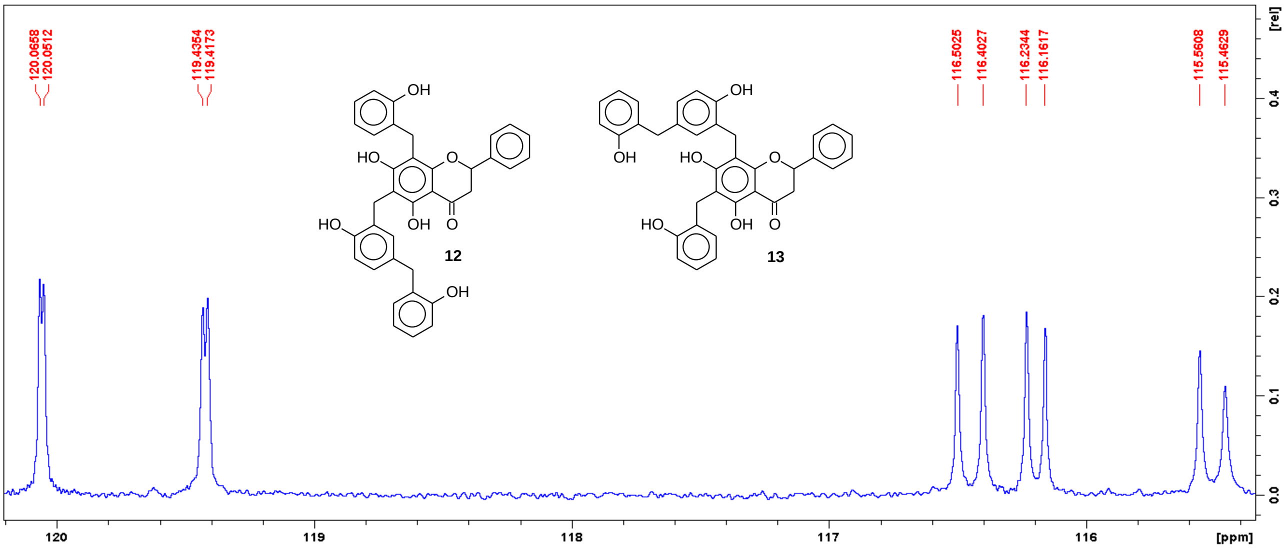

Figure S110. *Expansion (δ 120.1 – 115.3) of the* ^{13}*C NMR spectrum of the mixture of* ***uvarinol (12)*** *and* ***isouvarinol (13)*** *in acetone-d*$_6$ *at 125 MHz.*

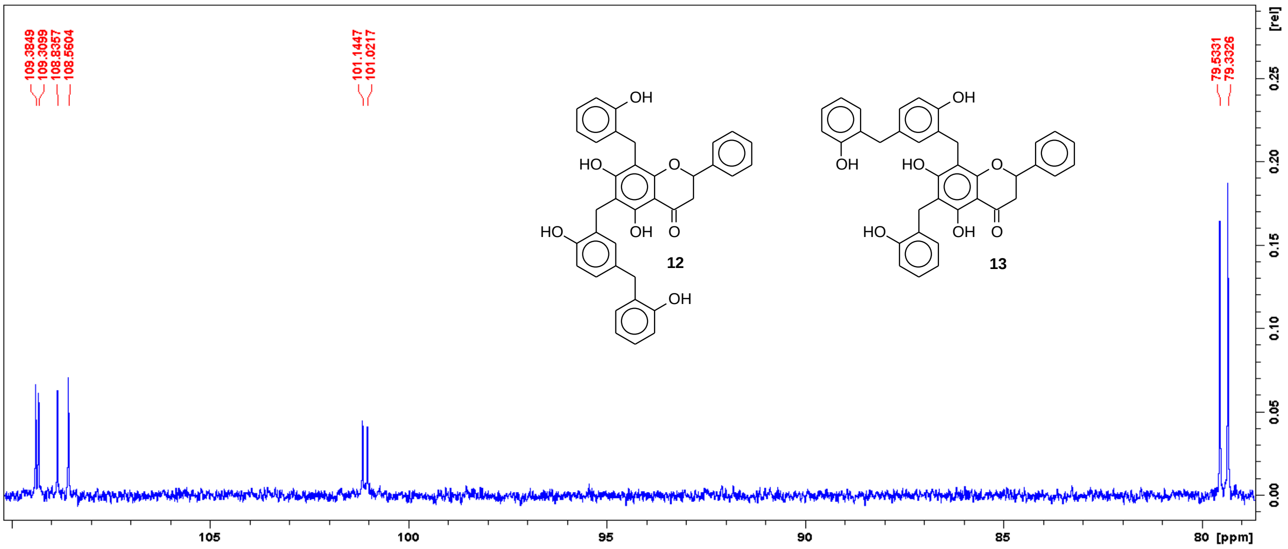

Figure S111. *Expansion (δ 110.1 – 78.7) of the* ^{13}*C NMR spectrum of the mixture of* ***uvarinol (12)*** *and* ***isouvarinol (13)*** *in acetone-d*$_6$ *at 125 MHz.*

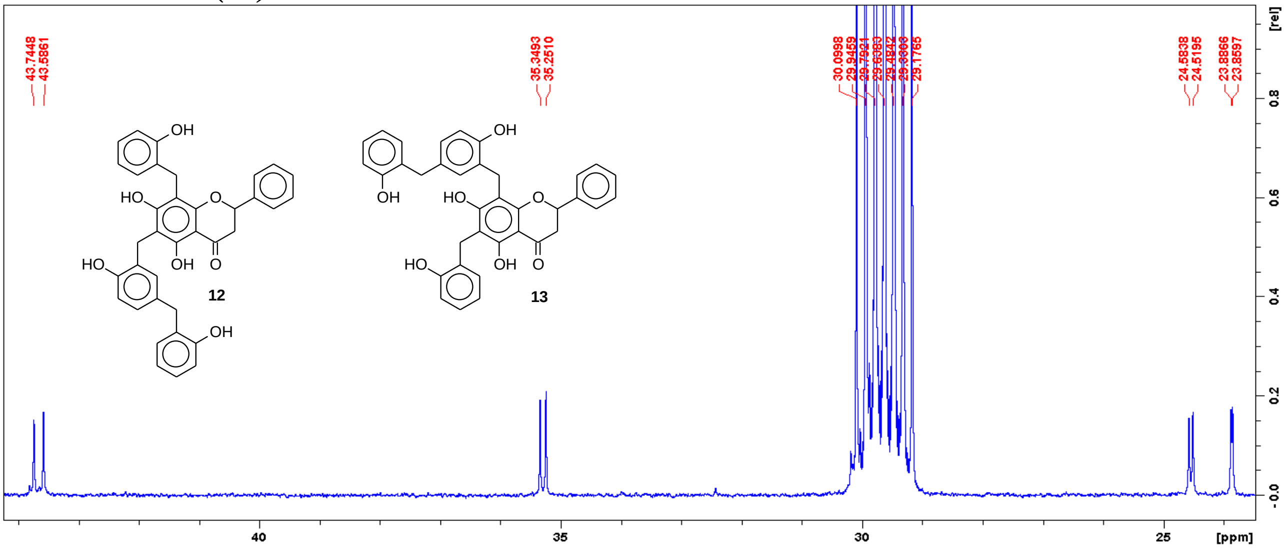

Figure S112. *Expansion (δ 44.2 –23.5) of the* ^{13}*C NMR spectrum of the mixture of* ***uvarinol (12)*** *and* ***isouvarinol (13)*** *in acetone-d*$_6$ *at 125 MHz.*

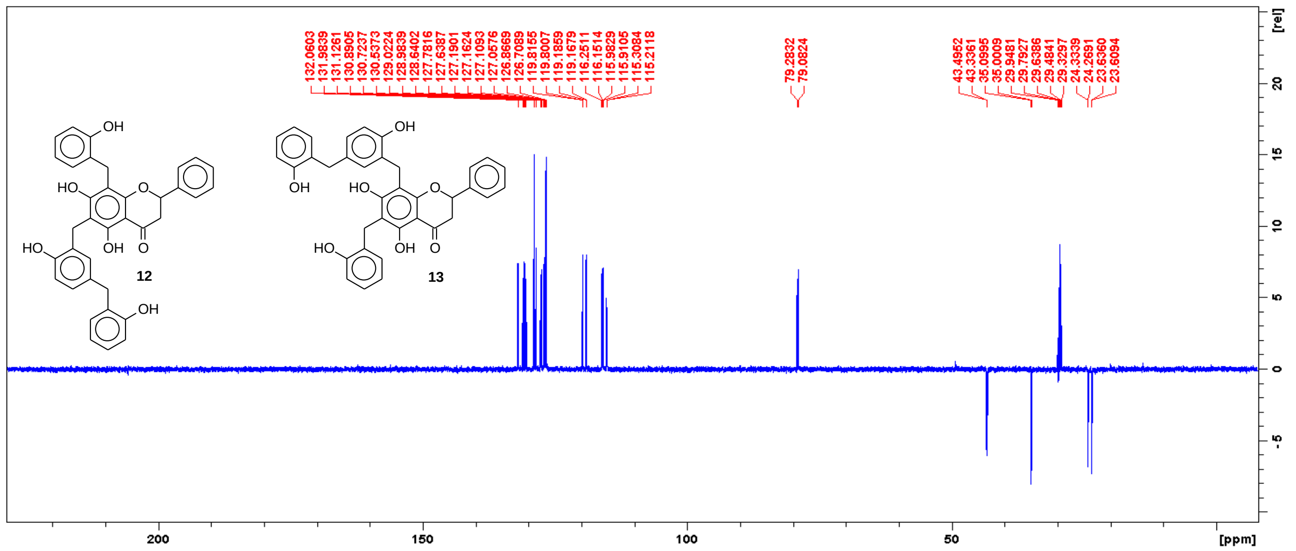

Figure S113. *DEPT 135 spectrum of the mixture of* ***uvarinol*** ***(12)*** *and* ***isouvarinol*** ***(13)*** *in acetone-d_6 at 125 MHz.*

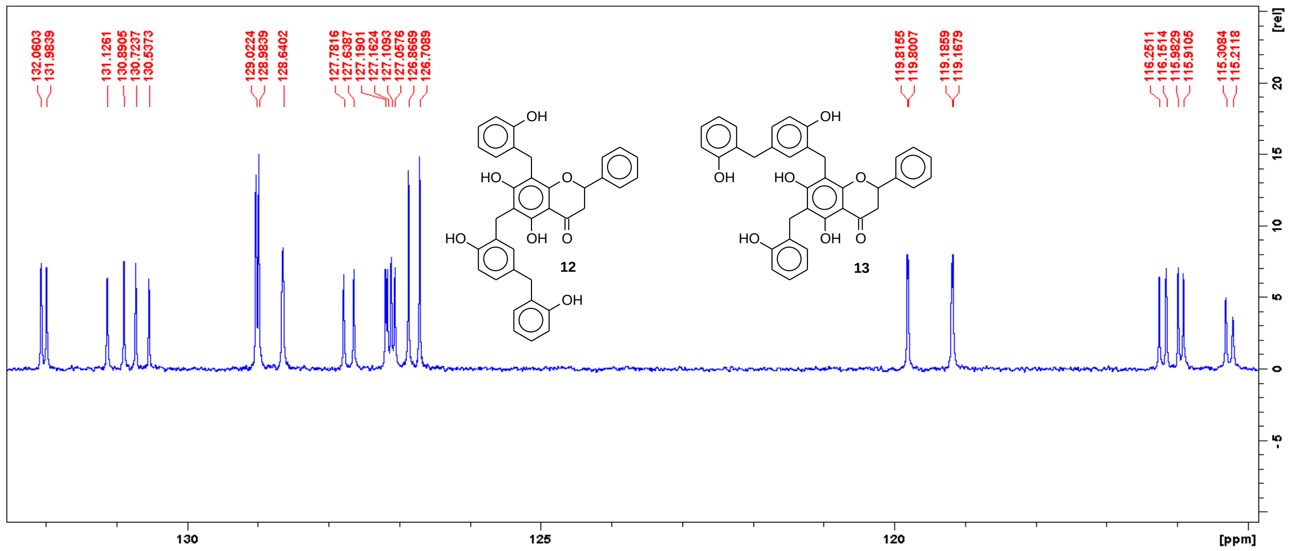

Figure S114. *Expansion (δ 132.4 – 114.9) of the DEPT 135 spectrum of the mixture of* ***uvarinol*** ***(12)*** *and* ***isouvarinol*** ***(13)*** *in acetone-d_6 at 125 MHz.*

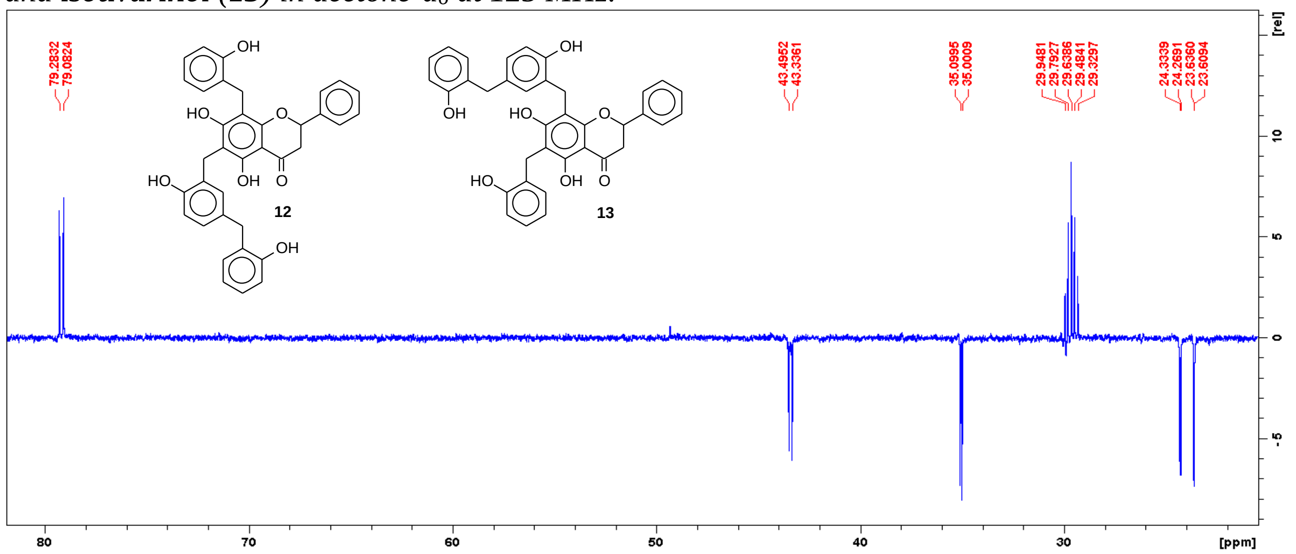

Figure S115. *Expansion (δ 81.7 – 20.7) of the DEPT 135 spectrum of the mixture of* ***uvarinol*** ***(12)*** *and* ***isouvarinol*** ***(13)*** *in acetone-d_6 at 125 MHz.*

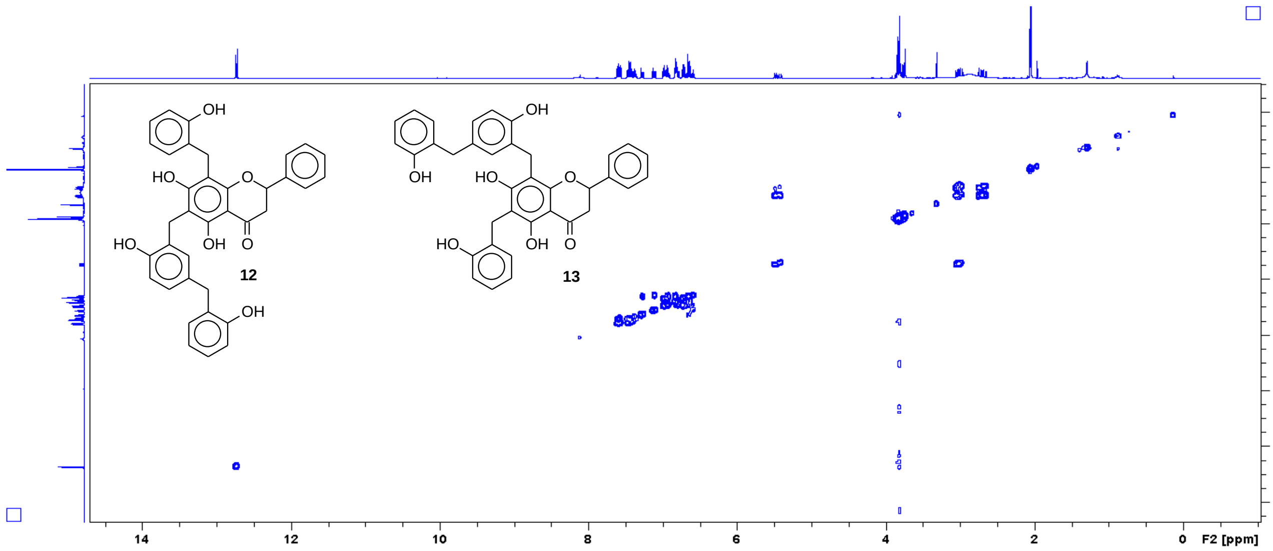

Figure S116. *^{1}H-^{1}H correlation map from COSY NMR experiment of the mixture of* ***uvarinol*** *(12) and* ***isouvarinol*** *(13) in acetone-d$_6$ at 500 MHz.*

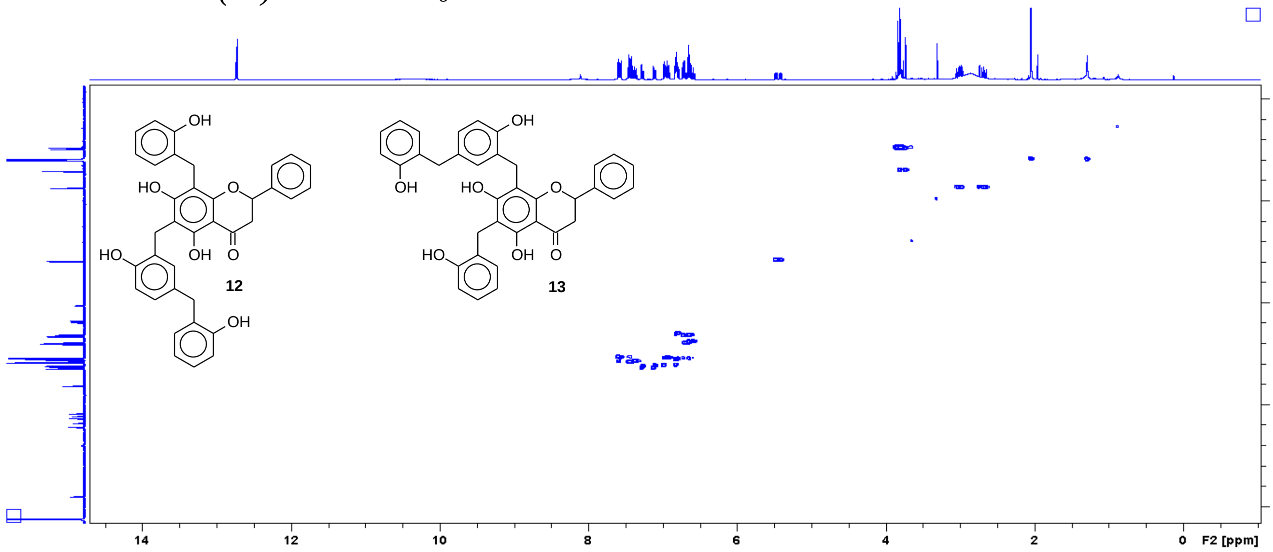

Figure S117. *One-bond ^{1}H-^{13}C correlation map from HSQC NMR experiment of the mixture of* ***uvarinol*** *(12) and* ***isouvarinol*** *(13) in acetone-d$_6$ at 500 (^{1}H) and 125 MHz (^{13}C), respectively.*

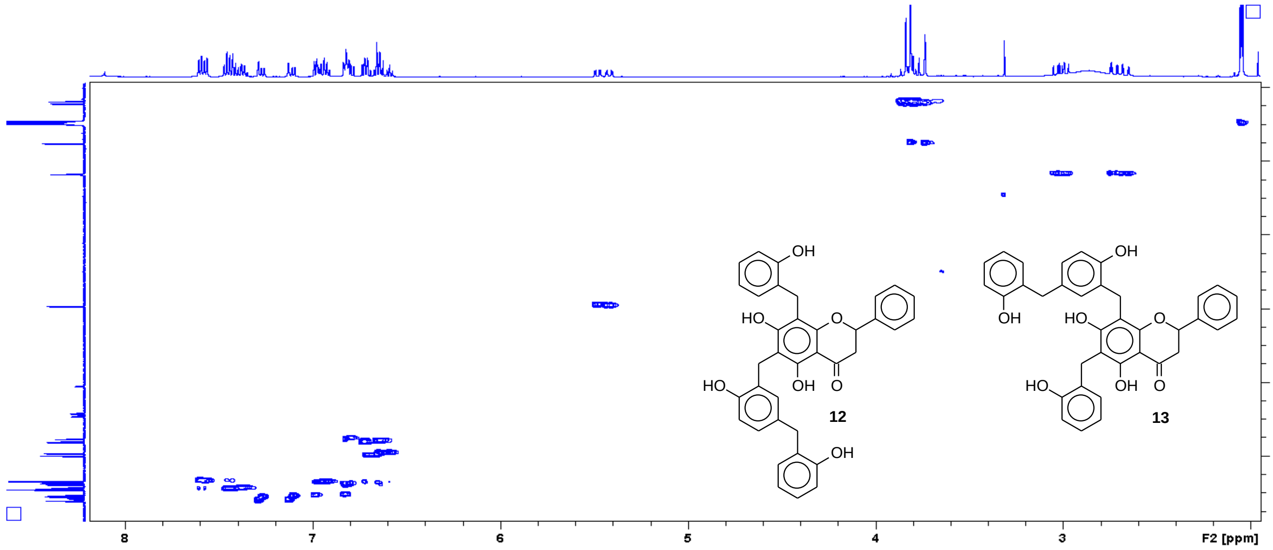

Figure S118. *Expansion of the one-bond ^{1}H-^{13}C correlation map from HSQC NMR experiment of the mixture of* ***uvarinol*** *(12) and* ***isouvarinol*** *(13) in acetone-d$_6$ at 500 (^{1}H) and 125 MHz (^{13}C), respectively.*

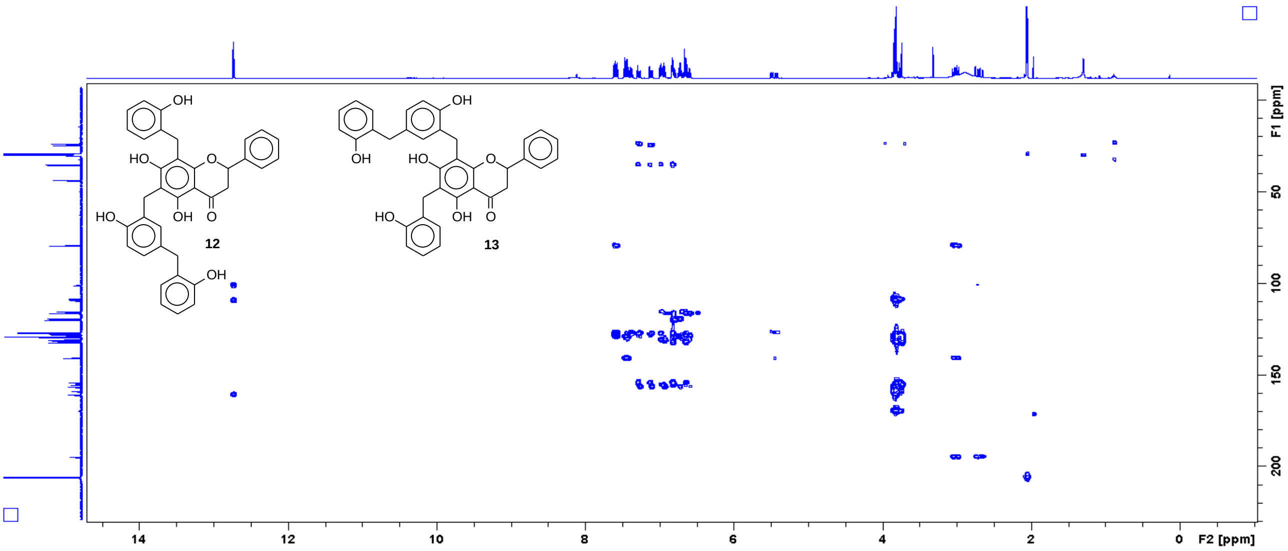

Figure S119. *Long-range ^{1}H-^{13}C correlation map from HMBC NMR experiment of the mixture of* ***uvarinol (12)*** *and* ***isouvarinol (13)*** *in acetone-d$_6$ at 500 (^{1}H) and 125 MHz (^{13}C), respectively.*

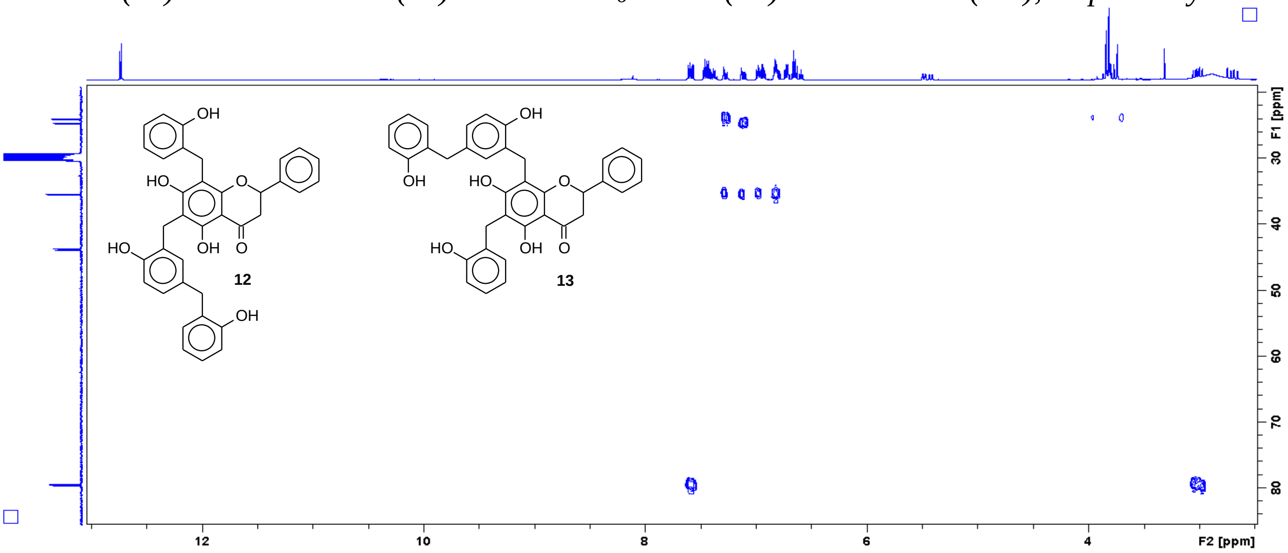

Figure S120. *Expansion of the long-range ^{1}H-^{13}C correlation map from HMBC NMR experiment of the mixture of* ***uvarinol (12)*** *and* ***isouvarinol (13)*** *in acetone-d$_6$ at 500 (^{1}H) and 125 MHz (^{13}C), respectively.*

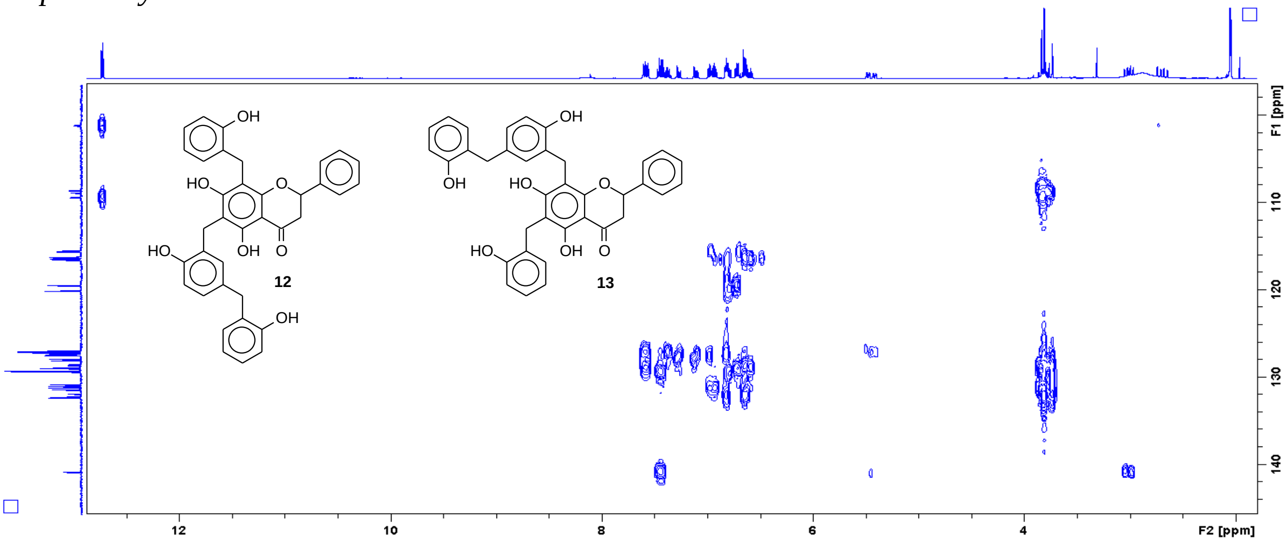

Figure S121. *Expansion of the long-range ^{1}H-^{13}C correlation map from HMBC NMR experiment of the mixture of* ***uvarinol (12)*** *and* ***isouvarinol (13)*** *in acetone-d$_6$ at 500 (^{1}H) and 125 MHz (^{13}C), respectively.*

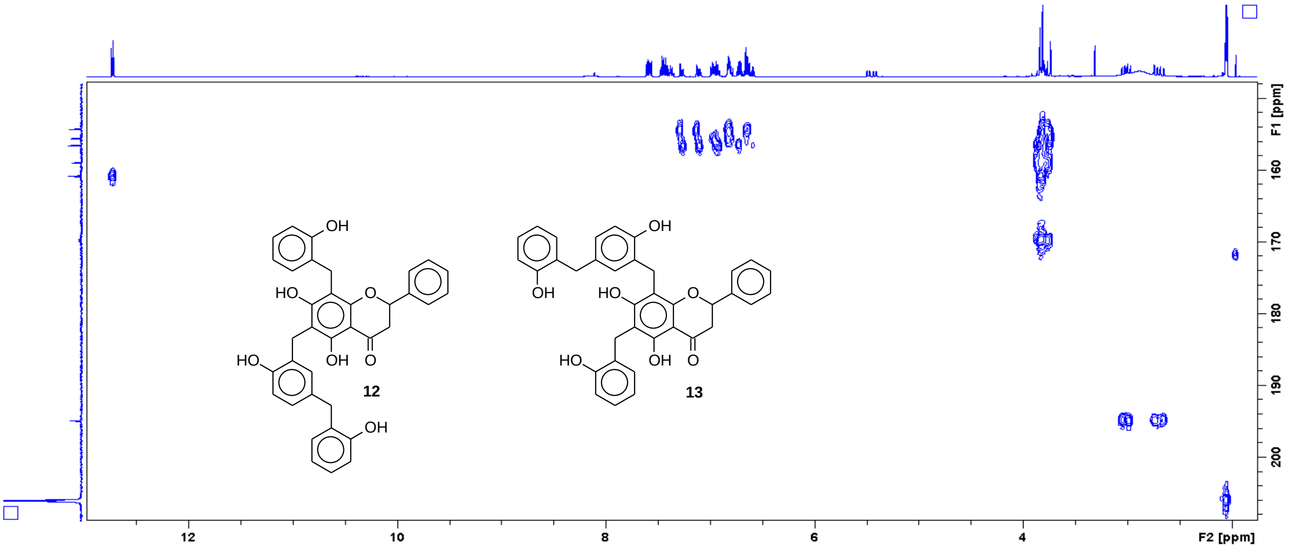

Figure S122. *Expansion of the long-range* ^{1}H-^{13}C *correlation map from HMBC NMR experiment of the mixture of* ***uvarinol*** *(**12**) and* ***isouvarinol*** *(**13**) in acetone-*d_6 *at 500 (*^{1}H*) and 125 MHz (*^{13}C*), respectively.*

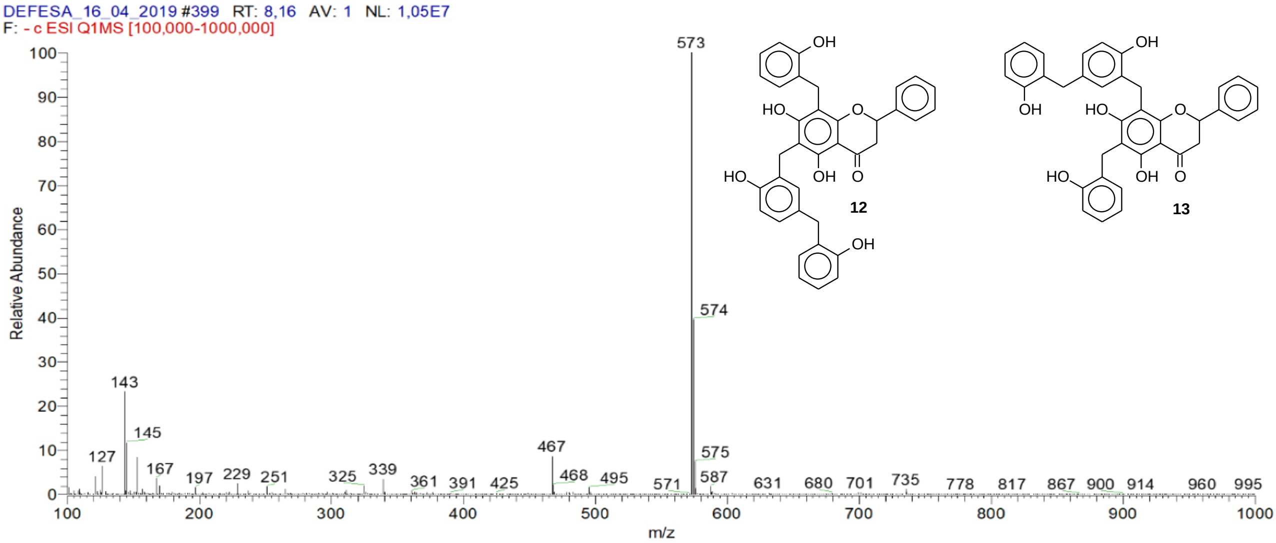

Figure S123. *LR-ESI(–)-MS spectrum of the mixture of* ***uvarinol*** *(**12**) and* ***isouvarinol*** *(**13**) (m/z 573 [M–H]*$^{-}$*).*
